# Chemical Synthesis of Glycopeptides containing l-Arabinosylated Hydroxyproline and Sulfated Tyrosine

**DOI:** 10.1021/acs.orglett.3c00411

**Published:** 2023-03-14

**Authors:** Jasper W. van de Sande, Bauke Albada

**Affiliations:** Laboratory of Organic Chemistry, Wageningen University & Research, Stippeneng 4, 6708 WE Wageningen, The Netherlands

## Abstract

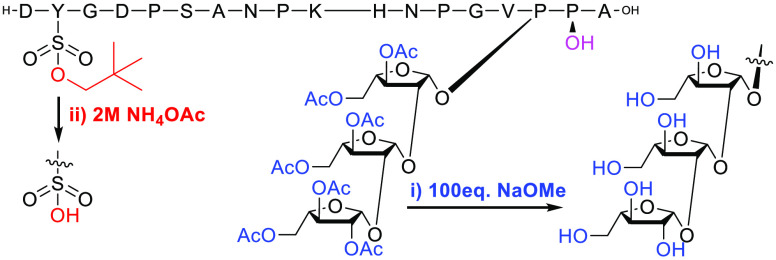

Post-translationally modified peptides are important regulating molecules for living organisms. Here, we report the stereoselective total synthesis of β-1,2-linked l-arabinosylated Fmoc-protected hydroxyproline building blocks and their incorporation, together with sulfated tyrosine and hydroxyproline, into the plant peptide hormone PSY1. Clean glycopeptides were obtained by performing acetyl removal from the l-arabinose groups prior to deprotection of the neopentyl-protected sulfated tyrosine.

Cell-to-cell communication by excreted peptides is pivotal for many regulatory mechanisms of living organisms.^1−4^ For example, plant peptide hormones (PPHs) are an important class of signaling and regulating molecules for plant growth, developmental processes and defense responses.^5^ Whereas details of these mechanisms have been established for simple peptides, a major fraction of PPHs are chemically modified, which are more difficult to obtain and study.^6^ Prominent plant-related post-translational modifications (PTMs) are proline hydroxylation, tyrosine sulfation, and hydroxyproline arabinosylation.^7^ As some of these chemical modifications are poorly compatible with current synthetic approaches, details of the interaction with their corresponding receptors, such as leucine-rich repeat receptor-like kinases (LRR-RLKs), are yet to be elucidated.^8,9^ As such, effects of this peptide-receptor interaction for intracellular signaling pathways remain poorly understood.

In this paper, we describe the synthesis of mono-, di-, and triarabinosylated hydroxyproline building blocks in which the l-arabinose units are linked via linear β-1,2-linkages, their incorporation in the 18 amino acid long plant peptide hormone PSY1,^10,11^ and the optimal deprotection protocol that leads to the fully unprotected peptide (Figure 1). As this peptide is a member of the PSY peptide family that is found in all higher plants and mosses,^12^ we expect that this synthesis will lead to PPHs and derivatives that increase our understanding of the intercellular communication of such organisms and, in particular, the role of PTMs in these processes.^13^ Here, we reveal a strategy to prepare more complex peptides than the ones reported so far,^14^ showing compatibility of the 1,2-*cis*-glycosidic connectivity between attached carbohydrates and amino acid unit with the synthetic incorporation of other PTMs in peptides.

**Figure 1:**
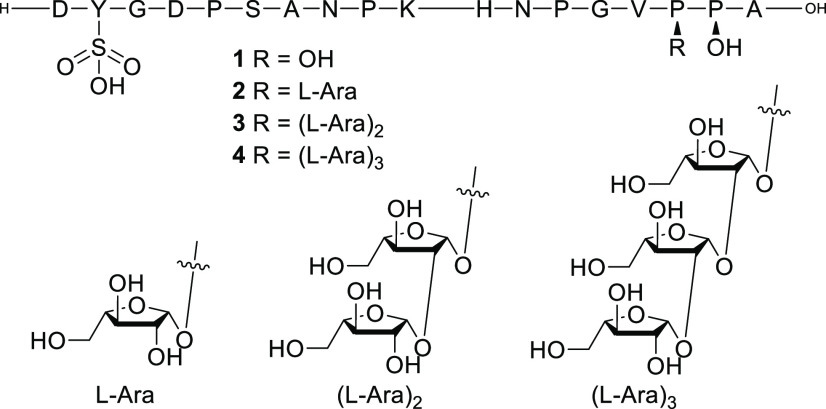
Structure of *Brassica* PSY1 containing hydroxylated proline **1**, or mono-, di-, or triarabinosylated hydroxyproline **2**, **3**, and **4**, respectively.

It was envisioned that synthesis of the polyamide backbone of PSY1 would be accessible with Fmoc/*t*Bu-based solid-phase peptide synthesis (SPPS) using the appropriate building blocks, i.e., Fmoc-Hyp(*t*Bu)–OH, Fmoc-Tyr(SO_3_Np)–OH, and Fmoc-Hyp[l-Ara_3_(OAc)_7_]–OH.^15^ Whereas the first two are obtained from commercial sources, the arabinosylated hydroxyproline derivative could be synthesized by a repetition of stereoselective glycosylation reactions using hydroxyproline and the proper protected arabinose donor. To achieve that, we apply a previously reported glycosylation strategy that involves the use of mild activator iodonium dicollidine perchlorate (IDCP) in order to stereoselectively couple the arabinose donor to Fmoc-Hyp-OBn.^15^

Synthesis of β-1,2-linked triarabinosylated PSY1 started from the generation of the arabinose donor (Scheme 1a) and the 4-hydroxyproline acceptor (Scheme 1b). For the synthesis of the arabinose donor we converted l-arabinose **5** into its corresponding furanoside by Fischer glycosylation, which then underwent a thioglycosylation to form ethyl thioarabinofuranoside **6**. Simultaneous protection of the 3- and 5-OH of furanoside **6** with 1,3-dichloro-1,1,3,3-tetraisopropyldisiloxane (TIPDS) was achieved, after which the 2-OH was protected using freshly prepared *p*-methoxybenzyl (PMB) bromide, to form intermediate **7**. We found reported procedures that used commercially available PMB bromide were ineffective.^16^ However, using freshly prepared PMB bromide (see ESI) for installation of the PMB moiety at C2–O was successful (52% yield). Lastly, the TIPDS protecting group was removed and replaced with benzyl groups by treatment of **7** with tetrabutylammonium fluoride followed by benzylation using benzyl bromide and NaH to obtain arabinose donor **8**. In this step, both l-α (**8**) and l-β (**S4**) arabinofuranoside were obtained (ratio 3.25:1; determined by ^1^H NMR analysis of both isolated compounds, Figure S1). Specifically, l-α-arabinofuranoside **8** was identified by a doublet of the anomeric proton at 5.31 ppm with a *J*-coupling constant of 2.38 Hz, whereas the anomeric proton of l-β arabinofuranoside **S4** was found at 5.35 ppm with a *J*-coupling constant of 4.94 Hz.^17^ Formation of both anomers most probably originated from a yet not understood furanose-ring mutarotation process in which the thioethyl group inverts.^18^ Importantly, both enantiomers led to the same 1,2-*cis*-linked product when applied in the next step (*vide infra*).

**Scheme 1:**
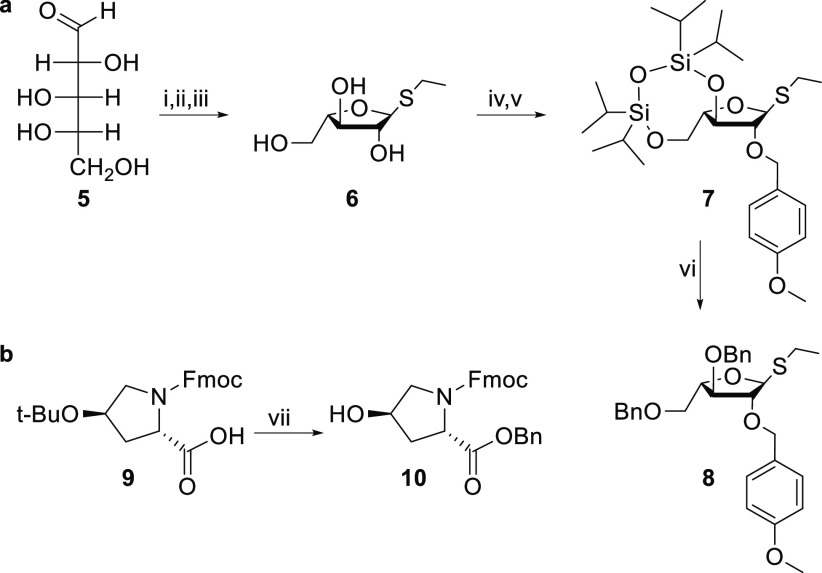
Synthesis of Arabinose Donor **8** (a) and Hydroxyproline Acceptor **10** (b) for the synthesis of AraHyp Reagents and conditions: (i) 1) MeOH, AcCl, 2) BzCl, pyridine, 50%; (ii) EtSH, BF_3_·Et_2_O, DCM, 81%; (iii) NaOMe, MeOH, 96%; (iv) TIPDSiCl_2_, pyridine, 84%; (v) PMBBr, NaH, THF, 52%; (vi) 1) TBAF, THF, 2) BnBr, NaH, THF, 87%; (vii) 1) BnOH, DMAP, EDC·HCl, DCM, 0 °C, 2) TFA, 77%.

Fmoc-Hyp-OBn **10** was obtained from commercially available Fmoc-Hyp(*t*Bu)–OH **9** that was first benzylated on the carboxylic acid moiety and subsequently subjected to *tert*-butyl removal using TFA, providing the secondary alcohol.

With arabinose donor **8** and hydroxyproline acceptor **10** in hand, stereoselective 1,2-*cis* glycosylation was performed to monoarabinosylate hydroxyproline (Scheme 2). As the intramolecular aglycon delivery (IAD) reaction of the acetal formed between hydroxyproline **10** and either donor **8** or donor **S4** led to the same 1,2-*cis*-linked product, the absolute stereochemistry at the anomeric center of precursor does not affect the outcome of this glycosylation method. To be more specific, this IAD glycosylation method involves an approach in which the *p*-methoxybenzyl group acts as the initial transient attachment point for the acceptor, in this case hydroxyproline **10**. Upon activation of the thioglycoside using IDCP, which forms an *sp*^2^ hybridized C1 atom, the glycosyl donor is relocated to C1 from the same face as C2–O and the 1,2-*cis*-glycosyl bond is formed stereoselectively (Figure 2).

**Figure 2:**
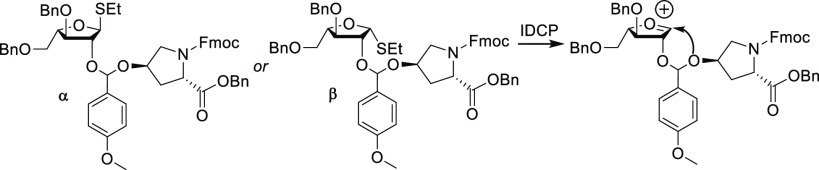
Activation of two enantiomers leads to the same intermediate, and one product is obtained from the subsequent intramolecular aglycon delivery (AID).

**Scheme 2:**
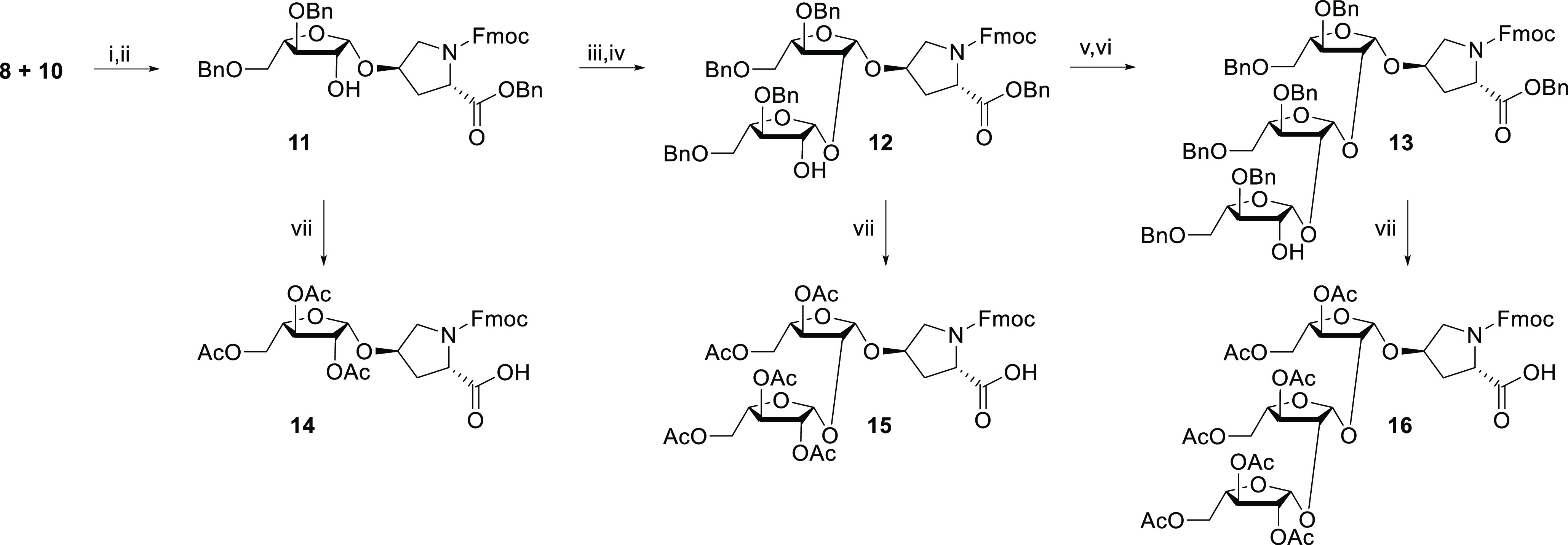
Synthesis of Fmoc- and Ac-Protected Mono-, Di-, and Triarabinosylated Hydroxyproline Reagents and conditions: (i) DDQ, DCM, 60%; (ii) IDCP, DCM, 74%; (iii) arabinose donor **8**, DDQ, DCM, 43%; (iv) IDCP, DCM, 63%; (v) arabinose donor **8**, DDQ, DCM, 53%; (vi) IDCP, DCM, 35%; (vii) Pd(OH)_2_/C, H_2_, DCM/MeOH/AcOH, then Fmoc-OSu, NaHCO_3_, H_2_O/1,4-dioxane/acetone, then Ac_2_O, pyridine, 66% (**14**), 59% (**15**), 58% (**16**). Yields over three steps are reported.

As such, treatment of donor **8** and acceptor **10** with 2,3-dichloro-5,6-dicyanobenzoquinone (DDQ) led to the transient mixed acetal intermediate. Subsequently, freshly prepared IDCP was used to activate the IAD mechanism to afford β-linked hydroxyproline monoarabinofuranoside **11**. The resulting free 2-OH of **11** was reacted with arabinose donor **8** using DDQ to form the transient mixed acetal, which was converted into the β-1,2-linked hydroxyproline diarabinofuranoside **12** by IDCP-initiated IAD. Similarly, treatment of **12** with arabinose donor **8** and the aforementioned reagents resulted in β-1,2-linked hydroxyproline triarabinofuranoside **13**.

Prior to their application in SPPS, the various arabinosylated hydroxyproline building blocks that were collected along the previously described synthesis path were converted into Fmoc-protected peracetylated amino acid constructs. For this, the three AraHyp moieties **11**–**13** were debenzylated in order to liberate carboxylic acid for peptide coupling, and to exchange the glycosyl *O*-benzyl groups with *O*-acetyl groups.^15^ Consequently, Fmoc-Hyp[Ara(OBn)]_1_-OBn **11**, Fmoc-Hyp[Ara(OBn)]_2_-OBn **12**, and Fmoc-Hyp[Ara(OBn)]_3_-OBn **13** were subjected to hydrogenolysis using Pd(OH_2_)/C and H_2_ gas.^15^ Interestingly, striking differences in debenzylation rates were observed for the different monomers. Whereas debenzylation of Ara_1_Hyp **11** required multiple Pd(OH)_2_/C refreshing and extended reaction times (48 h), the di- and triarabinosylated building blocks did not require refreshing of Pd(OH)_2_/C and were completed overnight. Also, whereas debenzylation of **11** and **13** was accompanied by significant Fmoc-removal, this was much less the case for **12**.

Fortunately, removal of the fluorenylmethyloxycarbonyl (Fmoc) protecting group from the secondary amine could be remedied by treatment of the product with Fmoc-OSu in the presence of sodium bicarbonate in order to protect the amino functionality again. After protection of the glycosyl OH groups with acetyl groups using acetic anhydride and pyridine, the desired mono-, di-, and triarabinosylated hydroxyproline building blocks Fmoc-Hyp[Ara(OAc)]_1_–OH **14**, Fmoc-Hyp[Ara(OAc)]_2_–OH **15**, and Fmoc-Hyp[Ara(OAc)]_3_–OH **16**, were obtained in yields of 66%, 59%, and 58%, respectively. Flash column chromatography purification of the arabinosylated building blocks resulted in higher yields of pure compound, when compared to previous reported procedure involving reverse-phase HPLC purification.^15^

With these Fmoc/Ac-protected arabinosylated hydroxyproline building blocks in hand, SPPS of the *Brassica* PSY1 peptide was commenced (Scheme 3). Starting with Fmoc-Ala bound to a Wang resin (loading: 0.68 mmol/g), the peptide chain was elongated with Fmoc-Hyp(*t*Bu)–OH. For coupling of the arabinosylated hydroxyproline (**14**, **15**, or **16**) monomers we used the coupling reagents 2-(1H-benzotriazole-1-yl)-1,1,3,3-tetramethyl-uronium hexafluorophosphate (HBTU) and 1-hydroxy-benzotriazole (HOBt). Introduction of arabinosylated Fmoc-Hyp **14**–**16** to the dipeptide was monitored by ninhydrin/chloranil tests and high-resolution mass spectrometry (HRMS) after cleavage of small aliquots of resins. As we used a slight excess of resin-bound amine groups with respect to the arabinosylated building blocks, remaining amine-groups were permanently acetylated using Ac_2_O and pyridine. After this, Fmoc-Val-OH coupling was conducted manually in order to monitor coupling of this beta-substituted amino acid to the different sterically hindered glycosylated tripeptides. Once formation of tetrapeptides **17**–**19** was confirmed, subsequent chain elongation was conducted using an automated peptide synthesizer. After acidic cleavage from the resin, the crude (glyco)peptides were purified using reversed-phase preparative-HPLC, yielding analytically pure Np- and Ac-protected (glyco)peptides **20**–**23** (see ESI).

**Scheme 3:**
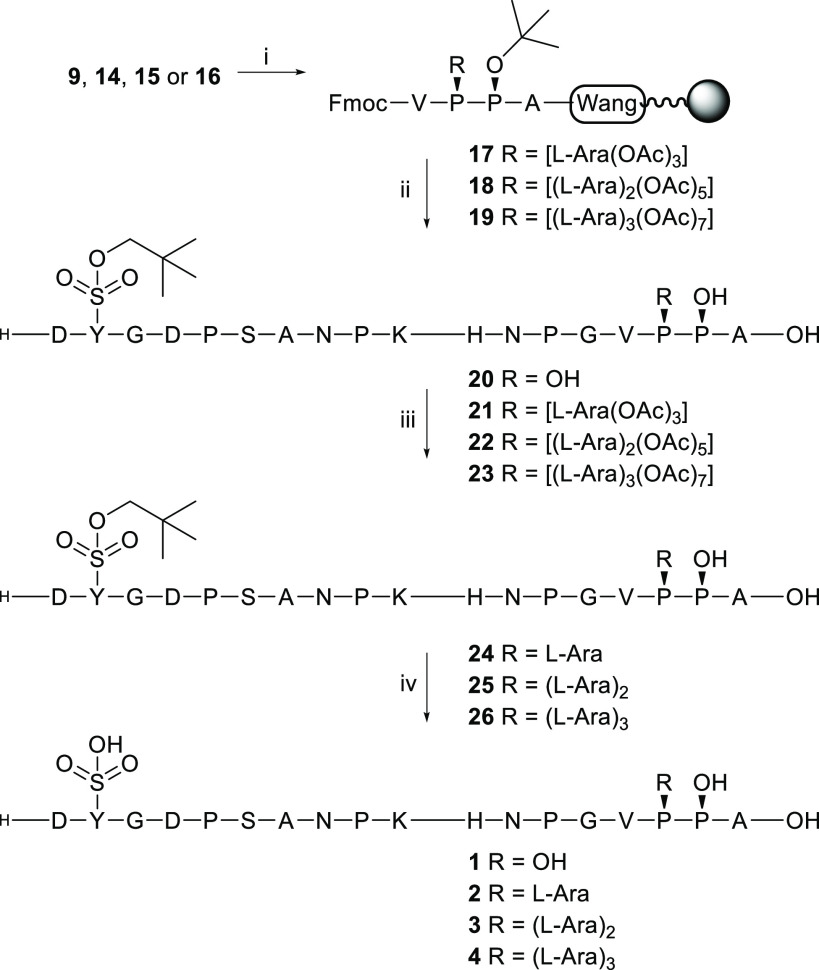
Synthesis of *Brassica* PSY1 Glycopeptide Reagents and conditions: (i) SPPS by hand using HBTU/HOBt as coupling reagents, DIPEA, DMF, coupling was monitored by microcleavage of a small portion of the resin with 95% TFA, followed by HRMS analysis; (ii) peptide synthesis, acidic cleavage, and RP-HPLC purification; (iii) NaOMe, MeOH; (iv) 2 M NH_4_OAc.

Once the octadeca-(glyco)peptides were prepared, we focused on removal of the two acid-stable protecting groups, namely, neopentyl (Np) at the 2-Tyr(SO_3_Np) and acetyl groups at the 16-Hyp[Ara(OAc)]_0–3_ (**21**–**23**). For the nonarabinosylated control peptide PSY1 16-Hyp **20** only Np removal in 2 M ammonium acetate solution fulfills the complete synthesis of this peptide resulting in *Brassica* PSY1 16-Hyp **1**. However, for glycopeptides **21**–**23** the order of deprotection was found important. Specifically, starting with Np removal from acetylated glycopeptide **21** using a 2 M ammonium acetate solution resulted in multiple peaks in liquid chromatography trace, only one of which corresponded to the target compound (Figure 3). Subsequent treatment with deacetylation reagents did not provide the correct mass of the desired glycopeptide. Fortunately, switching the order by first deacetylation using sodium methoxide in methanol (and lyophilization to obtain glycopeptides **24**–**26**) and subsequent removal of the tyrosine sulfate Np protecting group with 2 M ammonium acetate afforded the desired PSY1 peptides 16-Ara_1_Hyp **2** (see Figure 3), 16-Ara_2_Hyp **3**, and 16-Ara_3_Hyp **4** in high purity. LC-MS analyses of these constructs revealed the presence of rotational isomers, especially for the deacetylated precursors (see Figures S6.1, S7.1, S8.1 and S10.1, S11.1, S12.1).

**Figure 3:**
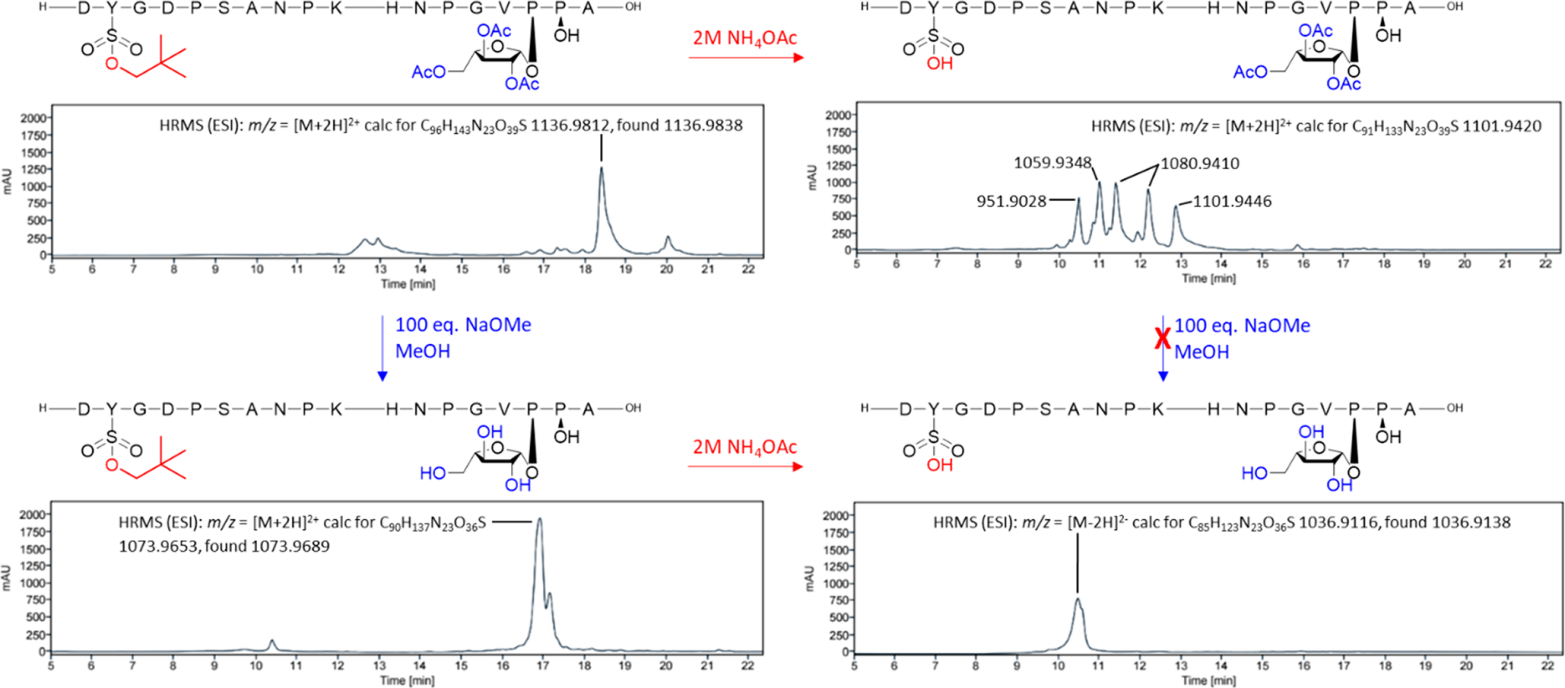
RP-HPLC analysis of the reaction mixtures obtained during the various deprotection paths for PSY1 16-Ara_1_Hyp **21**. The shoulder at *t*_R_ = 17.1 min in the HPLC trace in the lower left corner is associated with the same *m*/*z* value as the main peak, indicating the presence of rotational isomers for glycopeptide **24**. The optimal procedure entails removal of the acetyl-groups from the arabinose prior to removal of the Np group from the tyrosine sulfate group.

To conclude,^19^ we report the synthesis of the triple-modified plant peptide hormone PSY1, which is a glycopeptide that contains β-1,2-linked arabinose carbohydrates in combination with a hydroxyproline and sulfated tyrosine. Stereoselective glycosylation of hydroxyproline with l-arabinofuranoside was achieved by intramolecular aglycon delivery (IAD) using a PMB ether on the C2 hydroxyl group. This resulted in three Fmoc-protected building blocks that are compatible with Fmoc-based solid-phase peptide synthesis (SPPS) using HBTU/HOBt as coupling reagents for the introduction of Fmoc-Hyp[(l-Ara)]_1–3_–OH **14**–**16** and Fmoc-Hyp(*t*Bu)–OH **9**. Elongation to the PSY1 (glyco)peptide to form derivatives **20**–**23** was done using an automated peptide synthesizer. Target peptides were obtained by initial removal of acetyl groups and subsequent Np removal. With this, we disclose a robust synthesis approach for the preparation of complex post-translationally modified peptides, especially those containing a sulfated tyrosine and arabinosylated hydroxyproline units, that will be useful for the preparation of peptides for phenotypical biological evaluation, such as their role in intercellular signaling.

## Data Availability

The data underlying this study are available in the published article and its Supporting Information. The preprint of this paper is available from ChemRxiv.^19^

## Supporting Information Available

Experimental details, compound characterization, NMR spectra, and analytical data (PDF)
