# Supplementary material for: Chemical Synthesis of Glycopeptides containing l-Arabinosylated Hydroxyproline and Sulfated Tyrosine
Source: Org Lett. 2023 Mar 14;25(11):1907–11. doi: 10.1021/acs.orglett.3c00411 (PMC10043930; doi:10.1021/acs.orglett.3c00411)
Supplement: Supplementary file 1 — ol3c00411_si_001.pdf [file ol3c00411_si_001.pdf]

Electronic Supplementary Information for

Chemical Synthesis of Glycopeptides containing L-Arabinosylated Hydroxyproline and Sulfated Tyrosine

Jasper W. van de Sande and Bauke Albada*

Laboratory of Organic Chemistry, Wageningen University & Research, Stippeneng 4, 6708 WE Wageningen, The Netherlands

Table of Contents

General procedures S3

General procedure for (glyco)peptide synthesis S3

Synthesis of arabinosedonor (**8**) and hydroxyproline acceptor (**10**) building blocks S5

Methyl 2,3,5-tri-O-benzoyl-α-L-arabinofuranoside (**S1**) S5

Ethyl 2,3,5,-tri-O-benzoyl-1-thio-α-L-arabinofuranoside (**S2**) S5

Ethyl 1-thio-α-L-arabinofuranoside (**6**) S6

Ethyl 3,5-O-TIPS-1-thio-α-L-arabinofuranoside (**S3**) S6

Ethyl 2-O-PMB-3,5-O-TIPS-1-thio-α-L-arabinofuranoside (**7**) S6

Ethyl 2-O-PMB-3,5-O-benzyl-1-thio-α-L-arabinofuranoside (**8**) S6

Ethyl 2-O-PMB-3,5-O-benzyl-1-thio-β-L-arabinofuranoside (**S4**) S6

Fmoc-Hyp-OBn (**10**) S7

1-(bromomethyl)-4-methoxybenzene (**S5**) S7

Synthesis of mono-, di-, and triarabinosylated hydroxyproline (**14-16**) S8

Iodonium di-collidine perchlorate (IDCP) (**S6**) S8

Fmoc-[(3,5-Bn)Ara$_1$]-2-PMB acetal Hyp-OBn (**S7**) S9

Fmoc-[(3,5-Bn)Ara$_1$]Hyp-OBn (**11**) S9

Fmoc-[(3,5-Bn)Ara$_2$]-2-PMB acetal Hyp-OBn (**S8**) S9

Fmoc-[(3,5-Bn)Ara$_2$]Hyp-OBn (**12**) S10

Fmoc-[(3,5-Bn)Ara$_3$]-2-PMB acetal Hyp-OBn (**S9**) S10

Fmoc-[(3,5-Bn)Ara$_3$]Hyp-OBn (**13**) S11

Fmoc-[Ara(OAc)]$_1$Hyp-OBn (**14**) S11

Fmoc-[Ara(OAc)]$_2$Hyp-OBn (**15**) S11

Fmoc-[Ara(OAc)]$_3$Hyp-OBn (**16**) S12

Figure S1. ^{1}H NMR spectra of furanose protons from anomers **8** & **S4** S13

Synthesis and HPLC/MS data of *Brassica* PSY1 glycopeptides (**1-4**) S14

Fmoc-V-Hyp[Ara(OAc)]$_1$-Hyp(*tBu*)-A-Wang resin (**17**) S14

Fmoc-V-Hyp[Ara(OAc)]$_2$-Hyp(*tBu*)-A-Wang resin (**18**) S14

Fmoc-V-Hyp[Ara(OAc)]$_3$-Hyp(*tBu*)-A-Wang resin (**19**) S14

H-Asp-Tyr(SO$_2$ONp)-Gly-Asp-Pro-Ser-Ala-Asn-Pro-Lys-His-Asn-Pro-Gly-Val-Hyp-Hyp-Ala-OH (**20**) S15

H-Asp-Tyr(SO$_2$ONp)-Gly-Asp-Pro-Ser-Ala-Asn-Pro-Lys-His-Asn-Pro-Gly-Val-Hyp[Ara(OAc)]$_1$-Hyp-Ala-OH (**21**) S16

H-Asp-Tyr(SO$_2$ONp)-Gly-Asp-Pro-Ser-Ala-Asn-Pro-Lys-His-Asn-Pro-Gly-Val-Hyp[Ara(OAc)]$_2$-Hyp-Ala-OH (**22**) S17

H-Asp-Tyr(SO_2ONp)-Gly-Asp-Pro-Ser-Ala-Asn-Pro-Lys-His-Asn-Pro-Gly-Val-Hyp[Ara(OAc)]$_3$-Hyp-Ala-OH (**23**) S18
H-Asp-Tyr(SO_2ONp)-Gly-Asp-Pro-Ser-Ala-Asn-Pro-Lys-His-Asn-Pro-Gly-Val-Hyp[Ara$_1$]-Hyp-Ala-OH (**24**) S19
H-Asp-Tyr(SO_2ONp)-Gly-Asp-Pro-Ser-Ala-Asn-Pro-Lys-His-Asn-Pro-Gly-Val-Hyp[Ara$_2$]-Hyp-Ala-OH (**25**) S20
H-Asp-Tyr(SO_2ONp)-Gly-Asp-Pro-Ser-Ala-Asn-Pro-Lys-His-Asn-Pro-Gly-Val-Hyp[Ara$_3$]-Hyp-Ala-OH (**26**) S21
H-Asp-Tyr(SO_3H)-Gly-Asp-Pro-Ser-Ala-Asn-Pro-Lys-His-Asn-Pro-Gly-Val-Hyp-Hyp-Ala-OH (**1**) S22
H-Asp-Tyr(SO_3H)-Gly-Asp-Pro-Ser-Ala-Asn-Pro-Lys-His-Asn-Pro-Gly-Val-Hyp[Ara$_1$]-Hyp-Ala-OH (**2**) S23
H-Asp-Tyr(SO_3H)-Gly-Asp-Pro-Ser-Ala-Asn-Pro-Lys-His-Asn-Pro-Gly-Val-Hyp[Ara$_2$]-Hyp-Ala-OH (**3**) S24
H-Asp-Tyr(SO_3H)-Gly-Asp-Pro-Ser-Ala-Asn-Pro-Lys-His-Asn-Pro-Gly-Val-Hyp[Ara$_3$]-Hyp-Ala-OH (**4**) S25
^{1}H and ^{13}C NMR spectra of all compounds S26
^{1}H and ^{13}C spectra of Methyl 2,3,5-tri-O-benzoyl-α-L-arabinofuranoside (**S1**) S26
^{1}H and ^{13}C spectra of Ethyl 2,3,5,-tri-O-benzoyl-1-thio-α-L-arabinofuranoside (**S2**) S27
^{1}H and ^{13}C spectra of Ethyl 1-thio-α-L-arabinofuranoside (**6**) S28
^{1}H and ^{13}C APT spectra of Ethyl 3,5-O-TIPS-1-thio-α-L-arabinofuranoside (**S3**) S29
^{1}H and ^{13}C APT spectra of Ethyl 2-O-PMB-3,5-O-TIPS-1-thio-α-L-arabinofuranoside (**7**) S30
^{1}H and ^{13}C APT spectra of Ethyl 2-O-PMB-3,5-O-benzyl-1-thio-α-L-arabinofuranoside (**8**) S31
^{1}H and ^{13}C spectra of Ethyl 2-O-PMB-3,5-O-benzyl-1-thio-β-L-arabinofuranoside (**S4**) S32
^{1}H and ^{13}C spectra of Fmoc-Hyp-OBn (**10**) S33
^{1}H and ^{13}C spectra of 1-(bromomethyl)-4-methoxybenzene (**S5**) S34
^{1}H spectrum of Iodonium di-collidine perchlorate (IDCP) (**S6**) S35
^{1}H and ^{13}C APT spectra of Fmoc-[(3,5-Bn)Ara$_1$]-2-PMB acetal Hyp-OBn (**S7**) S36
^{1}H and ^{13}C APT spectra of Fmoc-[(3,5-Bn)Ara$_1$]Hyp-OBn (**11**) S37
^{1}H and ^{13}C spectra of Fmoc-[(3,5-Bn)Ara$_2$]-2-PMB acetal Hyp-OBn (**S8**) S38
^{1}H and ^{13}C spectra of Fmoc-[(3,5-Bn)Ara$_2$]Hyp-OBn (**12**) S39
^{1}H and ^{13}C spectra of Fmoc-[(3,5-Bn)Ara$_3$]-2-PMB acetal Hyp-OBn (**S9**) S40
^{1}H and ^{13}C spectra of Fmoc-[(3,5-Bn)Ara$_3$]Hyp-OBn (**13**) S41
^{1}H and ^{13}C APT spectra of Fmoc-[Ara(OAc)]$_1$Hyp-OBn (**14**) S42
^{1}H and ^{13}C spectra of Fmoc-[Ara(OAc)]$_2$Hyp-OBn (**15**) S43
^{1}H and ^{13}C spectra of Fmoc-[Ara(OAc)]$_3$Hyp-OBn (**16**) S44

General procedures

Starting materials, reagents, and solvents were purchased from commercial vendors and used as received unless stated otherwise. Anhydrous CH_2Cl_2 and anhydrous THF were obtained from a PureSolv Solvent Purification System. Traces of water from reagents were removed by co-evaporation with toluene in reactions that required anhydrous conditions. Molecular sieves (4 Å) were activated at 120 °C under reduced pressure in a drying oven (Binder). Reactions were monitored by thin-layer chromatography (TLC) using Merck aluminum sheets (Silica gel 60 F_{254}) with detection by UV absorption (254 nm), by spraying with a solution of 10% sulfuric acid in MeOH, or a solution of $KMnO_4$ (10 g/L) and K_2CO_3 (50 g/L) in water, followed by charring at ~150 °C. Organic solvents were removed under reduced pressure at 40 °C. Flash column chromatography was performed using SiliaFlash P60 silica gel (particle size of 40–63 µm, pore diameter of 60 Å) with the indicated eluents. ^1H NMR and ^{13}C NMR spectra were recorded using a Brüker AV-400 (400 and 101 MHz, respectively) spectrometer in the given deuterated solvent. Chemical shifts are given in ppm (δ) relative to the residual solvent peak or tetramethyl silane (0 ppm) as internal standard and coupling constants are given in Hz. Multiplicity is reported as s: singlet, d: doublet, dd: doublet of doublets, td: triplet of doublets, qd: quartet of doublets, ddd: doublet of doublet of doublets, dtd: doublet of triplet of doublets, t: triplet, tt: triplet of triplets, dt: doublet of triplets, q: quartet, dq: doublet of quartets, m: multiplet. Assignments were made by standard COSY and HSQC. High-resolution mass spectrometry (HRMS) analysis was performed with an Exactive Plus Orbitrap Mass Spectrometer (Thermo Fisher), equipped with an electrospray ion source (ESI) in positive mode. MS-grade methanol was used as eluent. The high-resolution mass spectrometer was calibrated prior to measurements with a calibration mixture (Thermo Finnigan).

General procedure for (glyco)peptide synthesis

Peptide synthesis was performed following Fmoc Solid-Phase Peptide Synthesis (SPPS) strategy. Chain elongation initiated with Fmoc-Ala-Wang resin 100–200 mesh Novabiochem®, a *p*-alkoxy-benzyl alcohol polymer-bound (polystyerene-1%, DVB) amino acid (loading capacity 0.68 mmol/g). Introduction of new amino acids were either performed by manual synthesis or in an automatic peptide synthesizer (CS336X Peptide Synthesizer CS BIO Co.). For automated SPPS, a standard protocol was used in which every coupling was reacted for 3 h. 2-(1H-benzotriazole-1-yl)-1,1,3,3-tetramethyluronium hexafluorophosphate (HBTU) and 1-hydroxybenzotriazole (HOBt) were employed as coupling reagents. Manual addition of amino acids was performed as follow: The resin was pre-swollen with *N*-methyl-2-pyrrolidone (NMP). Fmoc removal was achieved with 20% piperidine in *N,N*-dimethylformamide (DMF, 2 x 8 min). Then the resin was washed with DMF (3 x 2 min). Fmoc-amino acid or Fmoc-glyco amino acid was activated with HBTU, HOBt, and *N,N*-diisopropylethylamine (DIPEA) in DMF for 2 min before being added to the resin. The reaction mixture was allowed to couple for 2 h (except for secondary amine or glyco amino acid, these were coupled for 16 h). The reaction was monitored by resin staining using ninhydrin (15 g/L, supplemented with 30 mL/L acetic acid in *n*-butanol) for primary amines, or performing a chloranil test (20 mL/L acetaldehyde in DMF as reagent A and 20 g/L *p*-chloranil in DMF as reagent B) for secondary amines, or by cleavage of a small amount of resin using 95% trifluoroacetic acid (TFA), 2.5% triisopropylsilane, 2.5% MilliQ (deionised water, produced with a Milli-Q Integral 3 system; Millipore, Molsheim/France) for 2 hours. Redissolved cleaved product was then analyzed with a Q Exactive Focus UHPLC-MS (Agilent 1290 UHPLC). Upon completion, the resin was washed with DMF (3 x 2 min) and deprotected by 20% piperidine in DMF (2 x 8 min). After washing again with DMF (3 x 2 min), the next amino acid was coupled. Coupling and deprotection were repeated until the desired peptide was obtained. After either manual or automated SPPS, the peptide was cleaved from the resin by treatment with a cocktail of 95% TFA, 2.5% MilliQ, 2.5% TIS for 2 h. Each cleaved peptide resin was then washed extensively with fresh cleavage cocktail. The peptide was precipitated by addition of cold diethyl ether (1:1 ether:hexane, 10x the initial cocktail volume) and centrifuged for 10 min at 6000 rpm. The supernatant was discarded and the precipitate was washed with cold diethyl ether and again centrifuged for 10 min at 6000 rpm. The resulting precipitate was dried in a light stream of N_2, redissolved in acetonitrile (ACN):MilliQ (2:8) and then lyophilized (Labconco FreeZone lyophilizer, 2.5 L, -84 °C, connected to a 35_i xDS Edwards Oil-Free Dry Scroll Pump). The lyophilized (O-acetylated glyco)peptides were purified by semi-preparative reverse phase HPLC (Agilent 1260 Preparative HPLC with a DAD G7115A and MSD) using a semi-preparative Zorbax Eclipse column (XDB-C18, 9.4 x 250 mm, 5-Micron) followed by lyophilization of the product. Peptides were further treated for any deprotections accordingly described for each peptide. The purity of synthesized peptides was analyzed by high-performance liquid chromatography (HPLC) coupled to mass spectrometry (ESI-MS, measuring both positive and negative in a switch mode) using a Q Exactive Focus Agilent 1290 Infinity UHPLC-MS system or a LXQ Mass Spectrometer with HPLC (Thermo Finnigan). The HPLC system is equipped with a diode array detector (DAD G4212A, at 415 and 454 nm) and a Dr. Maisch ReproSil Gold 120 C18, 3 µm, 200 x 3 mm column containing a 10 mm guard with a flow rate of 0.4 mL/min. For peptides **21-26** the eluent contained 0.1% FA in H_2O-ACN with a gradient of 5→5→95→95→5→5%

(percentage CH_3CN) (0→5→25→30→35→40 min). For peptides **1-4** the eluent contained 10 mM NH_4OAc in H_2O-ACN with a gradient of 5→5→60→60→5→5% (percentage CH_3CN) (0→5→25→30→35→40 min).

Synthesis of arabinosedonor (8) and hydroxyproline acceptor (10) building blocks

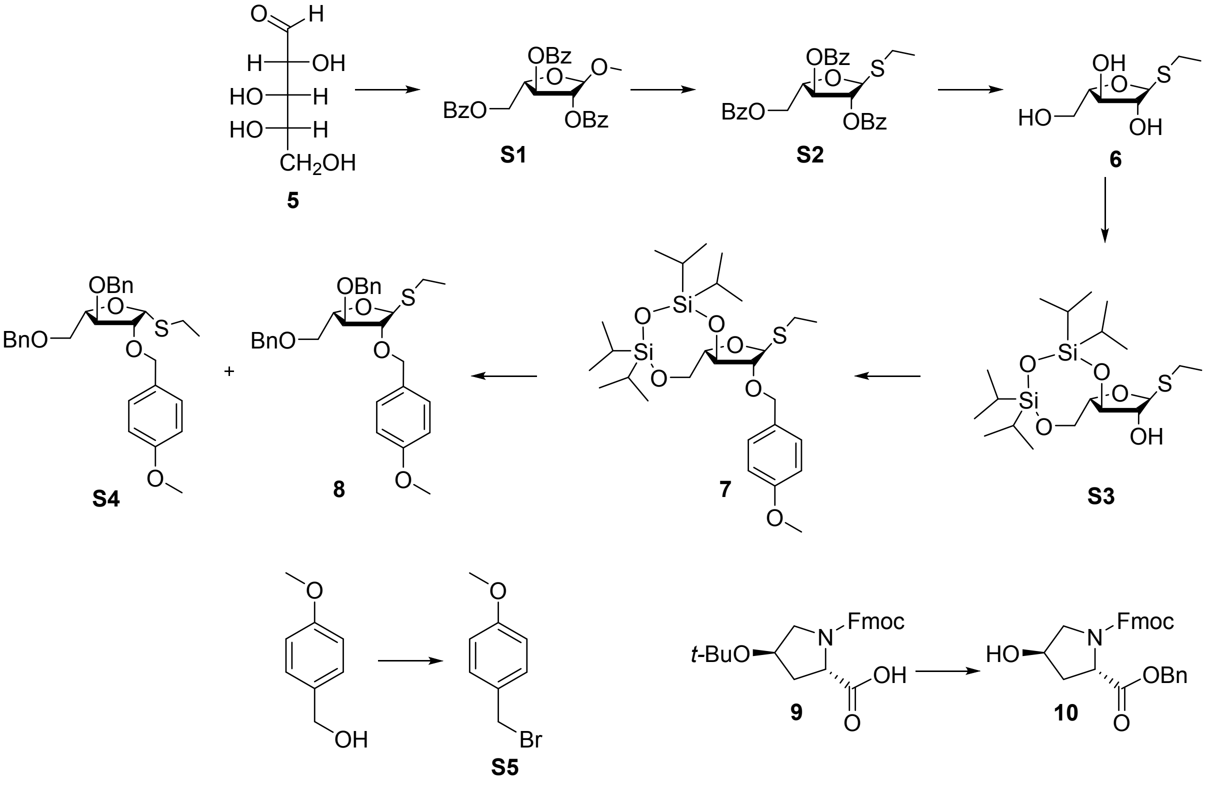

Methyl 2,3,5-tri-O-benzoyl-α-L-arabinofuranoside (S1)

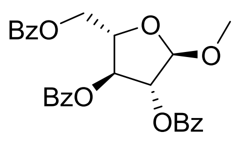

L-arabinose **5** (20 g, 133 mmol) was suspended in methanol (400 mL) and a freshly prepared mixture of acetylchloride (12 mL) in methanol (100 mL) was added dropwise at 0 °C. After stirring for 3 hrs at room temperature, the reaction was quenched with pyridine till pH 7. The mixture was concentrated and the crude was dissolved in pyridine (160 mL). Benzoylchloride (77 mL, 663 mmol) was added dropwise during 25 min at 0 °C. The reaction mixture was stirred overnight. Upon reaction completion, the mixture was diluted with H_2O while cooled under stirring. Organic products were extracted from the mixture with CH_2Cl_2 (2 x 250 mL), after which the organic layer was washed with H_2O (3 x 200 mL), 3N H_2SO_4 (3 x 150 mL), saturated $NaHCO_3$ (3 x 200 mL), brine (1 x 250 mL). Then, the organic phase was dried over $MgSO_4$, filtrated and concentrated. The product was crystallized with the use of ethanol, to afford arabinofuranoside **5** as white crystals (31.7 g, 66.5 mmol, 50%). ^1H NMR (400 MHz, $CDCl_3$): *δ* 8.11–7.99 (m, 6H), 7.62–7.55 (m, 2H), 7.54–7.48 (m, 1H), 7.48–7.43 (m, 2H), 7.43–7.37 (m, 2H), 7.34–7.27 (m, 2H), 5.60 (d, *J* = 5.1 Hz, 1H), 5.53 (d, *J* = 1.5 Hz, 1H), 5.19 (s, 1H), 4.85 (dd, *J* = 12.0, 3.5 Hz, 1H), 4.70 (dd, *J* = 12.0, 4.8 Hz, 1H), 4.58 (dd, *J* = 4.9, 3.4 Hz, 1H), 3.50 (s, 3H). ^{13}C NMR (101 MHz, $CDCl_3$): *δ* 166.3, 165.9, 165.6, 133.64, 133.62, 133.2, 130.1, 130.0, 129.9, 129.3, 129.2, 128.64, 128.60, 128.4, 107.0, 82.3, 81.0, 78.1, 77.5, 77.2, 76.8, 63.9, 55.1. HRMS (ESI): *m/z* = $[M+Na]^+$ calc for $C_{27}H_{24}O_8Na$ 499.1363, found 499.1363.

Ethyl 2,3,5,-tri-O-benzoyl-1-thio-α-L-arabinofuranoside (S2)

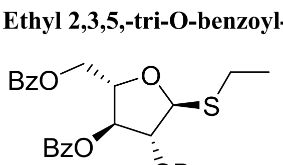

Methyl 2,3,5-tri-*O*-benzoyl-α-L-arabinofuranoside **S1** (20 g, 42 mmol) was dissolved in anhydrous CH_2Cl_2 (400 mL). To this, EtSH (3.75 mL, 50 mmol) and $BF_3{\cdot}Et_2O$ (15.7 mL, 125 mmol) were added and the mixture was stirred for 5 hrs at 0 °C. The reaction was diluted with CH_2Cl_2 (200 mL) and washed with saturated $NaHCO_3$ (1 x 300 mL), brine (1 x 300 mL), and then dried over $MgSO_4$, filtrated and concentrated. Flash purification by silica column chromatography (9:1 hexane-EtOAc) gave benzoylated thioarabinofuranoside **S2** as a colorless oil (17.2 g, 34 mmol, 81%). ^1H NMR (400 MHz, $CDCl_3$): *δ* 8.11 (dd, *J* = 8.1, 1.5 Hz, 2H), 8.04 (ddd, *J* = 12.7, 8.2, 1.5 Hz, 4H), 7.58 (dd, *J* = 8.3, 6.8 Hz, 2H), 7.54–7.44 (m, 3H), 7.40 (t, *J* = 7.7 Hz, 2H), 7.31 (t, *J* = 7.6 Hz, 2H), 5.64 (d, *J* = 4.5 Hz, 2H), 5.57 (t, *J* = 1.3 Hz, 1H), 4.88–4.68 (m, 3H), 2.91–2.64 (m, 2H), 1.36 (t, *J* = 7.4 Hz, 3H). ^{13}C NMR (101 MHz, $CDCl_3$): *δ* 166.3, 165.8, 165.6, 133.71, 133.66, 133.2, 130.1, 130.0, 129.9, 129.2, 129.1, 128.7, 128.4, 88.3, 83.2, 80.8, 63.6, 25.5, 15.0. HRMS (ESI): *m/z* = $[M+Na]^+$ calc for $C_{28}H_{26}O_7SNa$ 529.1291, found 529.1293.

Ethyl 1-thio-α-L-arabinofuranoside (6)

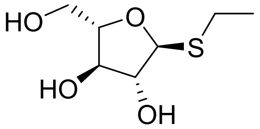

Ethyl 2,3,5-tri-*O*-benzoyl-1-thio-α-L-arabinofuranoside **S2** (50 g, 98.7 mmol) was dissolved in methanol (330 mL) and NaOMe (1.1 g, 19.7 mmol) was added. The reaction mixture was stirred overnight, and neutralized with 1N $KHSO_4$. The crude mixture was concentrated and flash chromatography (using a gradient from 0 to 10% MeOH in CH_2Cl_2) afforded thioarabinofuranoside **6** as a white amorphous solid (18.5 g, 95.2 mmol, 96%). ^1H NMR (400 MHz, D_2O): δ 5.15 (d, J = 5.3 Hz, 1H), 4.10–3.98 (m, 3H), 3.83 (dd, J = 12.5, 2.6 Hz, 1H), 3.74 (dd, J = 12.4, 4.8 Hz, 1H), 2.81–2.66 (m, 2H), 1.30 (t, J = 7.5 Hz, 3H). ^{13}C NMR (101 MHz, D_2O): δ 90.3, 84.6, 83.3, 78.1, 63.1, 27.3, 16.9. HRMS (ESI): *m/z* = $[M+Na]^+$ calc for $C_7H_{14}O_4SNa$ 217.0505, found 217.0507.

Ethyl 3,5-O-TIPS-1-thio-α-L-arabinofuranoside (S3)

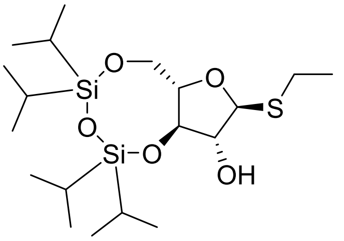

Ethyl 1-thio-α-L-arabinofuranoside **6** (5.1 g, 26.4 mmol) was dissolved in pyridine (75 mL) and $TIPDSiCl_2$ (9.7 mL, 30.6 mmol) was added. After stirring for 3 hrs, the reaction mixture was concentrated and co-evaporated with toluene until most traces of pyridine were removed. The residue was taken up in EtOAc and washed once with H_2O. The organic phase was dried over $MgSO_4$, filtrated and concentrated. The product was purified by flash chromatography (eluent was a gradient of 10 to 20% EtOAc in hexane) to afford the title product **S3** as a colorless oil (9.7 g, 22.2 mmol, 84%). ^1H NMR (400 MHz, $CDCl_3$): δ 5.08 (d, J = 5.6 Hz, 1H), 4.20 (dd, J = 8.5, 6.6 Hz, 1H), 4.09 (ddd, J = 7.2, 5.4, 2.7 Hz, 1H), 3.98–3.94 (m, 2H), 3.89 (dt, J = 8.5, 3.2 Hz, 1H), 2.78–2.58 (m, 2H), 2.27 (d, J = 3.8 Hz, 1H), 1.29 (t, J = 7.5 Hz, 3H), 1.13–0.99 (m, 28H). ^{13}C NMR (101 MHz, $CDCl_3$): δ 88.2, 82.3, 80.2, 76.5, 61.3, 25.7, 17.6, 17.4, 17.24, 17.20, 17.1, 15.2, 13.7, 13.3, 12.9, 12.7. HRMS (ESI): *m/z* = $[M+Na]^+$ calc for $C_{19}H_{40}O_5SSi_2Na$ 459.2027, found 459.2015.

Ethyl 2-O-PMB-3,5-O-TIPS-1-thio-α-L-arabinofuranoside (7)

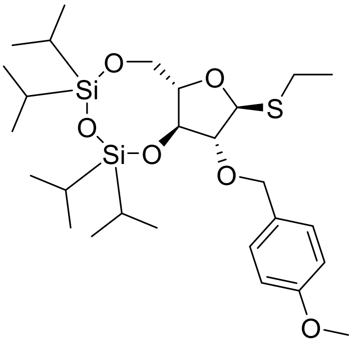

Ethyl 3,5-*O*-TIPS-1-thio-α-L-arabinofuranoside **S3** (18 g, 41.2 mmol) was dissolved in anhydrous THF (343 mL) and cooled down to 0 °C. To this, NaH (4.9 g, 123.6 mmol) was added and the mixture was stirred for 30 min. Freshly made PMBBr **S5** (*vide infra*) (9.9 g, 49.4 mmol) was added and the reaction mixture was stirred overnight. Upon completion, the reaction was quenched with saturated NH_4Cl and the organic products were extracted from the mixture with EtOAc. The combined organic layers were washed with H_2O (2 x 200 mL) and brine (1 x 200 mL), after which it was dried over $MgSO_4$, filtrated and concentrated *in vacuo*. The crude product was purified by flash chromatography (5% Et_2O in hexane) to afford the title product **7** as a slightly yellow oil (12 g, 21.5 mmol, 52%). ^1H NMR (400 MHz, $CDCl_3$): δ 7.30–7.26 (m, 2H), 6.90–6.84 (m, 2H), 5.21 (d, J = 4.2 Hz, 1H), 4.65–4.50 (m, 2H), 4.27 (dd, J = 8.3, 5.8 Hz, 1H), 3.97 (dd, J = 4.9, 3.0 Hz, 2H), 3.93 (td, J = 5.2, 4.6, 2.8 Hz, 1H), 3.89 (dd, J = 5.9, 4.2 Hz, 1H), 3.81 (s, 3H), 2.79–2.55 (m, 2H), 1.29 (t, J = 7.4 Hz, 3H), 1.10–1.01 (m, 28H). ^{13}C NMR (101 MHz, $CDCl_3$): δ 159.4, 130.1, 129.6, 113.8, 89.3, 86.6, 79.9, 76.3, 72.5, 61.3, 55.4, 25.6, 17.6, 17.5, 17.25, 17.21, 17.1, 15.1, 13.7, 13.3, 13.0, 12.7. HRMS (ESI): *m/z* = $[M+Na]^+$ calc for $C_{27}H_{48}O_6SSi_2Na$ 579.2602, found 579.2603.

Ethyl 2-*O*-PMB-3,5-*O*-benzyl-1-thio-α-L-arabinofuranoside (8) & Ethyl 2-*O*-PMB-3,5-*O*-benzyl-1-thio-β-L-arabinofuranoside (S4)

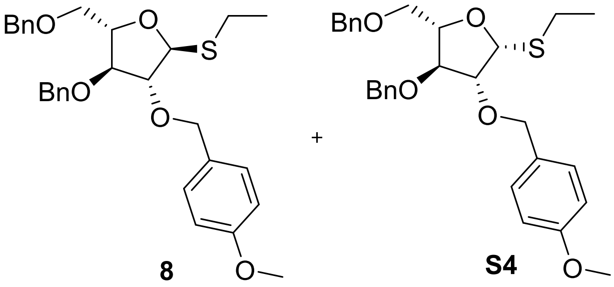

8 + S4

Ethyl 2-*O*-PMB-3,5-*O*-TIPS-1-thio-α-L-arabinofuranoside **7** (6.9 g, 12.3 mmol) was dried by means of co-evaporation with toluene (3 x 20 mL). Compound **7** was dissolved in anhydrous THF (98 mL) and tetra-*n*-butylammonium fluoride (24.7 mL, 1M in THF) was added. The reaction mixture was stirred for 15 min at room temperature after which it was concentrated, redissolved in anhydrous THF (123 mL) and cooled down to 0 °C. To this homogeneous solution, NaH (3.0

g, 74 mmol) was added and the mixture was allowed to stir for a couple of minutes at 0 ^{0}C. Then, benzylbromide (7.3 mL, 61.7 mmol) was added and the reaction mixture stirred overnight at room temperature. The completed reaction was quenched with MeOH and a couple droplets of 1M HCl before it was concentrated *in vacuo*. The crude product was purified by flash chromatography (gradual 0 to 10% EtOAc in hexane) resulting in a mixture of α- and β-arabinofuranosides **8** & **S4** in a ratio of 3.25:1 (5.3 g, 10.7 mmol, 87%). Analysis of α-L-arabinofuranoside **8**: ^{1}H NMR (400 MHz, $CDCl_3$): δ 7.28–7.17 (m, 12H), 6.88–6.78 (m, 2H), 5.31 (d, J = 2.4 Hz, 1H), 4.54–4.33 (m, 6H), 4.25 (dq, J = 5.1, 3.7 Hz, 1H), 3.92 (tt, J = 4.4, 2.4 Hz, 2H), 3.75 (s, 3H), 3.60 (qd, J = 10.9, 4.4 Hz, 2H), 2.72–2.55 (m, 2H), 1.26 (t, J = 7.4 Hz, 3H). ^{13}C NMR (101 MHz, $CDCl_3$): δ 159.5, 138.3, 137.9, 129.8, 129.6, 128.5, 128.4, 127.93, 127.86, 127.7, 114.0, 88.5, 87.4, 83.8, 80.0, 73.5, 72.3, 71.8, 69.3, 55.4, 25.4, 15.0. HRMS (ESI): *m/z* = $[M+Na]^+$ calc for $C_{29}H_{34}O_5SNa$ 517.2019, found 517.2024. Analysis of β-L-arabinofuranoside **S4**: ^{1}H NMR (400 MHz, $CDCl_3$): δ 7.27–7.19 (m, 12H), 6.84–6.79 (m, 2H), 5.35 (d, J = 5.0 Hz, 1H), 4.52–4.36 (m, 6H), 4.13–4.05 (m, 2H), 3.97 (t, J = 4.0 Hz, 1H), 3.74 (s, 3H), 3.67 (dd, J = 9.9, 6.4 Hz, 1H), 3.59 (dd, J = 9.9, 6.5 Hz, 1H), 2.63 (qd, J = 7.4, 2.8 Hz, 2H), 1.24 (t, J = 7.4 Hz, 3H). ^{13}C NMR (101 MHz, $CDCl_3$): δ 159.5, 138.4, 138.0, 129.8, 129.7, 128.54, 128.49, 127.92, 127.89, 127.86, 127.7, 114.0, 87.0, 84.1, 83.9, 82.2, 73.5, 72.2, 72.0, 71.6, 55.4, 25.0, 15.2. HRMS (ESI): *m/z* = $[M+Na]^+$ calc for $C_{29}H_{34}O_5SNa$ 517.2019, found 517.2020.

Fmoc-Hyp-OBn (10)

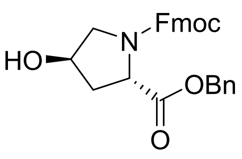

Fmoc-Hyp(*t*Bu)-OH (5.0 g, 12.2 mmol) was dissolved in anhydrous CH_2Cl_2 (33 mL) and cooled down to 0 °C. To this, benzyl alcohol (1.4 mL, 13.4 mmol), DMAP (150 mg, 1.22 mmol) and EDC•HCl (2.6 g, 13.4 mmol) were added and the reaction mixture stirred for 2 hrs on ice. The mixture was washed with 20% aqueous citrate and subsequently concentrated *in vacuo*. The residue was treated with 95% TFA (20 mL) for 10 min. The mixture was partitioned between CH_2Cl_2 and aqueous $NaHCO_3$, separated and the organic layer was concentrated. Flash chromatography (1:1 EtOAc:hexane) resulted in the title compound **10** as a colorless oil (4.2 g, 9.4 mmol, 77%). The NMR spectra are reported for a mixture of two rotamers caused by the tertiary carbamate functionality of Fmoc-protected Hyp: ^{1}H NMR (400 MHz, $CDCl_3$): δ 7.80–7.70 (m, 2H), 7.62–7.49 (m, 2H), 7.43–7.21 (m, 9H), 5.26–5.01 (m, 2H), 4.62–4.21 (m, 4.5H), 3.98 (t, J = 6.9 Hz, 0.5H), 3.77–3.63 (m, 1.5H), 3.55 (dt, J = 11.4, 2.1 Hz, 0.5H), 2.45–2.24 (m, 1H), 2.15–2.00 (m, 2H). ^{13}C NMR (101 MHz, $CDCl_3$): δ 172.4, 172.3, 154.8, 154.7, 144.3, 144.2, 144.0, 143.7, 141.5, 141.3, 135.8, 135.2, 128.7, 128.6, 128.4, 128.3, 127.8, 127.2, 125.3, 125.1, 120.1, 70.3, 69.5, 67.9, 67.7, 67.14, 67.07, 58.2, 57.9, 55.4, 54.8, 47.4, 47.3, 39.5, 38.5. HRMS (ESI): *m/z* = $[M+Na]^+$ calc for $C_{27}H_{25}NO_5Na$ 466.1625, found 466.1626.

1-(bromomethyl)-4-methoxybenzene (S5)

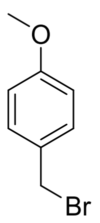

p-Methoxybenzylalcohol (11.5 mL, 92 mmol) was dissolved in Et_2O (100 mL) and cooled down to 0 °C. To this, PBr_3 (4.3 mL, 46 mmol) was added dropwise and the reaction was stirred for 3 hrs before the mixture was poured on a mixture of saturated $NaHCO_3$ and ice. The organic layer was dried over $MgSO_4$, filtered and concentrated *in vacuo* to give title compound **S5** (18.5 g, 92 mmol, quant.) as a clear oil that was used without further purification. ^{1}H NMR (400 MHz, $CDCl_3$): δ 7.38–7.27 (m, 2H), 6.93–6.79 (m, 2H), 4.51 (s, 2H), 3.81 (s, 3H). ^{13}C NMR (101 MHz, $CDCl_3$): δ 159.8, 130.6, 130.1, 114.3, 55.4, 34.1.

Synthesis of mono-, di-, and triarabinosylated hydroxyproline (14-16)

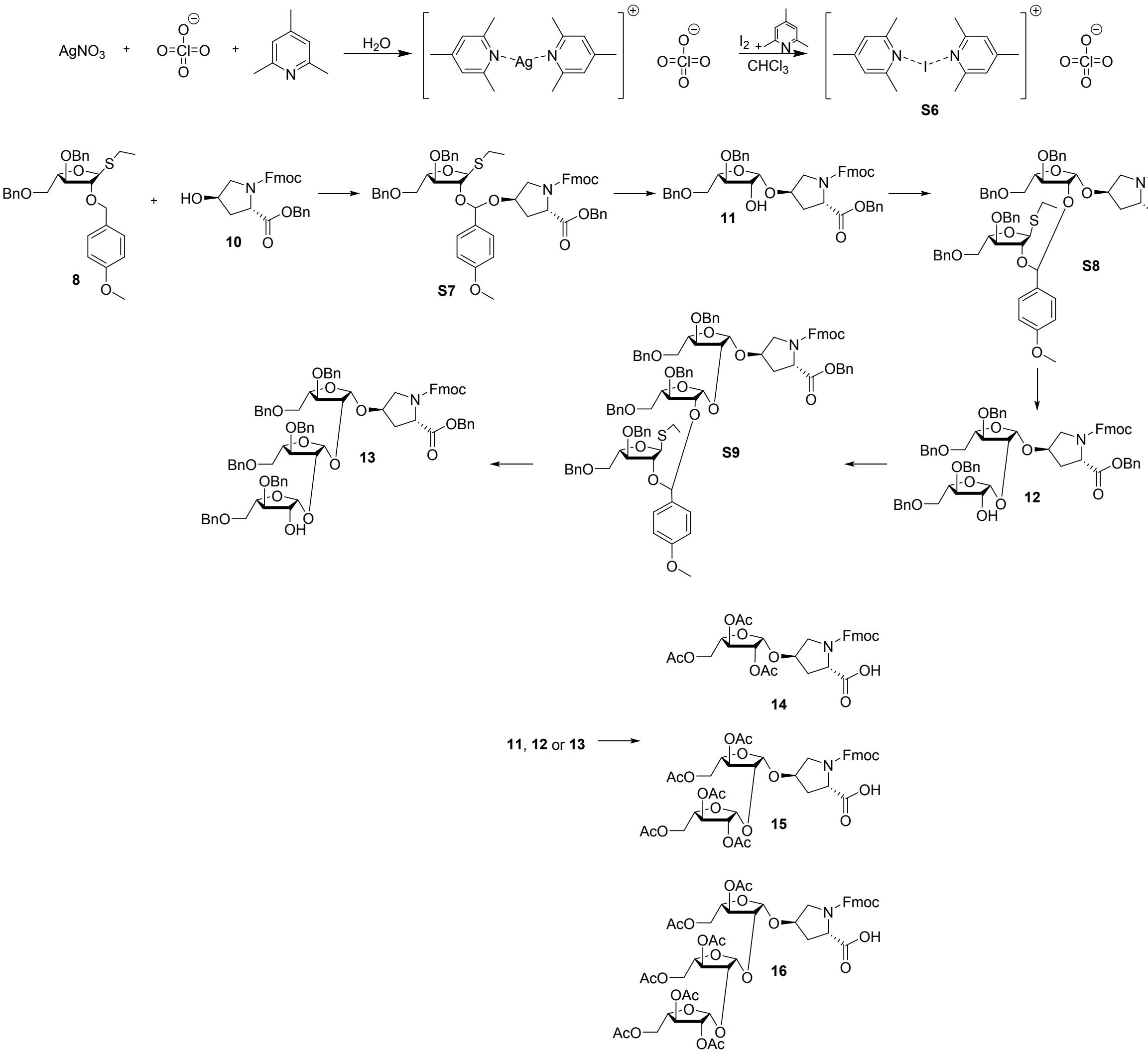

Iodonium di-collidine perchlorate (IDCP) (S6)

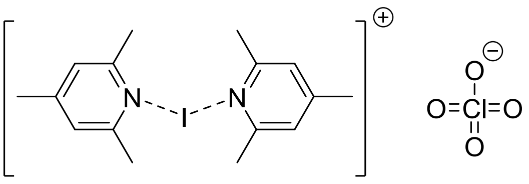

Silver di-*sym*-collidine perchlorate was prepared by adding *sym*-collidine (20 mL) to a solution of $AgNO_3$ (9 g) and $NaClO_4$ (11 g) in H_2O (100 mL) under vigorous stirring. The resulting white curdy precipitate was filtered and washed repeatedly with H_2O, ethanol and ether and finally dried under vacuum over P_2O_5. Then, silver di-*sym*-collidine was suspended in chloroform (150 mL) and *sym*-collidine (1 mL). To this, iodine (7.6 g, 30 mmol) was added and the mixture was allowed to stir a few minutes. The mixture was filtered through Celite and the filtrate was crystallized with the use of ether. The crystals were dried under high vacuum to give the title compound as a fine light yellow powder in quantitative yield. ^1H NMR (400 MHz, D_2O): δ 7.32 (s, 4H), 2.59 (s, 12H), 2.46 (s, 6H).

Fmoc-[(3,5-Bn)Ara$_1$]-2-PMB acetal Hyp-OBn (S7)

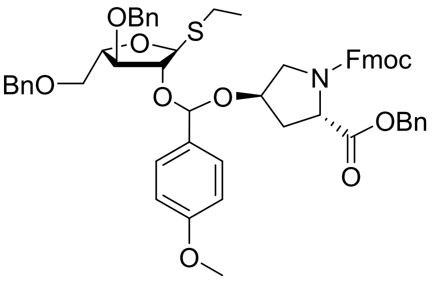

Arabinosedonor **8** (175 mg, 0.35 mmol) and hydroxyproline acceptor **10** (105 mg, 0.24 mmol) were dissolved in anhydrous CH_2Cl_2 (9.5 mL). 4 Å molecular sieves and 2,3-dichloro-5,6-dicyanobenzoquinone (DDQ) (107 mg, 0.47 mmol) were added. The mixture was stirred for 1 hr at room temperature and quenched with an aqueous solution of ascorbic acid (0.7%), citric acid (1.3%) and NaOH (0.9%). The organic phase was washed with aqueous $NaHCO_3$ and concentrated *in vacuo*. The crude product was purified by silica gel column chromatography (20% EtOAc in hexane) to afford the title compound **S7** as a slight yellow oil (133 mg, 0.14 mmol, 60%). The NMR reported are for a mixture of *cis/trans* rotamers originating from the tertiary carbamate moiety of Fmoc-protected Hyp: ^{1}H NMR (400 MHz, $CDCl_3$): δ 7.81–7.70 (m, 2H), 7.62–7.47 (m, 2H), 7.43–7.14 (m, 20H), 6.93–6.81 (m, 2H), 5.62 (d, J = 6.2 Hz, 0.4H), 5.42 (d, J = 8.7 Hz, 0.6H), 5.33 (dd, J = 8.8, 2.6 Hz, 0.6H), 5.26–5.01 (m, 2H), 4.61–4.46 (m, 3H), 4.43–4.32 (m, 3H), 4.32–4.17 (m, 3H), 4.07 (dt, J = 16.2, 2.8 Hz, 0.7H), 4.02–3.91 (m, 1.4H), 3.83–3.74 (m, 3H), 3.71–3.54 (m, 3.4H), 2.75–2.51 (m, 2H), 2.47–2.25 (m, 1H), 2.13–1.95 (m, 1.5H) 0.96–0.79 (m, 3H). ^{13}C NMR (101 MHz, $CDCl_3$): δ 172.4, 160.4, 154.8, 144.3, 143.9, 143.8, 141.4, 138.2, 137.8, 128.7, 128.50, 128.48, 128.4, 128.3, 128.2, 127.9, 127.8, 127.2, 125.33, 125.26, 125.1, 120.1, 114.0, 102.0, 101.9, 88.1, 86.4, 86.3, 84.1, 83.9, 80.3, 73.7, 73.51, 73.46, 72.8, 72.3, 72.2, 69.3, 67.8, 67.1, 67.0, 58.3, 58.0, 55.43, 55.40, 52.0, 47.2, 29.8, 25.4, 15.0. HRMS (ESI): m/z = [M+Na]$^+$ calc for $C_{56}H_{57}NO_{10}SNa$ 958.3595, found 958.3596.

Fmoc-[(3,5-Bn)Ara$_1$]Hyp-OBn (11)

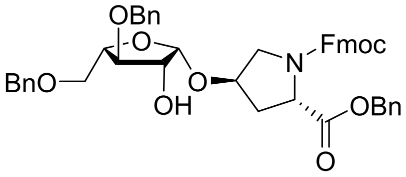

Mixed acetal intermediate **S7** (2.2 g, 2.4 mmol) was dissolved in anhydrous CH_2Cl_2 (69 mL). 4 Å molecular sieves and IDCP (3.3 g, 7.0 mmol) were added and the reaction mixture was allowed to stir for 30 min. Upon completion of the reaction, the mixture was washed with saturated $Na_2S_2O_3$ and 20% sodium citrate. The organic phase was dried over $MgSO_4$, filtered, and concentrated. The crude product was purified by silica gel column chromatography (1:2 EtOAc:hexane) to afford the title product **11** as a slight yellow oil (1.3 g, 1.8 mmol, 74%). The NMR reported are for a mixture of *cis/trans* rotamers originating from the tertiary carbamate moiety of Fmoc-protected Hyp: ^{1}H NMR (400 MHz, $CDCl_3$) δ 7.76 (dd, J = 10.7, 7.5 Hz, 2H), 7.63–7.47 (m, 2H), 7.45–7.15 (m, 16H), 5.26–5.07 (m, 2H), 5.04 (dd, J = 8.5, 3.7 Hz, 0.8H), 5.00 (d, J = 4.8 Hz, 0.4H), 4.77 (d, J = 11.9 Hz, 1H), 4.61 (dd, J = 12.1, 2.7 Hz, 1H), 4.55–4.32 (m, 5H), 4.31–4.20 (m, 2H), 4.14–4.07 (m, 1H), 3.99 (t, J = 7.0 Hz, 0.5H), 3.92–3.83 (m, 1H), 3.68 (dd, J = 7.2, 4.2 Hz, 1H), 3.60–3.42 (m, 2H), 2.51–2.34 (m, 1H), 2.15–1.99 (m, 1H). ^{13}C NMR (101 MHz, $CDCl_3$) δ 172.2, 154.9, 154.5, 144.5, 144.1, 143.9, 141.4, 138.0, 135.6, 128.7, 128.59, 128.55, 128.5, 128.3, 128.0, 127.93, 127.88, 127.85, 127.78, 127.23, 127.15, 125.3, 125.2, 125.1, 120.13, 120.06, 101.3, 101.2, 83.9, 83.8, 80.9, 80.8, 77.8, 77.6, 76.3, 75.5, 73.5, 72.2, 72.1, 71.4, 67.8, 67.7, 67.2, 67.1, 58.3, 58.0, 52.3, 51.8, 47.3, 37.8, 36.6. HRMS (ESI): m/z = [M+Na]$^+$ calc for $C_{46}H_{45}NO_9Na$ 778.2986, found 778.2990.

Fmoc-[(3,5-Bn)Ara$_2$]-2-PMB acetal Hyp-OBn (S8)

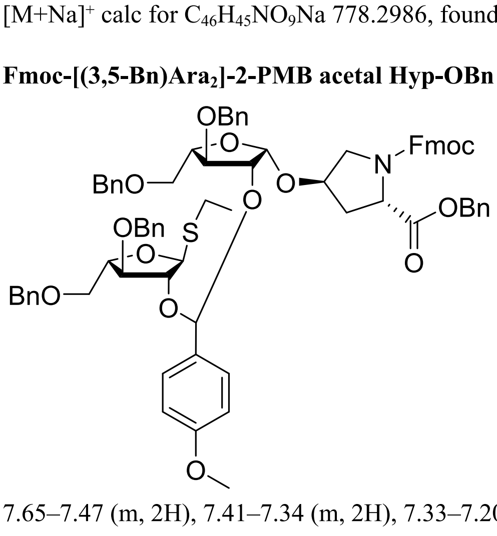

Arabinosedonor **8** (922 mg, 1.9 mmol) and Ara$_1$Hyp **11** (470 mg, 0.6 mmol) were dissolved in anhydrous CH_2Cl_2 (28 mL). 4 Å molecular sieves and DDQ (565 mg, 2.5 mmol) were added. The mixture was allowed to stir for 1 hour at room temperature before quenching with an aqueous solution of ascorbic acid (0.7%), citric acid (1.3%) and NaOH (0.9%). The organic phase was washed with aqueous $NaHCO_3$ and concentrated *in vacuo*. The crude product was purified by silica gel column chromatography (25% EtOAc in hexane) to afford the title compound **S8** as a slight yellow oil (333 mg, 0.3 mmol, 43%). The NMR reported is for a mixture of *cis/trans* rotamers originating from the tertiary carbamate moiety of Fmoc-protected Hyp: ^{1}H NMR (400 MHz, $CDCl_3$): δ 7.80 – 7.70 (m, 2H), 7.65–7.47 (m, 2H), 7.41–7.34 (m, 2H), 7.33–7.20 (m, 27H), 7.17–7.06 (m, 2H), 6.87–6.73 (m, 2H), 5.63 (d, J = 8.2 Hz, 0.6H), 5.43 (d, J = 12.4 Hz, 0.5H), 5.37 (d, J = 2.6 Hz, 0.3H), 5.34–5.26 (m, 1H), 5.24–4.99 (m, 2H), 4.91 (d, J = 4.4 Hz, 0.5H), 4.78–4.60 (m, 1.5H), 4.56–4.39 (m, 8H), 4.39–4.16 (m, 6H), 4.16–3.89 (m, 5H), 3.75 (s, 2H), 3.66 (d, J = 8.0 Hz, 2H), 3.63–3.53 (m, 2H), 3.47 (dd, J = 7.7, 5.7 Hz, 1H), 3.43–3.35 (m, 1H), 2.75–2.51 (m, 2H), 2.51–2.30 (m, 1.3H),

2.10–1.97 (m, 1.5H), 1.24 (dtd, J = 15.4, 7.3, 3.8 Hz, 3H). ^{13}C NMR (101 MHz, $CDCl_3$): δ 172.2, 164.8, 155.0, 154.4, 144.1, 143.71, 143.66, 141.4, 137.8, 137.1, 132.1, 128.72, 128.70, 128.60, 128.55, 128.5, 128.3, 128.2, 128.01, 127.99, 127.93, 127.88, 127.2, 125.2, 125.1, 120.13, 120.07, 114.5, 101.3, 101.2, 92.0, 85.4, 83.9, 83.1, 80.9, 79.5, 76.4, 75.5, 73.9, 73.5, 72.22, 72.16, 71.4, 69.8, 67.7, 67.2, 67.1, 58.3, 58.0, 55.7, 52.3, 47.3, 26.1, 15.2. HRMS (ESI): *m/z* = [M+Na]$^+$ calc for $C_{75}H_{77}NO_{14}SNa$ 1270.4963, found 1270.4958.

Fmoc-[(3,5-Bn)Ara$_2$]Hyp-OBn (12)

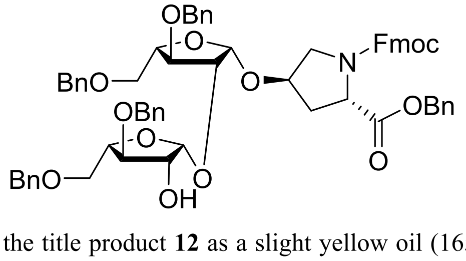

Intermediate **S8** (309 mg, 0.25 mmol) was dissolved in anhydrous CH_2Cl_2 (20 mL). 4 Å molecular sieves and IDCP (348 mg, 0.74 mmol) were added and the reaction mixture was allowed to stir for 1 hr. Upon reaction completion, the mixture was washed with saturated $Na_2S_2O_3$ and 20% sodium citrate. The organic phase was dried over $MgSO_4$, filtered, and concentrated. The crude product was purified by silica gel chromatography (gradual from 20% to 40% EtOAc in hexane) to afford the title product **12** as a slight yellow oil (165 mg, 0.15 mmol, 63%). The NMR reported is for a mixture of *cis/trans* rotamers originating from the tertiary carbamate moiety of Fmoc-protected Hyp: ^{1}H NMR (400 MHz, $CDCl_3$): δ 7.81–7.68 (m, 2H), 7.65–7.53 (m, 2H), 7.43–7.13 (m, 29H), 5.16 (dd, J = 29.3, 16.8 Hz, 1.7H), 5.07 (d, J = 3.8 Hz, 0.7H), 5.01 (d, J = 4.4 Hz, 0.5H), 4.91 (dd, J = 9.6, 4.8 Hz, 1H), 4.74 (d, J = 12.0 Hz, 0.5H), 4.67 (dd, J = 11.7, 6.5 Hz, 1H), 4.63–4.56 (m, 1H), 4.56–4.47 (m, 4H), 4.47–4.38 (m, 4H), 4.38–4.23 (m, 3H), 4.22–4.00 (m, 5H), 3.89–3.73 (m, 1.5H), 3.72–3.58 (m, 1.5H), 3.55 (dd, J = 9.5, 5.7 Hz, 2H), 3.48 (t, J = 5.9 Hz, 2H), 3.00–2.81 (m, 1H), 2.44–2.28 (m, 1H). ^{13}C NMR (101 MHz, $CDCl_3$): δ 172.5, 172.3, 155.0, 154.8, 144.2, 144.14, 144.06, 144.0, 141.5, 141.4, 141.3, 138.0, 137.8, 135.7, 135.4, 128.7, 128.54, 128.51, 128.48, 128.44, 128.38, 128.3, 128.03, 127.97, 127.93, 127.90, 127.86, 127.80, 127.76, 127.71, 127.66, 127.2, 125.4, 125.3, 125.24, 125.18, 120.1, 120.0, 102.5, 102.2, 99.3, 98.7, 84.1, 83.9, 82.1, 81.9, 81.8, 81.7, 81.12, 81.06, 80.4, 77.8, 75.6, 74.0, 73.4, 73.3, 73.2, 72.5, 72.3, 72.2, 72.1, 71.9, 71.8, 71.5, 71.4, 68.0, 67.8, 67.15, 67.10, 58.2, 58.0, 51.6, 51.3, 47.3, 37.7, 36.6. HRMS (ESI): *m/z* = [M+Na]$^+$ calc for $C_{65}H_{65}NO_{13}Na$ 1090.4348, found 1090.4348.

Fmoc-[(3,5-Bn)Ara$_3$]-2-PMB acetal Hyp-OBn (S9)

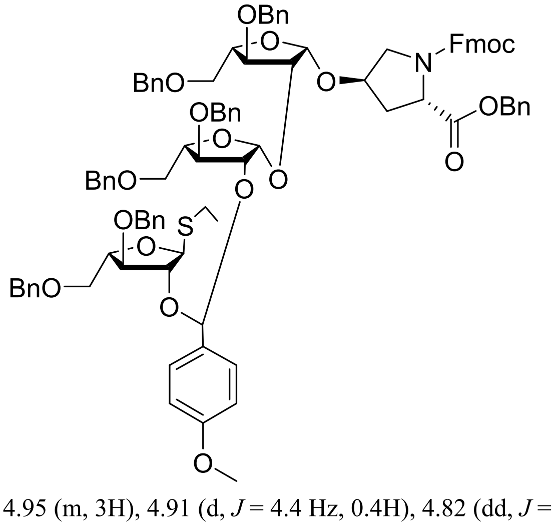

Arabinosedonor **8** (529 mg, 1.00 mmol) and Ara$_2$Hyp **12** (381 mg, 0.36 mmol) were dissolved in anhydrous CH_2Cl_2 (16 mL). 4 Å molecular sieves and DDQ (243 mg, 1.0 mmol) were added. The mixture was allowed to stir for 3 hours at room temperature before quenching with an aqueous solution of ascorbic acid (0.7%), citric acid (1.3%) and NaOH (0.9%). The organic phase was washed with aqueous $NaHCO_3$ and concentrated *in vacuo*. The crude product was purified by silica gel column chromatography (25% EtOAc in hexane) to afford the title compound **S8** as a slight yellow oil (295 mg, 0.19 mmol, 53%). The NMR reported is for a mixture of *cis/trans* rotamers originating from the tertiary carbamate moiety of Fmoc-protected Hyp: ^{1}H NMR (400 MHz, $CDCl_3$): δ 7.76 (d, J = 8.6 Hz, 1H), 7.71–7.58 (m, 2H), 7.55–7.37 (m, 2H), 7.35–7.02 (m, 38H), 6.92 (d, J = 8.4 Hz, 1H), 5.46 (d, J = 3.6 Hz, 0.3H), 5.23–4.95 (m, 3H), 4.91 (d, J = 4.4 Hz, 0.4H), 4.82 (dd, J = 8.7, 4.8 Hz, 0.7H), 4.70–4.16 (m, 18H), 4.15–3.86 (m, 6H), 3.81 (s, 2H), 3.77–3.65 (m, 2H), 3.64–3.42 (m, 6H), 3.42–3.34 (m, 2H), 2.80 (dd, J = 28.5, 8.8 Hz, 0.67H), 2.69–2.38 (m, 2H), 2.34–2.19 (m, 1H), 2.03–1.87 (m, 1H), 1.27–1.19 (m, 3H). ^{13}C NMR (101 MHz, $CDCl_3$): δ 172.4, 172.2, 164.7, 160.4, 155.0, 154.7, 144.2, 144.12, 144.08, 144.0, 141.4, 137.8, 132.1, 128.71, 128.68, 128.65, 128.59, 128.54, 128.51, 128.48, 128.44, 128.37, 128.3, 128.2, 128.03, 128.00, 127.97, 127.93, 127.89, 127.84, 127.80, 127.71, 127.65, 127.2, 125.4, 125.25, 125.19, 120.1, 120.0, 114.4, 102.5, 102.2, 99.3, 98.7, 92.0, 87.8, 85.4, 84.1, 83.9, 83.1, 82.1, 81.9, 81.8, 81.7, 81.1, 80.5, 79.5, 77.8, 75.6, 74.0, 73.9, 73.4, 73.3, 73.2, 72.5, 72.3, 72.2, 72.1, 71.9, 71.8, 71.5, 71.4, 69.8, 68.0, 67.9, 67.1, 58.2, 58.0, 55.7, 51.6, 51.3, 47.3, 37.7, 36.6, 29.8, 26.1, 15.2. HRMS (ESI): *m/z* = [M+Na]$^+$ calc for $C_{94}H_{97}NO_{18}SNa$ 1582.6318, found 1582.6320.

Fmoc-[(3,5-Bn)Ara$_3$]Hyp-OBn (13)

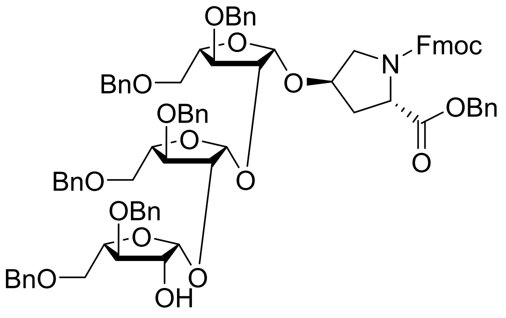

Intermediate **S9** (275 mg, 0.18 mmol) was dissolved in anhydrous CH_2Cl_2 (3 mL). 4 Å molecular sieves and IDCP (248 mg, 0.53 mmol) were added and the reaction mixture was allowed to stir for 1 hour. Upon reaction completion, the mixture was washed with saturated $Na_2S_2O_3$ and 20% sodium citrate. The organic phase was dried over $MgSO_4$, filtered, and concentrated. The crude product was purified by silica gel chromatography (gradual from 20% to 40% EtOAc in hexane) to afford the title product **12** as a slight yellow oil (85 mg, 0.06 mmol, 35%). The NMR reported is for a mixture of *cis/trans* rotamers originating from the tertiary carbamate moiety of Fmoc-protected Hyp: ^{1}H NMR (400 MHz, $CDCl_3$): δ 7.78–7.67 (m, 2H), 7.60–7.48 (m, 2H), 7.40–7.11 (m, 39H), 5.47 (d, J = 3.0 Hz, 0.5H), 5.27 (s, 0.2H), 5.23–4.94 (m, 5H), 4.72 (dd, J = 12.1, 5.5 Hz, 1H), 4.60–4.27 (m, 16H), 4.25–4.18 (m, 2H), 4.14–3.98 (m, 6H), 3.89 (q, J = 6.1 Hz, 1H), 3.69–3.60 (m, 1.5H), 3.59–3.39 (m, 6H), 3.00 (d, J = 8.3 Hz, 0.5H), 2.87 (d, J = 8.5 Hz, 0.5H), 2.42–2.22 (m, 1H). ^{13}C NMR (101 MHz, $CDCl_3$): δ 172.32, 172.28, 171.2, 154.8, 154.5, 144.2, 144.10, 144.07, 143.8, 141.4, 141.35, 141.29, 138.29, 138.27, 138.1, 138.05, 137.98, 137.9, 135.7, 135.4, 128.7, 128.6, 128.53, 128.47, 128.43, 128.39, 128.36, 128.2, 128.0, 127.93, 127.91, 127.87, 127.84, 127.82, 127.78, 127.76, 127.7, 127.6, 127.25, 127.21, 125.3, 125.20, 125.17, 120.04, 119.98, 101.5, 99.3, 98.9, 98.14, 98.07, 84.2, 84.1, 82.42, 82.37, 82.14, 82.08, 81.5, 80.9, 80.8, 80.7, 80.5, 80.4, 78.9, 78.6, 78.02, 77.99, 75.1, 74.3, 73.40, 73.36, 73.3, 73.24, 73.17, 72.5, 72.12, 72.10, 72.04, 71.96, 71.84, 71.80, 68.0, 67.9, 67.1, 60.5, 58.2, 57.9, 51.8, 51.2, 47.3, 47.2, 37.6, 36.5, 21.2, 14.3. HRMS (ESI): m/z = $[M+Na]^+$ calc for $C_{84}H_{85}NO_{17}Na$ 1402.5704, found 1402.5702.

Fmoc-[Ara(OAc)]$_1$Hyp-OBn (14)

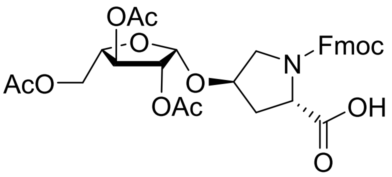

Compound **11** (103 mg, 0.13 mmol) was dissolved in CH_2Cl_2:MeOH (1:1, 3.4 mL) with 5 drops of acetic acid and hydrogenolyzed in the presence of 10% $Pd(OH)_2/C$ (50 mg) and H_2 at room temperature. After 16 hours of reaction time, the $Pd(OH)_2/C$ was refreshed and a new H_2 balloon was used. This was repeated after 25 hours and 45 hours. Reaction was completed after 64 hours, followed by filtration over Celite and concentration *in vacuo*. The deprotected compound was dissolved in H_2O:ACN (1:1, 2.9 mL) with 5% $NaHCO_3$ added until pH 8 is reached. The mixture is treated with Fmoc-OSu (89 mg, 0.26 mmol), some droplets of dioxane and a single droplet of acetone was added for solubility reasons and the mixture was stirred overnight. Upon reaction completion, the mixture was lyophilized and subsequently further treated with Ac_2O:pyridine (1:1, 3 mL) for 16 h at room temperature. The mixture was then quenched with H_2O and diluted with EtOAc. The organic layer was washed with 1 N $KHSO_4$, dried over $MgSO_4$, filtered and concentrated. The crude product was purified by silica gel column chromatography (gradual 0 to 10% MeOH in CH_2Cl_2 with 1% AcOH) and the obtained fractions were co-evaporated with cyclohexane to remove residual traces of acetic acid. This yielded in title product **14** (54 mg, 66%). The NMR reported is for a mixture of *cis/trans* rotamers originating from the tertiary carbamate moiety of Fmoc-protected Hyp: ^{1}H NMR (400 MHz, $CDCl_3$): δ 7.76 (dd, J = 7.6, 4.7 Hz, 2H), 7.58 (t, J = 8.2 Hz, 2H), 7.41–7.30 (m, 4H), 5.35 (d, J = 4.8 Hz, 1H), 5.33–5.27 (m, 1H), 4.95 (td, J = 5.9, 4.7, 2.5 Hz, 1H), 4.54–4.44 (m, 2H), 4.44–4.20 (m, 6H), 4.14–4.06 (m, 1H), 3.65–3.52 (m, 2H), 3.51–3.39 (m, 1H), 2.65–2.56 (m, 1H), 2.56–2.42 (m, 1H), 2.35–2.24 (m, 1H), 2.23–2.14 (m, 1H), 2.10 (t, J = 4.6 Hz, 6H), 2.04 (s, 2H), 1.97 (s, 1H). ^{13}C NMR (101 MHz, $CDCl_3$): δ 178.0, 177.5, 176.8, 170.9, 170.4, 155.7, 144.0, 143.8, 141.4, 127.9, 127.83, 127.77, 127.23, 127.18, 125.24, 125.16, 125.1, 124.9, 120.1, 99.9, 99.2, 79.0, 78.9, 77.2, 76.9, 75.8, 75.74, 75.67, 68.1, 67.9, 67.0, 65.5, 65.3, 58.1, 57.5, 52.1, 51.3, 47.3, 37.6, 36.3, 34.4, 20.91, 20.89, 20.87, 20.5, 20.4. HRMS (ESI): m/z = $[M+Na]^+$ calc for $C_{31}H_{33}NO_{12}Na$ 634.1895, found 634.1890.

Fmoc-[Ara(OAc)]$_2$Hyp-OBn (15)

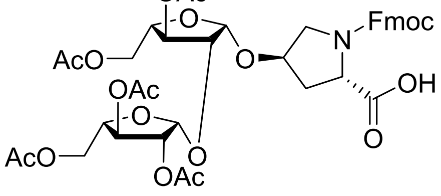

Compound **12** (128 mg, 0.12 mmol) was dissolved in CH_2Cl_2:MeOH (1:1, 3 mL) with 2 drops of acetic acid and hydrogenolyzed in the presence of 10% $Pd(OH)_2/C$ (50 mg) and H_2 at room temperature for 16 hours. Upon reaction completion, the mixture was filtered over Celite and concentrated *in vacuo*. Most Fmoc remained intact on the proline residue, however to ensure full conversion, the deprotected compound was treated with Fmoc-OSu (81 mg, 0.24 mmol) in H_2O:dioxane:acetone (1.2:1:0.2, 2.4 mL) with

5% $NaHCO_3$ added until pH 8 is reached. The mixture was allowed to stir overnight till completion and was then lyophilized. The product was further treated with Ac_2O:pyridine (1:1, 3 mL) for 16 h at room temperature. The mixture was then quenched with H_2O and diluted with EtOAc. The organic layer was washed with 1 N $KHSO_4$, dried over $MgSO_4$, filtered and concentrated. The crude product was purified by silica gel column chromatography (gradual 0 to 10% MeOH in CH_2Cl_2) to obtain title compound **15** (59 mg, 59%). The NMR reported are for a mixture of *cis/trans* rotamers originating from the tertiary carbamate moiety of Fmoc-protected Hyp: ^{1}H NMR (400 MHz, $CDCl_3$): δ 7.79–7.72 (m, 2H), 7.65–7.59 (m, 2H), 7.42–7.33 (m, 4H), 5.29 (s, 1H), 5.26 (d, J = 4.2 Hz, 1H), 5.14 (q, J = 5.9 Hz, 1H), 5.09 (dd, J = 8.2, 4.3 Hz, 1H), 4.95 (dd, J = 6.6, 4.5 Hz, 1H), 4.56 (ddd, J = 10.9, 8.3, 5.9 Hz, 1H), 4.49–4.12 (m, 10H), 4.09–4.02 (m, 1H), 3.86–3.60 (m, 2H), 3.58–3.42 (m, 1H), 2.63–2.51 (m, 1H), 2.50–2.40 (m, 1H), 2.34–2.25 (m, 1H), 2.11 (d, J = 3.3 Hz, 3H), 2.08 (s, 9H), 2.05 (d, J = 1.9 Hz, 3H). ^{13}C NMR (101 MHz, $CDCl_3$): δ 176.5, 170.84, 170.76, 170.7, 170.60, 170.56, 170.3, 170.2, 170.1, 169.3, 155.4, 154.9, 148.2, 144.2, 144.11, 144.07, 144.0, 141.4, 141.3, 137.6, 127.8, 127.7, 127.2, 127.1, 125.4, 125.3, 125.2, 120.1, 119.9, 99.4, 99.2, 98.8, 98.7, 79.6, 79.5, 79.4, 77.9, 77.6, 77.4, 77.0, 76.6, 76.5, 76.0, 75.9, 75.7, 74.7, 68.04, 67.97, 66.9, 65.89, 65.86, 65.9, 65.7, 53.5, 51.5, 51.1, 47.35, 47.27, 37.5, 36.1, 25.7, 20.94, 20.89, 20.85, 20.83, 20.78, 20.6, 17.7. HRMS (ESI): m/z = $[M+Na]^+$ calc for $C_{40}H_{45}NO_{18}Na$ 850.2529, found 850.2528.

Fmoc-[Ara(OAc)]$_3$Hyp-OBn (16)

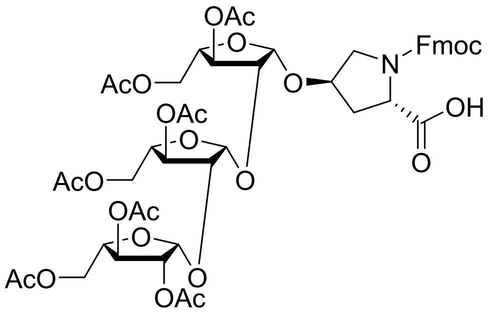

Compound **13** (85 mg, 0.06 mmol) was dissolved in CH_2Cl_2:MeOH (1:1, 2 mL) with 1 drop of acetic acid and hydrogenolyzed in the presence of 10% $Pd(OH)_2/C$ (50 mg) and H_2 at room temperature for 16 hours. Upon reaction completion, the mixture was filtered over Celite and concentrated *in vacuo*. The deprotected compound was treated with Fmoc-OSu (42 mg, 0.12 mmol) in H_2O:dioxane:acetone (1:0.8:0.2, 2 mL) with 5% $NaHCO_3$ added until pH 8 is reached. The mixture was allowed to stir for 3 hours until reaction completion and was then lyophilized. The product was further treated with Ac_2O:pyridine (1:1, 2 mL) for 16 h at room temperature. The mixture was then quenched with H_2O and diluted with EtOAc. The organic layer was washed with 1 N $KHSO_4$, dried over $MgSO_4$, filtered and concentrated. The crude product was purified by silica gel column chromatography (gradual 0 to 10% MeOH in CH_2Cl_2) to obtain title compound **16** (37 mg, 58%). The NMR reported is for a mixture of *cis/trans* rotamers originating from the tertiary carbamate moiety of Fmoc-protected Hyp: ^{1}H NMR (400 MHz, $CDCl_3$): δ 7.75 (d, J = 7.5 Hz, 2H), 7.58 (d, J = 7.5 Hz, 2H), 7.42–7.31 (m, 4H), 5.33 (d, J = 4.4 Hz, 1H), 5.27 (d, J = 4.3 Hz, 1H), 5.20–5.12 (m, 3H), 5.03–5.00 (m, 2H), 4.95 (dd, J = 7.2, 4.4 Hz, 1H), 4.70–4.63 (m, 1H), 4.55–4.47 (m, 3H), 4.44–4.36 (m, 6H), 4.33–4.25 (m, 4H), 4.13–4.05 (m, 6H), 3.70–3.61 (m, 2H), 2.69–2.53 (m, 1H), 2.42–2.35 (m, 1H), 2.09–2.06 (m, 13H), 1.96 (d, J = 5.1 Hz, 4H), 1.91 (s, 2H), 1.82 (s, 2H). ^{13}C NMR (101 MHz, $CDCl_3$): δ 171.0, 170.9, 170.8, 170.7, 170.6, 170.4, 170.3, 170.0, 169.9, 169.3, 165.7, 155.7, 154.7, 144.5, 144.0, 143.9, 141.4, 129.5, 128.0, 127.9, 127.8, 127.7, 127.3, 127.2, 125.5, 125.2, 125.1, 124.5, 120.1, 120.0, 98.8, 98.6, 97.84, 97.78, 97.5, 80.5, 80.4, 79.7, 79.6, 79.2, 79.0, 77.7, 77.4, 77.3, 76.6, 76.5, 75.6, 75.4, 75.0, 74.5, 68.1, 68.0, 66.3, 66.2, 65.6, 65.4, 51.8, 51.4, 47.3, 47.2, 37.6, 36.0, 29.8, 25.7, 20.94, 20.92, 20.86, 20.8, 20.7, 20.6, 20.5, 17.7. HRMS (ESI): m/z = $[M+Na]^+$ calc for $C_{49}H_{57}NO_{24}Na$ 1066.3163, found 1066.3169.

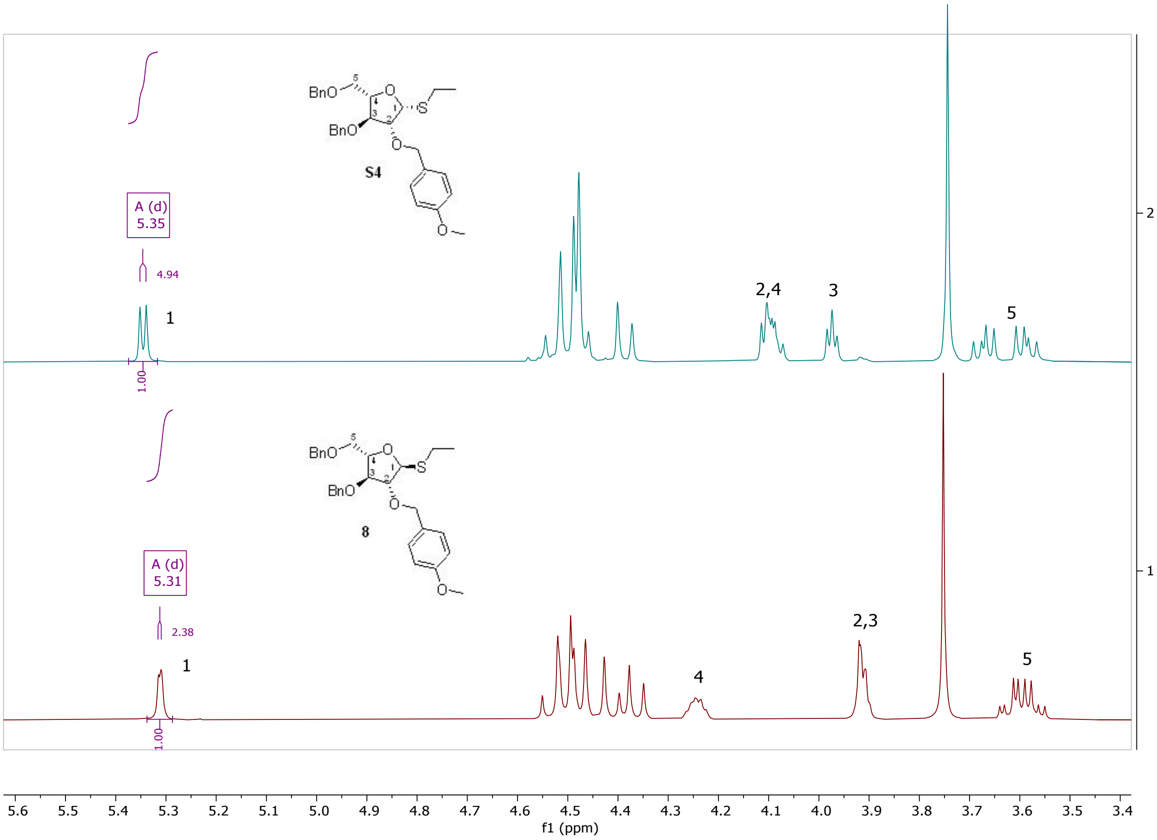

Figure S1. ^{1}H NMR spectra of furanose protons from anomers **8** & **S4**.

Synthesis and HPLC/MS data of *Brassica* PSY1 glycopeptides (1-4)

Fmoc-V-Hyp[Ara(OAc)]$_1$-Hyp(*t*Bu)-A-Wang resin (17)

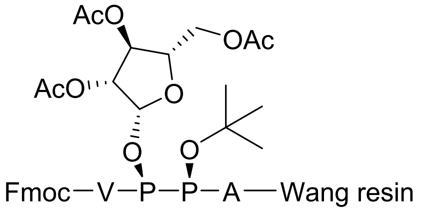

The first amino acids were coupled manually to Fmoc-Ala-Wang resin (72.2 mg, 0.05 mmol). Coupling of Fmoc-Hyp(*t*Bu)-OH was performed with an excess of 5 equivalents, as well as for HBTU/HOBt and 10 equivalents DIPEA. Fmoc-[(2,3,5-OAc)Ara]$_1$Hyp-OBn **14** (27.3 mg, 0.044 mmol) was coupled using a small deficiency compared to reactive groups on the resin to ensure most of tailor-made compound **14** was coupled. To monitor the coupling, a small amount of resin was cleaved with TFA cocktail and subsequently analyzed with the use of LC-MS (LXQ, 95% H_2O to 95% MeCN supplemented with 0.1% FA, gradient in 20 min). MS (ESI) *m/z* = $[M+H]^+$ calc for $C_{39}H_{46}N_3O_{15}$ 796.29, found 796.20; $[M+Na]^+$ calc for $C_{39}H_{45}N_3O_{15}Na$ 818.27, found 818.38. The reaction was also monitored by ninhydrin/chloranil tests. After completion of the coupling of **14** to the resin, remaining unreacted H_2N-Hyp(*t*Bu)-A-Wang resin was subsequently capped using an acetic anhydride mixture that contained a 50 fold molar excess of Ac_2O in the presence of DIPEA in DMF. Coupling of Fmoc-Val-OH was performed again with a 5 equivalent excess and was monitored in a similar way as previous coupling. MS (ESI) *m/z* = $[M+H]^+$ calc for $C_{44}H_{55}N_4O_{16}$ 895.36, found 895.22; $[M+Na]^+$ calc for $C_{44}H_{54}N_4O_{16}Na$ 917.34, found 917.45. Formed product on resin was further used for the synthesis of glycopeptide **21**.

Fmoc-V-Hyp[Ara(OAc)]$_2$-Hyp(*t*Bu)-A-Wang resin (18)

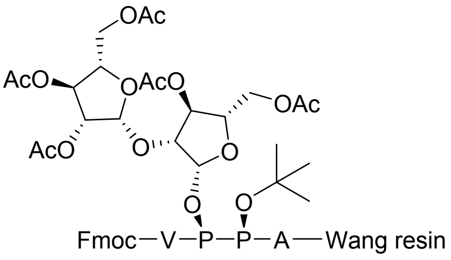

The first amino acids were coupled manually to, Fmoc-Ala-Wang resin (70.9 mg, 0.048 mmol). Coupling of Fmoc-Hyp(*t*Bu)-OH was performed with an excess of 5 equivalents, as well as for HBTU/HOBt and 10 equivalents DIPEA. Fmoc-[(2,3,5-OAc)Ara]$_2$Hyp-OBn **15** (36.3 mg, 0.044 mmol) was coupled using a small deficiency compared to reactive groups on the resin to ensure most of tailor-made compound **15** was coupled. To monitor the coupling, a small amount of resin was cleaved with TFA cocktail and subsequently analyzed with the use of LC-MS (LXQ, 95% H_2O to 95% MeCN supplemented with 0.1% FA, gradient in 20 min). HRMS (ESI) *m/z* = $[M+H]^+$ calc for $C_{48}H_{58}N_3O_{21}$ 1012.3558, found 1012.3580; $[M+Na]^+$ calc for $C_{48}H_{57}N_3O_{21}Na$ 1034.3377, found 1034.3403. The reaction was also monitored by ninhydrin/chloranil tests. After completion of the coupling of **14** to the resin, remaining unreacted H_2N-Hyp(*t*Bu)-A-Wang resin was subsequently capped using an acetic anhydride mixture that contained a 50 fold molar excess of Ac_2O in the presence of DIPEA in DMF. Coupling of Fmoc-Val-OH was performed again with a 5 equivalent excess and was monitored in a similar way as previous coupling. HRMS (ESI) *m/z* = $[M+H]^+$ calc for $C_{53}H_{67}N_4O_{22}$ 1111.4242, found 1111.4272; $[M+Na]^+$ calc for $C_{53}H_{66}N_4O_{22}Na$ 1133.4061, found 1133.4089. Formed product on resin was further used for the synthesis of glycopeptide **22**.

Fmoc-V-Hyp[Ara(OAc)]$_3$-Hyp(*t*Bu)-A-Wang resin (19)

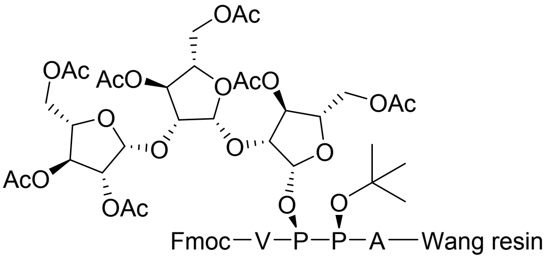

The first amino acids were coupled manually to, Fmoc-Ala-Wang resin (39.4 mg, 0.027 mmol). Coupling of Fmoc-Hyp(*t*Bu)-OH was performed with an excess of 5 equivalents, as well as for HBTU/HOBt and 10 equivalents DIPEA. Fmoc-[(2,3,5-OAc)Ara]$_3$Hyp-OBn **16** (25.4 mg, 0.024 mmol) was coupled using a small deficiency compared to reactive groups on the resin to ensure most of tailor-made compound **16** was coupled. To monitor the coupling, a small amount of resin was cleaved with TFA cocktail and subsequently analyzed with the use of LC-MS (LXQ, 95% H_2O to 95% MeCN supplemented with 0.1% FA, gradient in 20 min). HRMS (ESI) *m/z* = $[M+H]^+$ calc for $C_{57}H_{70}N_3O_{27}$ 1228.4191, found 1228.4196; $[M+Na]^+$ calc for $C_{57}H_{69}N_3O_{27}Na$ 1250.4010, found 1250.4014. The reaction was also monitored by ninhydrin/chloranil tests. After completion of the coupling of **14** to the resin, remaining unreacted H_2N-Hyp(*t*Bu)-A-Wang resin was subsequently capped using an acetic anhydride mixture that contained a 50 fold molar excess of Ac_2O in the presence of DIPEA in DMF. Coupling of Fmoc-Val-OH was performed again with a 5 equivalent excess and was monitored in a similar way as previous coupling. HRMS (ESI) *m/z* = $[M+H]^+$ calc for $C_{62}H_{79}N_4O_{28}$ 1327.4876, found 1327.4882; $[M+Na]^+$ calc for $C_{62}H_{78}N_4O_{28}Na$ 1349.4695, found 1349.4701. Formed product on resin was further used for the synthesis of glycopeptide **23**.

H-Asp-Tyr(SO$_2$ONp)-Gly-Asp-Pro-Ser-Ala-Asn-Pro-Lys-His-Asn-Pro-Gly-Val-Hyp-Hyp-Ala-OH (20)

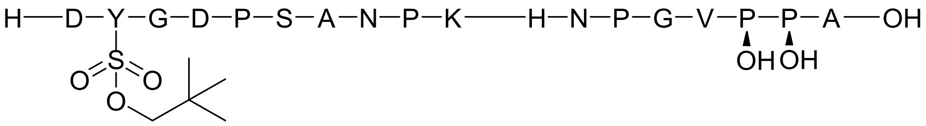

Compound **20** was obtained by the general procedure for peptide synthesis, using the automatic peptide synthesizer. For Hyp coupling, commercially available Fmoc-Hyp(*t*Bu)-OH **9** was used. For sulfated tyrosine coupling, commercially available Fmoc-Tyr(SO$_2$ONp)-OH (Bachem) was used. Crude peptide was purified using preparative-HPLC and collected fractions were lyophilized. Purified peptide was further used for deprotection of the sulfated tyrosine (**1**).

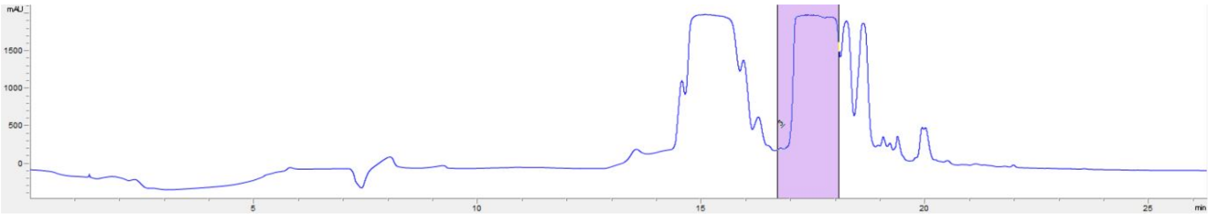

Figure S2.1. Prep-HPLC trace of the purification of peptide **20**. The section that is indicated with the purple box was collected and processed further. Instrument: Agilent 1260 Preparative HPLC, Column: Grace Alltima column, C18, 5 µ, 22 mm x 250 mm, Eluent: 0.1 % FA in H_2O-CH_3CN, Gradient: 5→5→95→95→5→5% (percentage CH_3CN) (0→5→25→30→35→40 min), Flow Rate: 10 mL/min, Detection: DAD at 415 nm, retention time (t_R) of product **20**: 16.7–18.4 min.

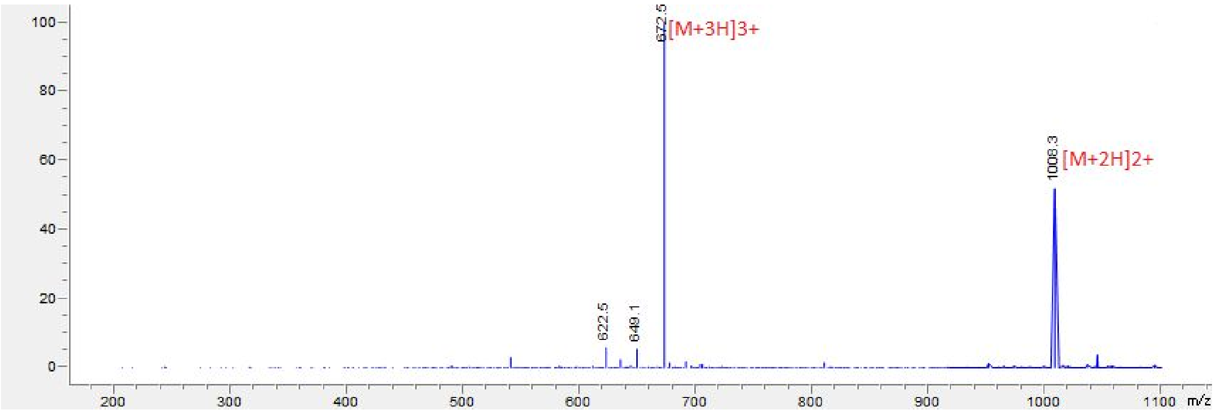

Figure S2.2. MS spectrum of the fraction that was collected from the prep-HPLC trace shown in Fig. **S2.1**. The peaks are assigned as follows: MS (ESI): *m/z* = [M+3H]$^{3+}$ calc for $C_{85}H_{130}N_{23}O_{32}S$ 672.3, found 672.3; *m/z* = [M+2H]$^{2+}$ calc for $C_{85}H_{129}N_{23}O_{32}S$ 1007.9, found 1007.9.

H-Asp-Tyr(SO$_2$ONp)-Gly-Asp-Pro-Ser-Ala-Asn-Pro-Lys-His-Asn-Pro-Gly-Val-Hyp[Ara(OAc)]$_1$-Hyp-Ala-OH (21)

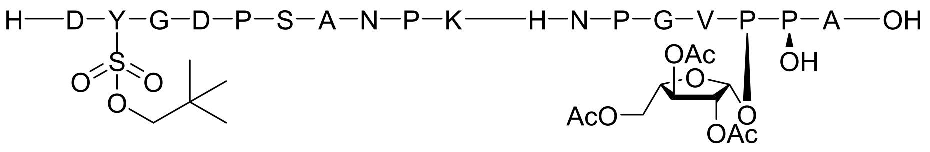

Peptide chain elongation of **17** was further completed with the use of an automatic peptide synthesizer. The product was then cleaved from the resin, diluted in MilliQ and lyophilized. Before purification, the crude product was analyzed with LC-MS (LXQ). Found: $[M+3H]^{3+}$ 758.32, $[M+2H]^{2+}$ 1136.98. Lyophilized crude product (52 mg) was purified by semi-preparative HPLC using a 100% H_2O + 0.1% FA to 100% MeCN + 0.1% FA gradient yielding in title compound **21** (4.7 mg, 4.7%).

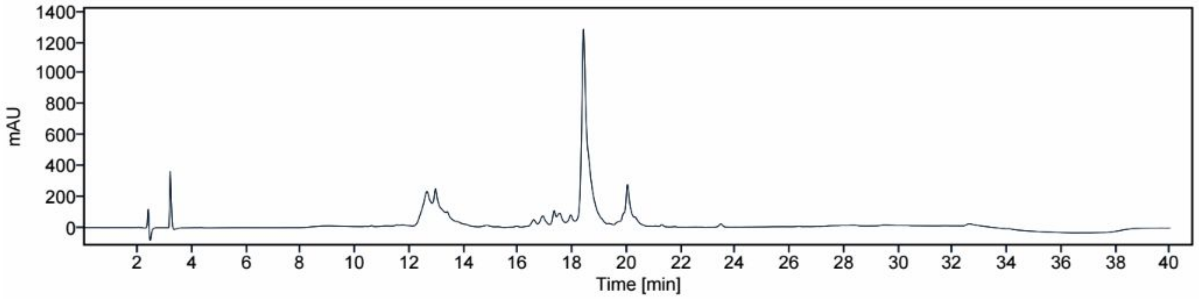

Figure S3.1. UHPLC trace of the purified peptide **21**. The t_R of the product is 18.4 min.

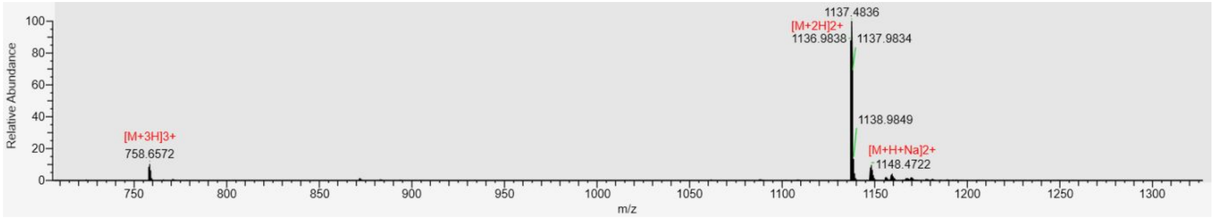

Figure S3.2. MS spectrum of the purified peptide **21**. Spectrum belongs to the LC peak with rt 18.4 min (shown in Fig. **S3.1**). The peaks are assigned as follows: HRMS (ESI): *m/z* = $[M+3H]^{3+}$ calc for $C_{96}H_{144}N_{23}O_{39}S$ 758.3232, found 758.3231; *m/z* = $[M+2H]^{2+}$ calc for $C_{96}H_{143}N_{23}O_{39}S$ 1136.9812, found 1136.9838; *m/z* = $[M+H+Na]^{2+}$ calc for $C_{96}H_{142}N_{23}O_{39}SNa$ 1147.9721, found 1147.9707.

H-Asp-Tyr(SO_2ONp)-Gly-Asp-Pro-Ser-Ala-Asn-Pro-Lys-His-Asn-Pro-Gly-Val-Hyp[Ara(OAc)]$_2$-Hyp-Ala-OH (22)

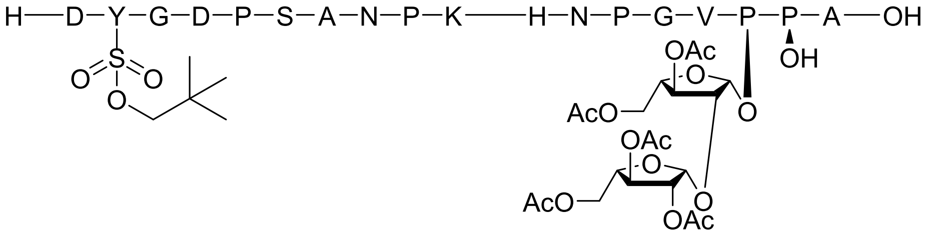

Peptide chain elongation of **18** was further completed with the use of an automatic peptide synthesizer. The product was then cleaved from the resin, diluted in MilliQ and lyophilized. Before purification, the crude product was analyzed with LC-MS (LXQ). Found: $[M+3H]^{3+}$ 830.34, $[M+2H]^{2+}$ 1245.01. Lyophilized crude product (52 mg) was purified by semi-preparative HPLC using a 100% H_2O + 0.1% FA to 40% MeCN + 0.1% FA gradient yielding in title compound **22** (16.6 mg, 12.7%).

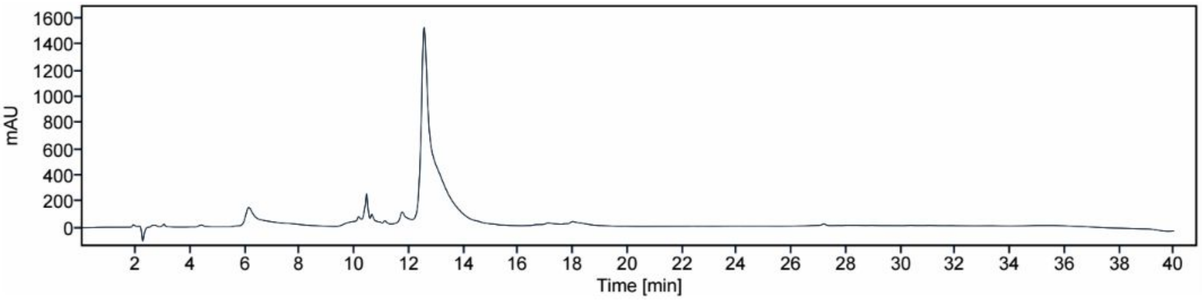

Figure S4.1. UHPLC trace of the purified peptide **22**. The t_R of the product is 12.5 min.

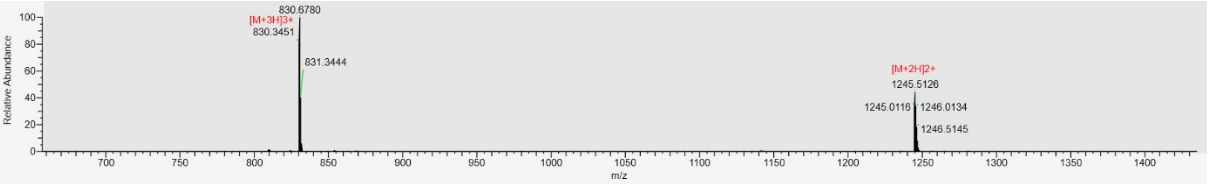

Figure S4.2. MS spectrum of the purified peptide **22**. Spectrum belongs to the LC peak with t_R 12.5 min (shown in Fig. **S4.1**). The peaks are assigned as follows: HRMS (ESI): *m/z* = $[M+3H]^{3+}$ calc for $C_{105}H_{156}N_{23}O_{45}S$ 830.3443, found 830.3451; HRMS (ESI): *m/z* = $[M+2H]^{2+}$ calc for $C_{105}H_{155}N_{23}O_{45}S$ 1245.0123, found 1245.0116.

H-Asp-Tyr(SO_2ONp)-Gly-Asp-Pro-Ser-Ala-Asn-Pro-Lys-His-Asn-Pro-Gly-Val-Hyp[Ara(OAc)]$_3$-Hyp-Ala-OH (23)

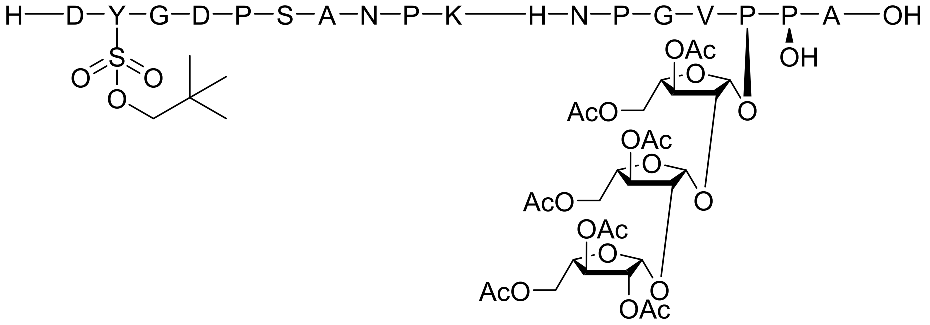

Peptide chain elongation of **19** was further completed with the use of an automatic peptide synthesizer. The product was then cleaved from the resin, diluted in MilliQ and lyophilized. Before purification, the crude product was analyzed with LC-MS (LXQ). Found: $[M+3H]^{3+}$ 902.36, $[M+2H]^{2+}$ 1353.04. Lyophilized crude product (11 mg) was purified by semi-preparative HPLC using a 100% H_2O + 0.1% FA to 40% MeCN + 0.1% FA gradient yielding in title compound **23** (3.6 mg, 5.3%).

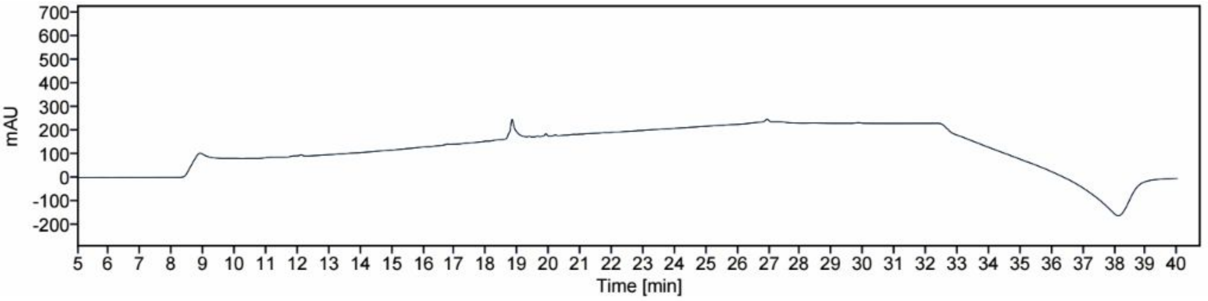

Figure S5.1. UHPLC trace of the purified peptide **23**. The t_R of the product is 18.8 min.

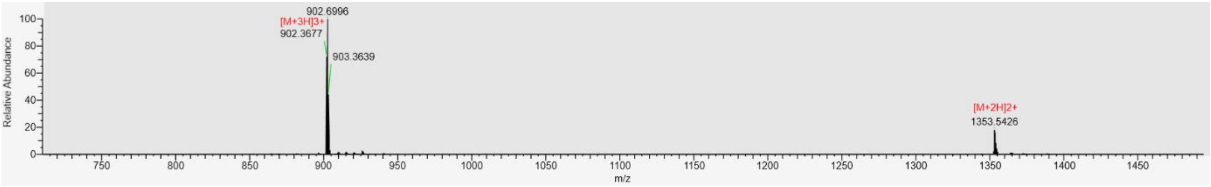

Figure S5.2 MS spectrum of the purified peptide **23**. Spectrum belongs to the LC peak with t_R 18.8 min (shown in Fig. **S5.1**). The peaks are assigned as follows: HRMS (ESI): m/z = $[M+3H]^{3+}$ calc for $C_{114}H_{168}N_{23}O_{51}S$ 902.3654, found 902.3677; m/z = $[M+2H]^{2+}$ calc for $C_{114}H_{167}N_{23}O_{51}S$ 1353.0445, found 1353.0415.

H-Asp-Tyr(SO$_2$ONp)-Gly-Asp-Pro-Ser-Ala-Asn-Pro-Lys-His-Asn-Pro-Gly-Val-Hyp[Ara$_1$]-Hyp-Ala-OH (24)

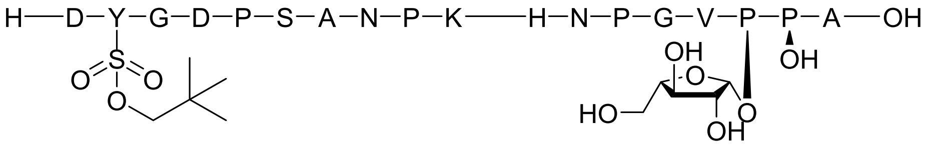

Lyophilized *O*-acetylated glycopeptide **21** (3.5 mg, 1.5 µmol) was dissolved in NaOMe in MeOH (2 mL supplemented with 100 eq., 0.15 mmol NaOMe). The reaction was monitored by UHPLC-MS (Q Exactive) and found to be complete overnight. The product was then diluted with MilliQ and lyophilized to obtain glycopeptide **24** (3.2 mg, 1.5µmol, quant.).

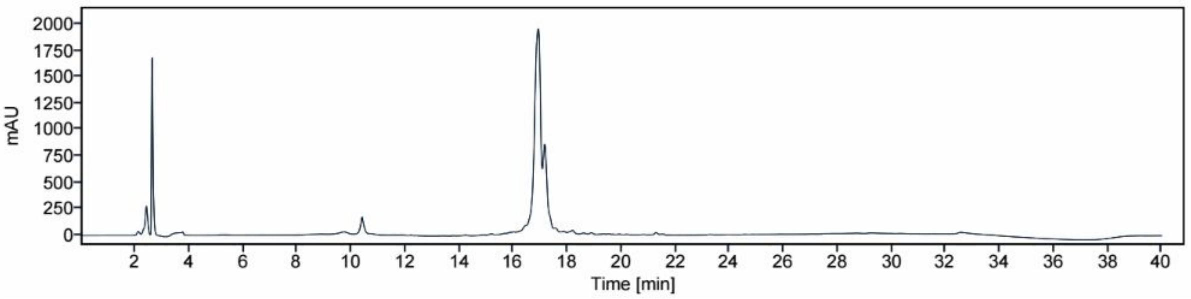

Figure S6.1. UHPLC trace of peptide **24**. The t_R of the product is 16.9 min.

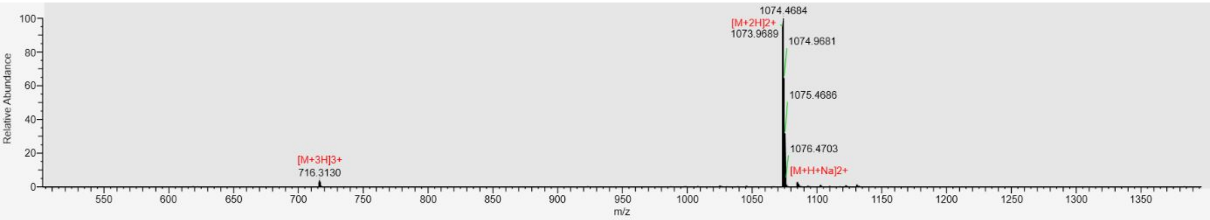

Figure S6.2. MS spectrum of peptide **24**. Spectrum belongs to the LC peak with t_R 18.4 min (shown in Fig. **S6.1**). The peaks are assigned as follows: HRMS (ESI): *m/z* = $[M+3H]^{3+}$ calc for $C_{90}H_{138}N_{23}O_{36}S$ 716.3126, found 716.3130; *m/z* = $[M+2H]^{2+}$ calc for $C_{90}H_{137}N_{23}O_{36}S$ 1073.9653, found 1073.9689; *m/z* = $[M+H+Na]^{2+}$ calc for $C_{90}H_{136}N_{23}O_{36}SNa$ 1084.9563, found 1084.9561.

H-Asp-Tyr(SO$_2$ONp)-Gly-Asp-Pro-Ser-Ala-Asn-Pro-Lys-His-Asn-Pro-Gly-Val-Hyp[Ara$_2$]-Hyp-Ala-OH (25)

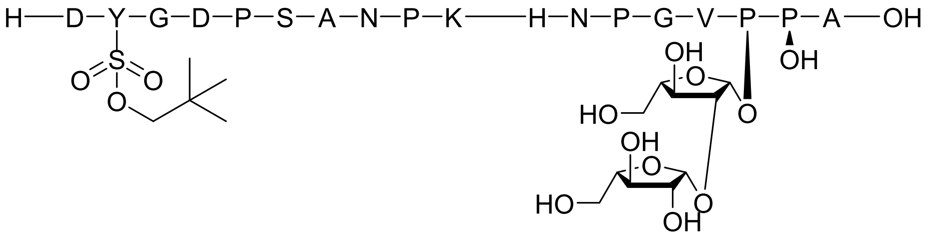

Lyophilized *O*-acetylated glycopeptide **22** (16.6 mg, 6.6 µmol) was dissolved in NaOMe in MeOH (4 mL supplemented with 100 eq., 0.66 mmol NaOMe). The reaction was monitored by UHPLC-MS (Q Exactive) and found to be complete overnight. The product was then diluted with MilliQ and lyophilized to obtain glycopeptide **25** (15.2 mg, 6.6 µmol, quant.).

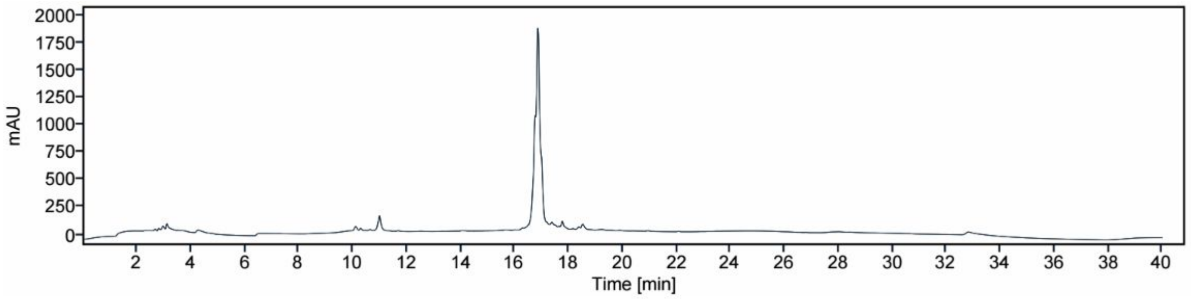

Figure S7.1. UHPLC trace of peptide **25**. The t_R of the product is 16.8 min.

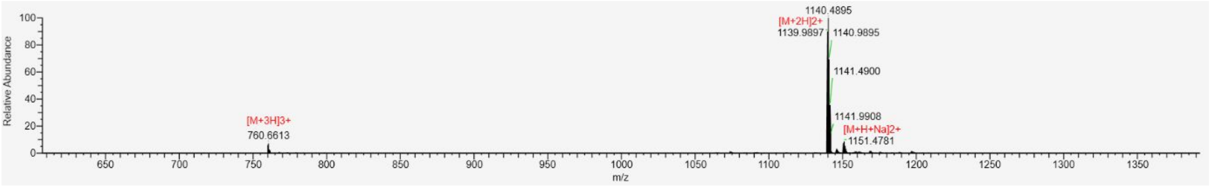

Figure S7.2. MS spectrum of peptide **25**. Spectrum belongs to the LC peak with t_R 16.8 min (shown in Fig. **S7.1**). The peaks are assigned as follows: HRMS (ESI): *m/z* = $[M+3H]^{3+}$ calc for $C_{95}H_{146}N_{23}O_{40}S$ 760.3267, found 760.3270; *m/z* = $[M+2H]^{2+}$ calc for $C_{95}H_{145}N_{23}O_{40}S$ 1139.9864, found 1139.9897; *m/z* = $[M+H+Na]^{2+}$ calc for $C_{95}H_{144}N_{23}O_{40}SNa$ 1150.9774, found 1150.9769.

H-Asp-Tyr(SO$_2$ONp)-Gly-Asp-Pro-Ser-Ala-Asn-Pro-Lys-His-Asn-Pro-Gly-Val-Hyp[Ara$_3$]-Hyp-Ala-OH (26)

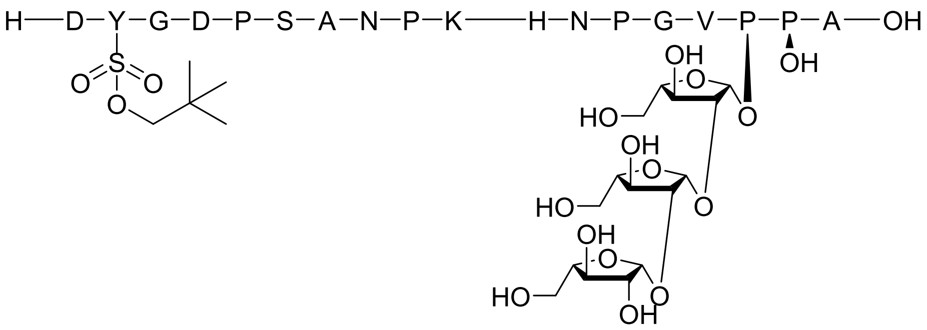

Lyophilized *O*-acetylated glycopeptide **23** (3.6 mg, 1.3 µmol) was dissolved in NaOMe in MeOH (2 mL supplemented with 100 eq., 0.13 mmol NaOMe). The reaction was monitored by UHPLC-MS (Q Exactive) and found to be complete overnight. The product was then diluted with MilliQ and lyophilized to obtain glycopeptide **26** (3.2 mg, 1.3 µmol, quant.).

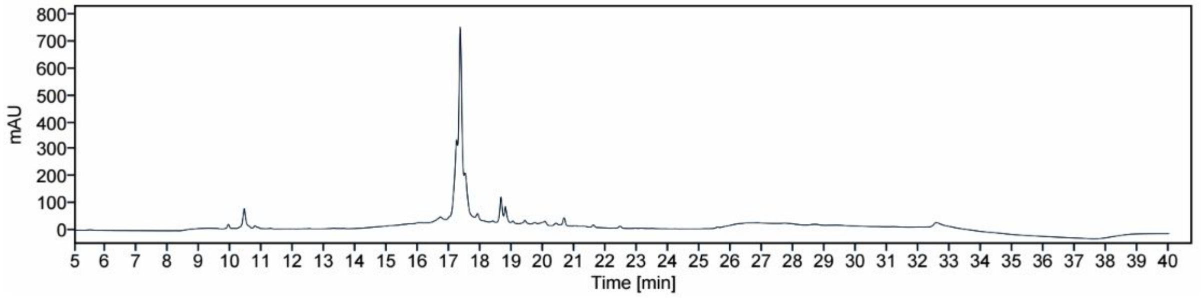

Figure S8.1. UHPLC trace of peptide **26**. The t_R of the product is 17.3 min.

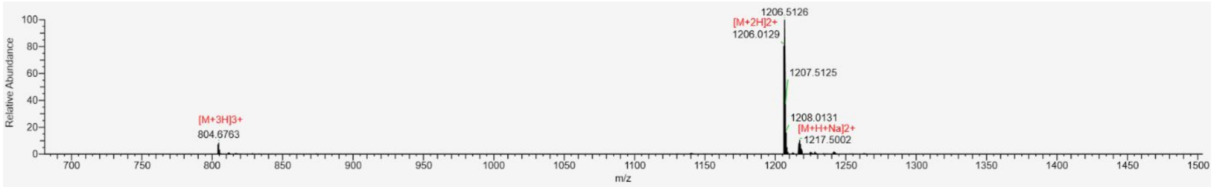

Figure S8.2. MS spectrum of peptide **26**. Spectrum belongs to the LC peak with t_R 17.3 min (shown in Fig. **S8.1**). The peaks are assigned as follows: HRMS (ESI): *m/z* = $[M+3H]^{3+}$ calc for $C_{100}H_{154}N_{23}O_{44}S$ 804.3408, found 804.3420; *m/z* = $[M+2H]^{2+}$ calc for $C_{100}H_{153}N_{23}O_{44}S$ 1206.0076, found 1206.0129; *m/z* = $[M+H+Na]^{2+}$ calc for $C_{100}H_{152}N_{23}O_{44}SNa$ 1216.9985, found 1216.9990.

H-Asp-Tyr(SO$_3$H)-Gly-Asp-Pro-Ser-Ala-Asn-Pro-Lys-His-Asn-Pro-Gly-Val-Hyp-Hyp-Ala-OH (1)

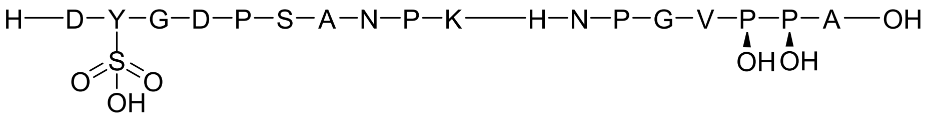

Compound **20** was treated with 2M NH_4OAc for the removal of neopentyl from the sulfated tyrosine residue. The solution was allowed to react for 40 hours at 45 °C prior to lyophilization. The resulting mixture was purified with the Agilent 1260 preparative HPLC using a Grace Alltima C18 column. For the sulfate deprotected peptide, buffers were used containing 10 mM NH_4OAc. Purification was performed with a gradient from 95% H_2O to 60% MeCN in 20 min with a 10 mL/min flow rate. Collected fractions containing the product were lyophilized to afford *Brassica* PSY1 16-Hyp **1**.

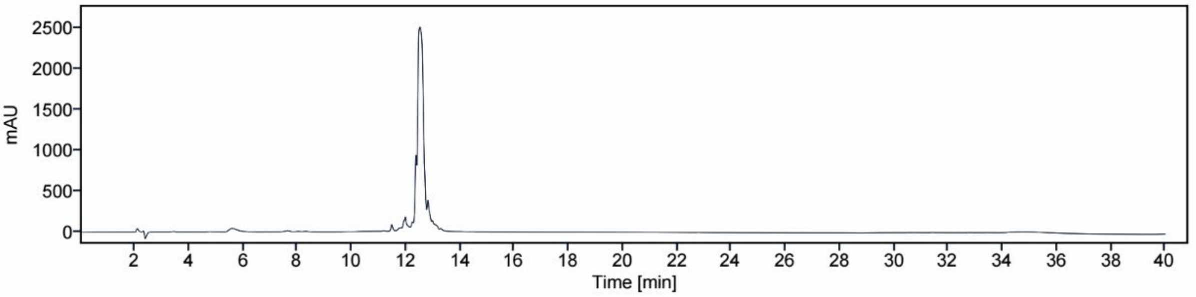

Figure S9.1. UHPLC trace of peptide **1**. The t_R of the product is 12.5 min.

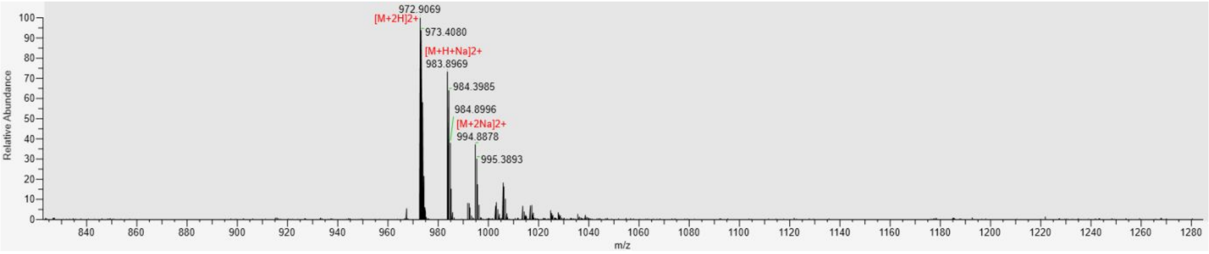

Figure S9.2. MS spectrum of peptide **1**. Spectrum belongs to the LC peak with t_R 12.5 min (shown in Fig. **S9.1**). The peaks are assigned as follows: HRMS (ESI): *m/z* = $[M+2H]^{2+}$ calc for $C_{80}H_{119}N_{23}O_{32}S$ 972.9051, found 972.9069; *m/z* = $[M+H+Na]^{2+}$ calc for $C_{80}H_{119}N_{23}O_{32}SNa$ 983.8960, found 983.8969; *m/z* = $[M+2Na]^{2+}$ calc for $C_{80}H_{117}N_{23}O_{32}SNa_2$ 994.8870, found 994.8878.

H-Asp-Tyr(SO$_3$H)-Gly-Asp-Pro-Ser-Ala-Asn-Pro-Lys-His-Asn-Pro-Gly-Val-Hyp[Ara$_1$]-Hyp-Ala-OH (2)

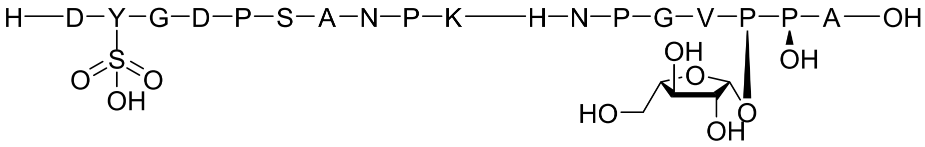

Lyophilized Tyr(SO$_3$Np) glycopeptide **24** (3.2 mg, 1.5 µmol) was dissolved in 2M NH$_4$OAc (2 mL) and was left at 45 °C. The reaction was monitored by UHPLC-MS (Q Exactive) and found to be complete after 40 hours. The product was then diluted with MilliQ and lyophilized to obtain *Brassica* PSY1 16-Ara$_1$Hyp **2** (3.1 mg, 1.5 µmol, quant.).

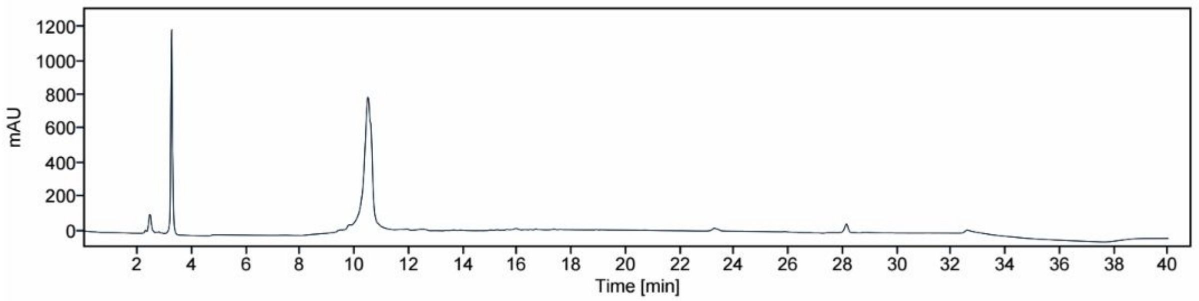

Figure S10.1. UHPLC trace of peptide **2**. The t_R of the product is 10.5 min.

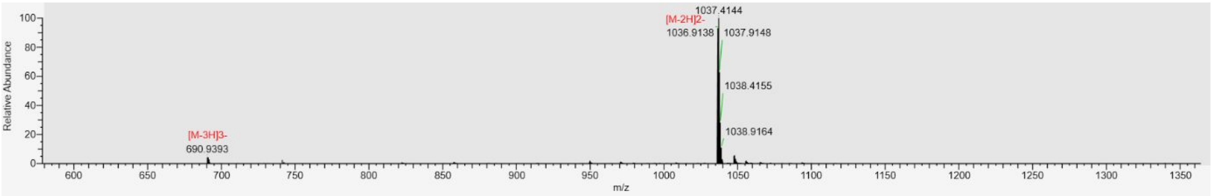

Figure S10.2. MS spectrum of peptide **2**. Spectrum belongs to the LC peak with t_R 10.5 min (shown in Fig **S10.1**). The peaks are assigned as follows: HRMS (ESI): *m/z* = [M-3H]$^{3-}$ calc for $C_{85}H_{122}N_{23}O_{36}S$ 690.9387, found 690.9393; *m/z* = [M-2H]$^{2-}$ calc for $C_{85}H_{123}N_{23}O_{36}S$ 1036.9116, found 1036.9138.

H-Asp-Tyr(SO$_3$H)-Gly-Asp-Pro-Ser-Ala-Asn-Pro-Lys-His-Asn-Pro-Gly-Val-Hyp[Ara$_2$]-Hyp-Ala-OH (3)

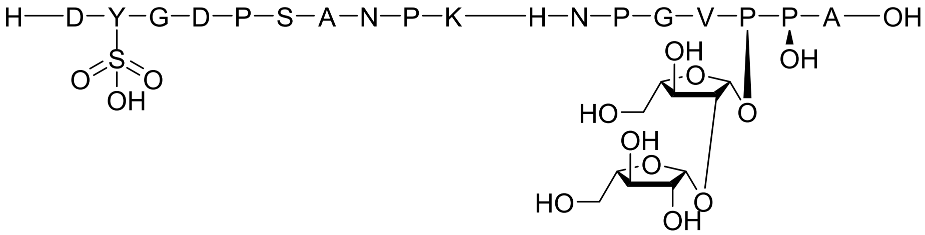

Lyophilized Tyr(SO$_3$Np) glycopeptide **25** (15.2 mg, 6.6 µmol) was dissolved in 2M NH$_4$OAc (4 mL) and was left at 45 °C. The reaction was monitored by UHPLC-MS (Q Exactive) and found to be complete after 24 hrs. The product was then diluted with MilliQ and lyophilized to obtain *Brassica* PSY1 16-Ara$_2$Hyp **3** (14.8 mg, 6.6 µmol, quant.).

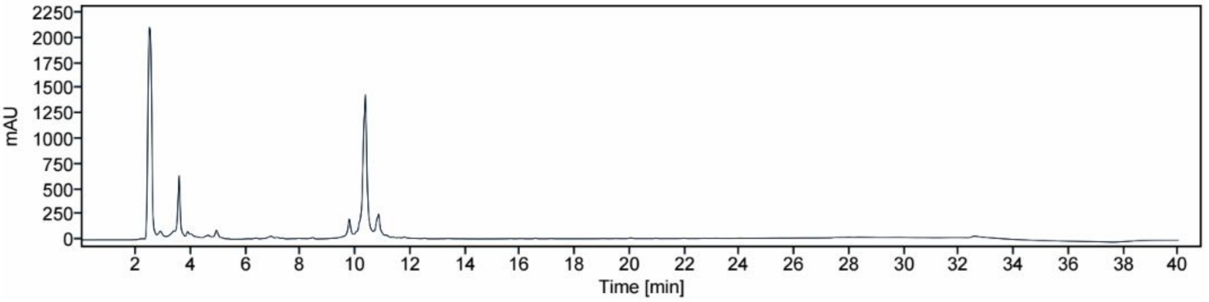

Figure S11.1. UHPLC trace of peptide **3**. The t_R of the product is 10.3 min.

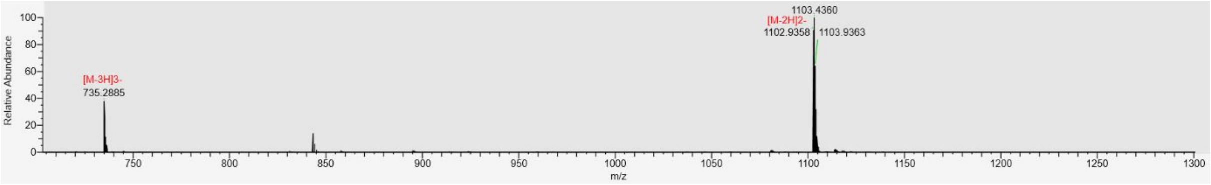

Figure S11.2. MS spectrum of peptide **3**. Spectrum belongs to the LC peak with t_R 10.3 min (shown in Fig. **S11.1**). The peaks are assigned as follows: HRMS (ESI): *m/z* = [M-3H]$^{3-}$ calc for $C_{90}H_{130}N_{23}O_{40}S$ 734.9528, found 734.9547; *m/z* = [M-2H]$^{2-}$ calc for $C_{90}H_{131}N_{23}O_{40}S$ 1102.9328, found 1102.9358.

H-Asp-Tyr(SO$_3$H)-Gly-Asp-Pro-Ser-Ala-Asn-Pro-Lys-His-Asn-Pro-Gly-Val-Hyp[Ara$_3$]-Hyp-Ala-OH (4)

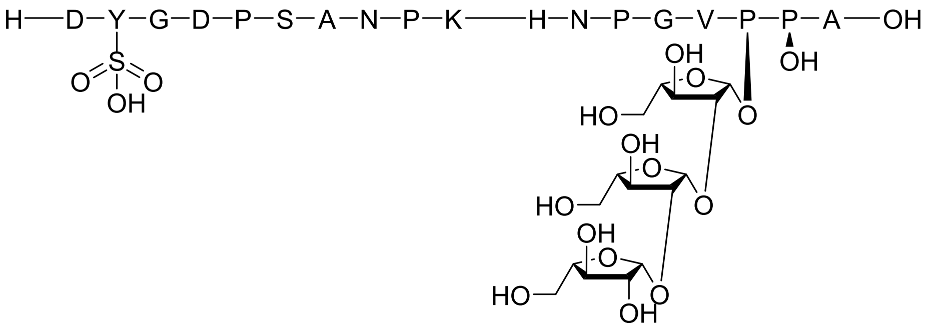

Lyophilized Tyr(SO$_3$Np) glycopeptide **25** (3.2 mg, 1.3 µmol) was dissolved in 2M NH$_4$OAc (2 mL) and was left at 45 °C. The reaction was monitored by UHPLC-MS (Q Exactive) and found to be complete after 24 hours. The product was then diluted with MilliQ and lyophilized to obtain *Brassica* PSY1 16-Ara$_3$Hyp **4** (3.1 mg, 1.3 µmol, quant.).

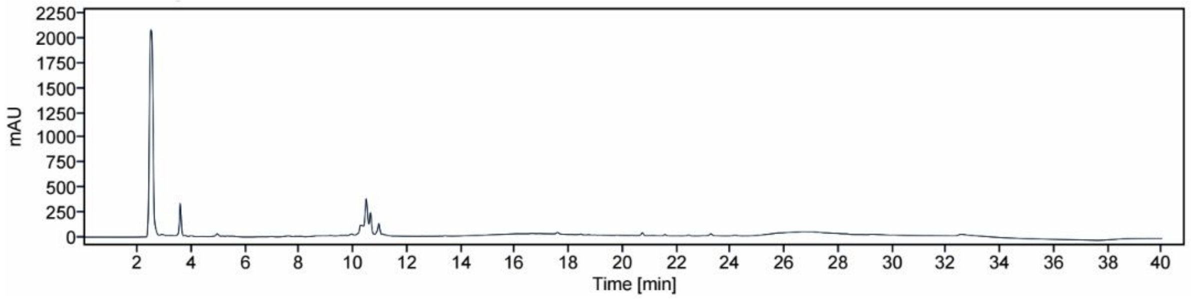

Figure S12.1. UHPLC trace of peptide **4**. All peaks in the 10–11 min range correspond to the m/z value of the target compound, indicating the presence of isomers of the final tri-arabinosylated sulfopeptide **25**.

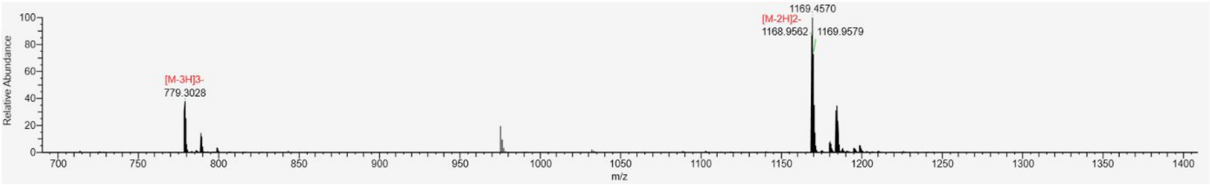

Figure S12.2. MS spectrum of peptide **4**. Spectrum belongs to the LC peak with t_R 10.5 min (shown in Fig. **S12.1**). The peaks are assigned as follows: HRMS (ESI): *m/z* = $[M-3H]^{3-}$ calc for $C_{95}H_{138}N_{23}O_{44}S$ 778.9668, found 778.9686; *m/z* = $[M-2H]^{2-}$ calc for $C_{95}H_{139}N_{23}O_{44}S$ 1168.9539, found 1168.9562.

^{1}H and ^{13}C-NMR spectra of all compounds

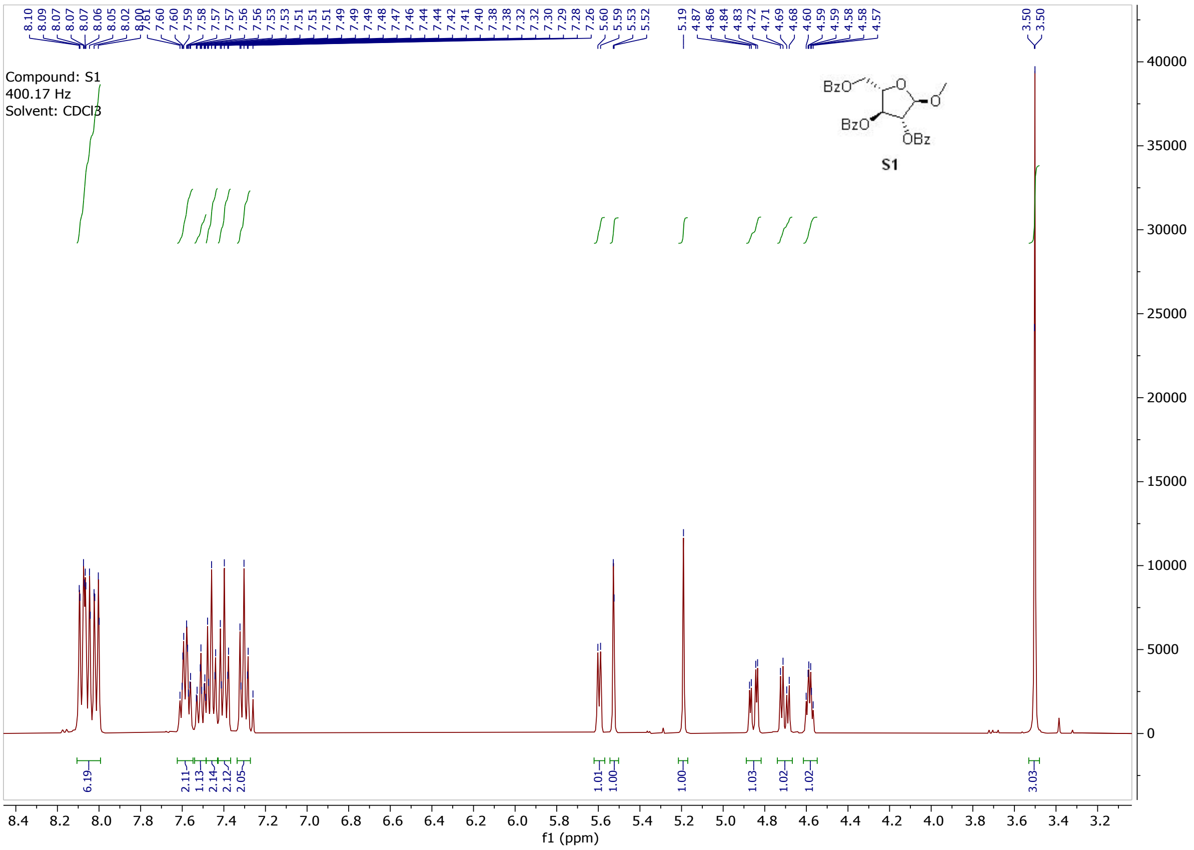

Figure S13. ^{1}H spectrum of methyl 2,3,5-tri-*O*-benzoyl-α-L-arabinofuranoside (**S1**).

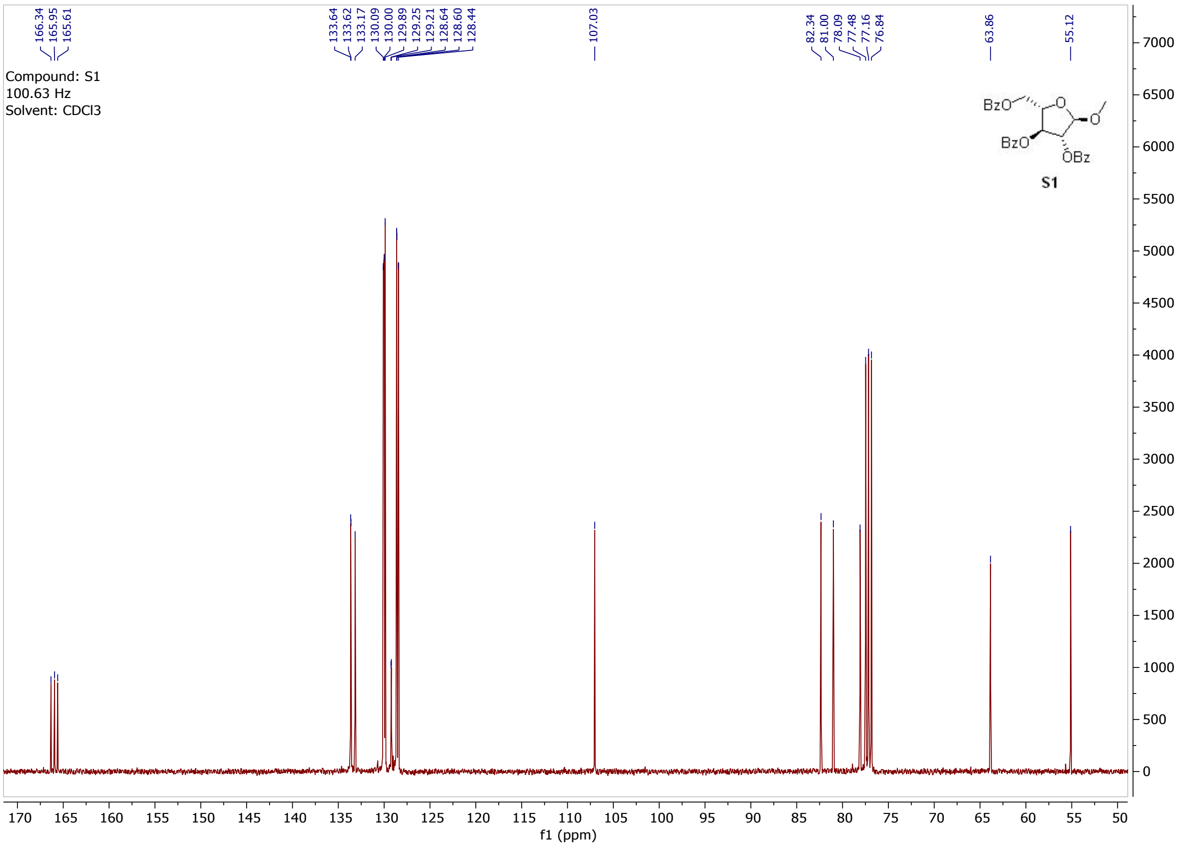

Figure S14. ^{13}C spectrum of methyl 2,3,5-tri-*O*-benzoyl-α-L-arabinofuranoside (**S1**).

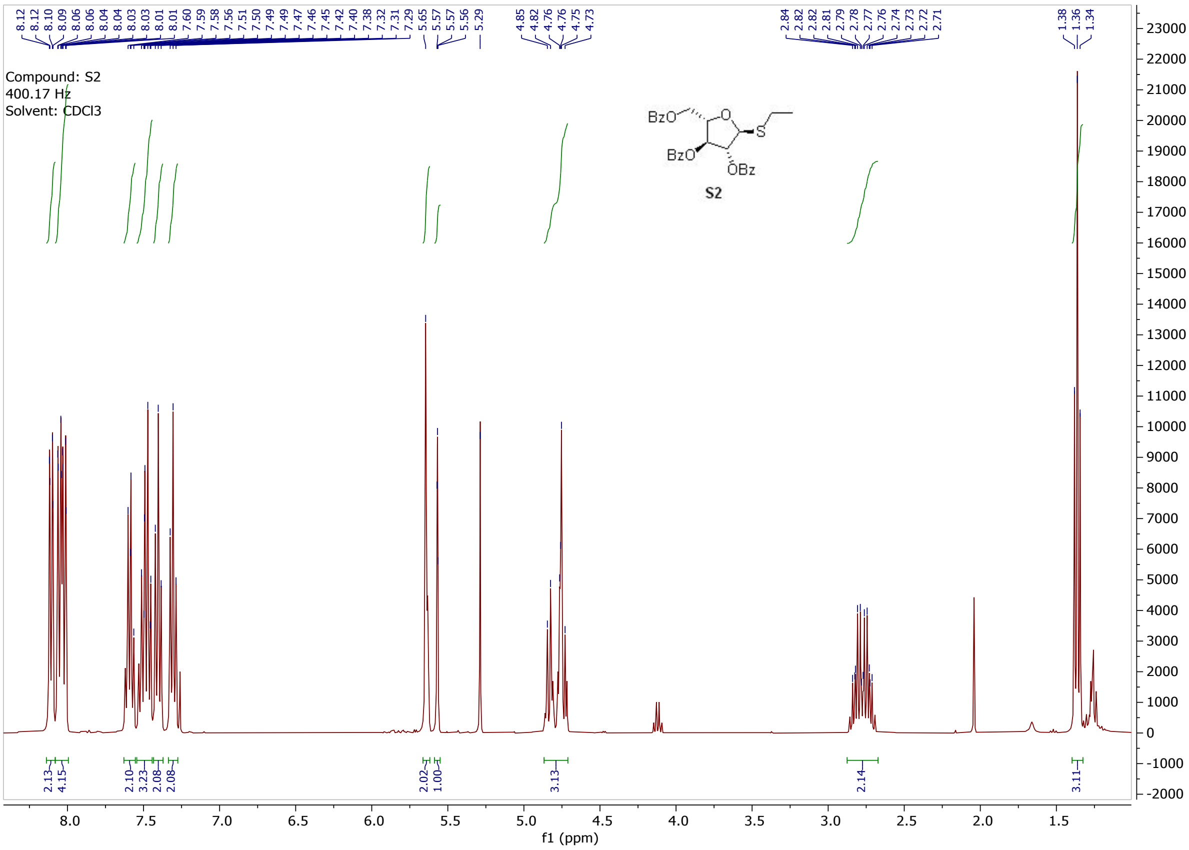

Figure S15. ^{1}H spectrum of ethyl 2,3,5-tri-*O*-benzoyl-1-thio-α-L-arabinofuranoside (**S2**).

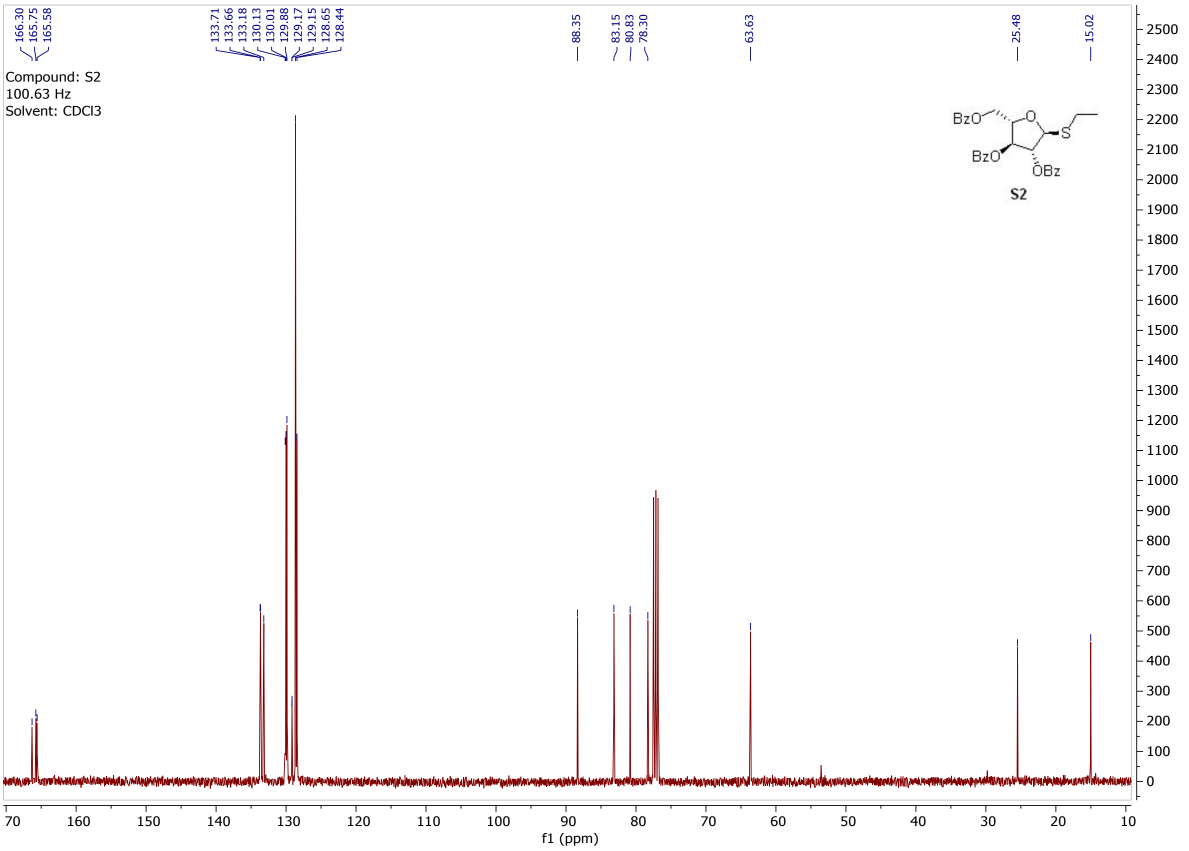

Figure S16. ^{13}C spectrum of ethyl 2,3,5-tri-*O*-benzoyl-1-thio-α-L-arabinofuranoside (**S2**).

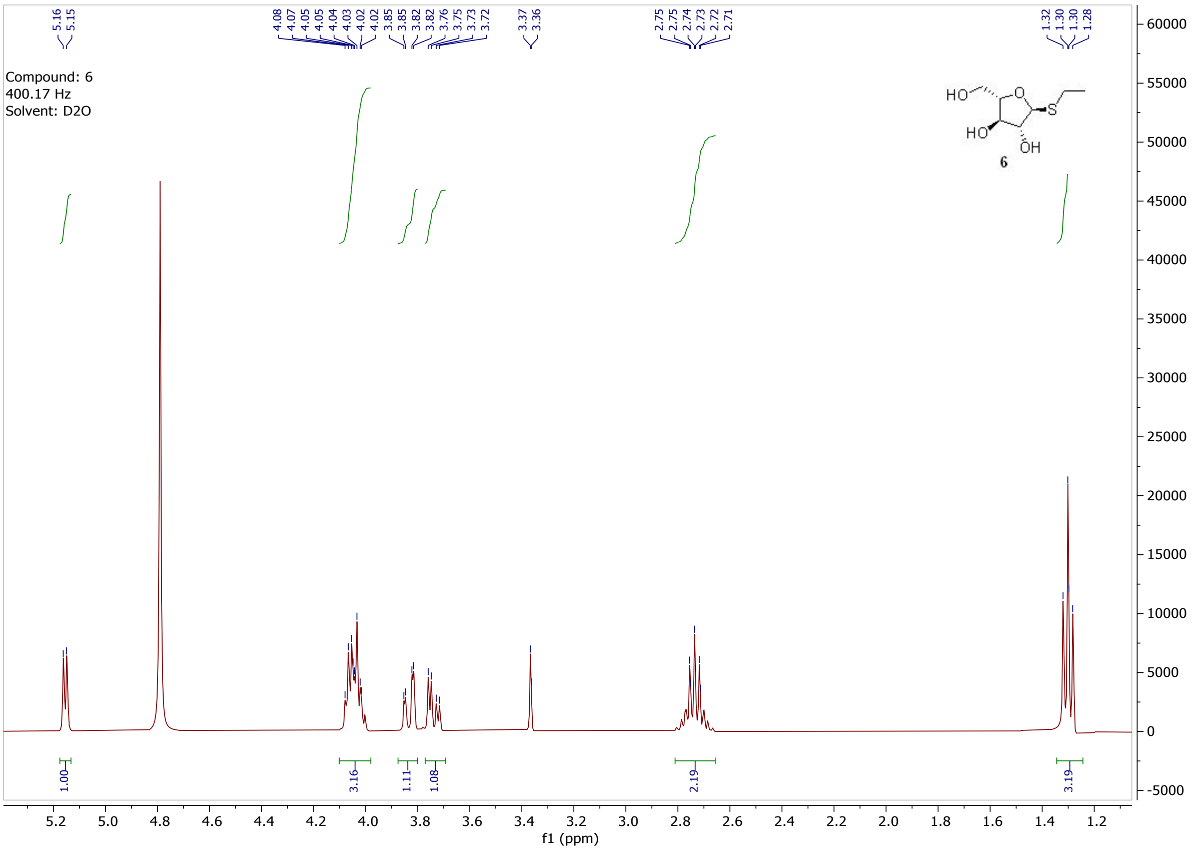

Figure S17. ^{1}H spectrum of ethyl 1-thio-α-L-arabinofuranoside (**6**).

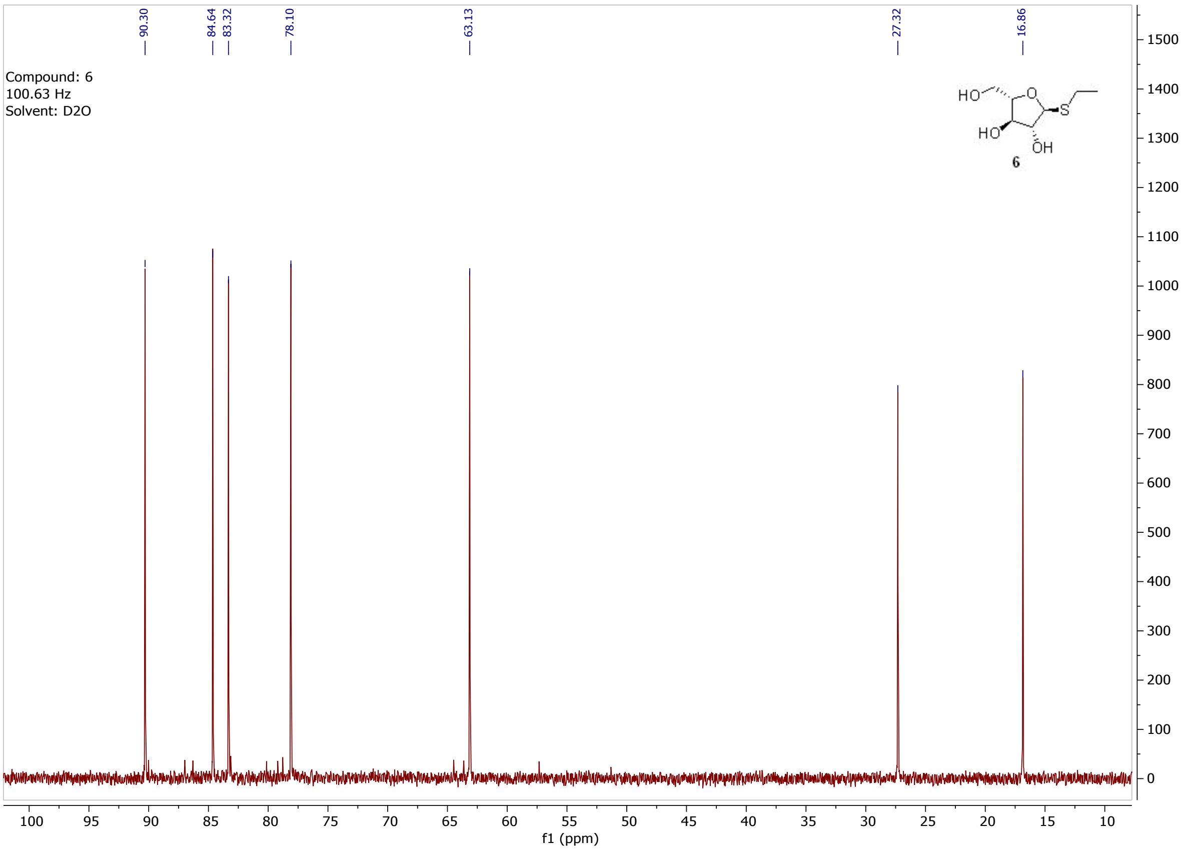

Figure S18. ^{13}C spectrum of ethyl 1-thio-α-L-arabinofuranoside (**6**).

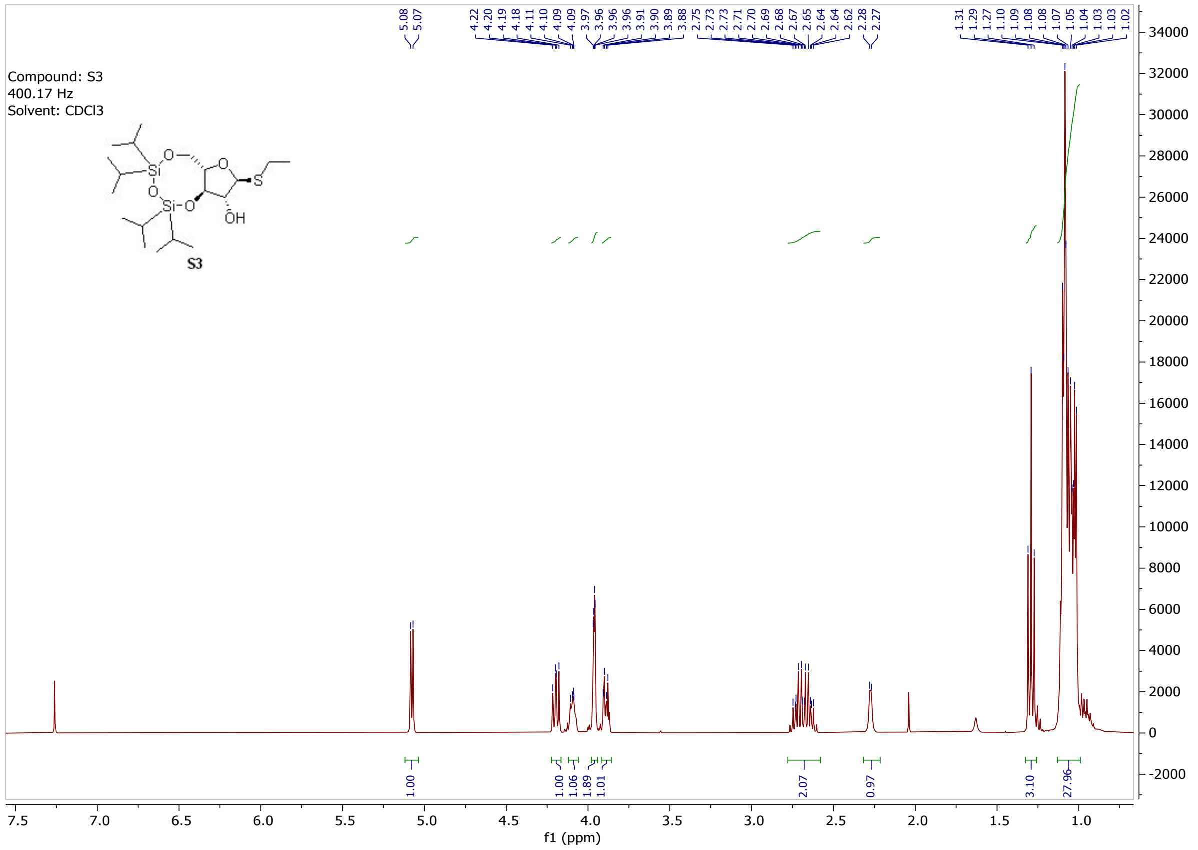

Figure S19. ^{1}H spectrum of ethyl 3,5-O-TIPS-1-thio-α-L-arabinofuranoside (**S3**).

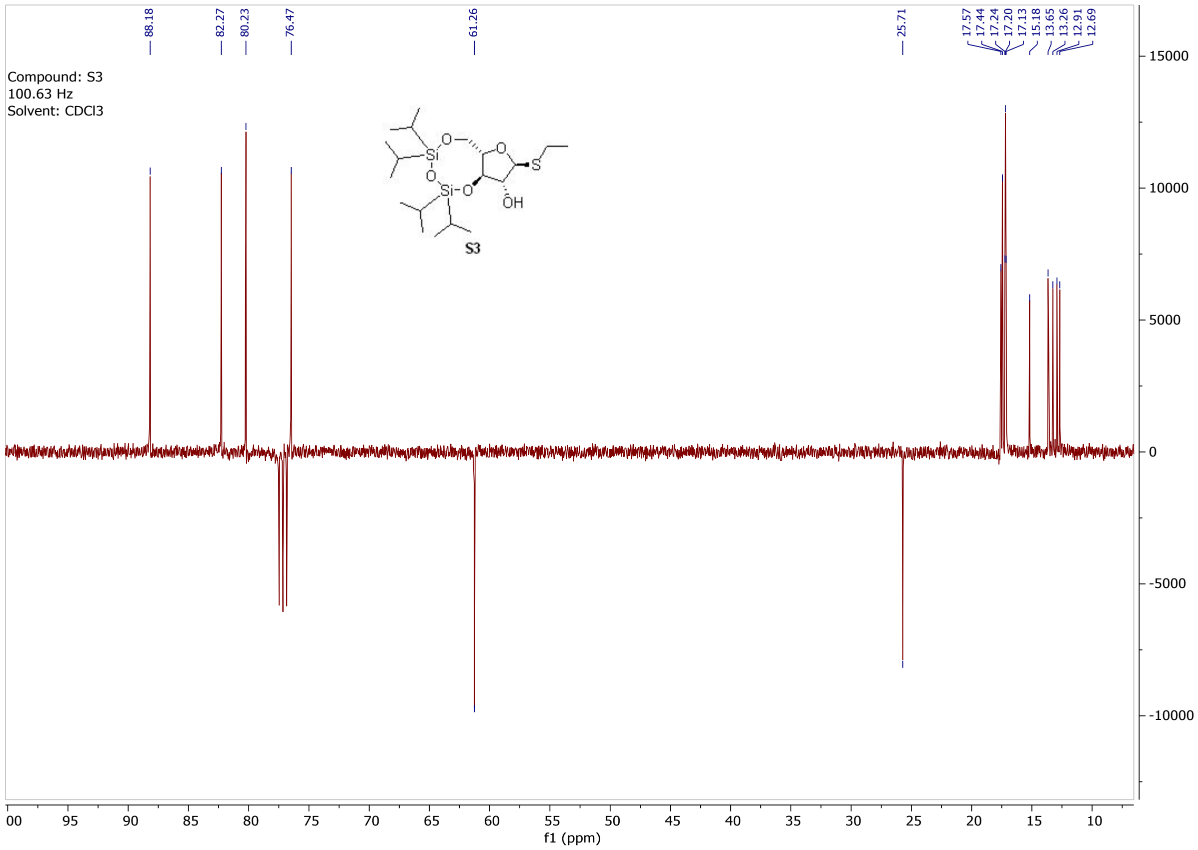

Figure S20. ^{13}C APT spectrum of ethyl 3,5-O-TIPS-1-thio-α-L-arabinofuranoside (**S3**).

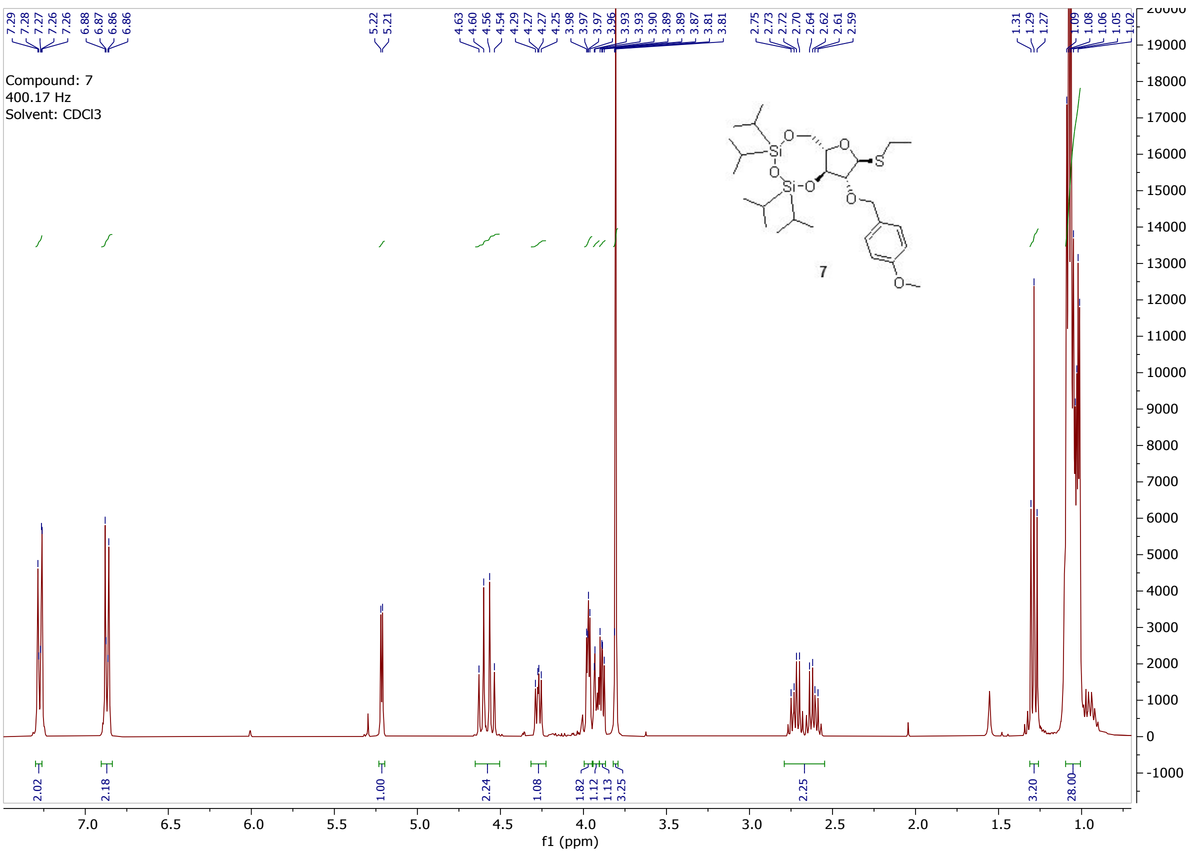

Figure S21. ^{1}H spectrum of ethyl 2-O-PMB-3,5-O-TIPS-1-thio-α-L-arabinofuranoside (**7**).

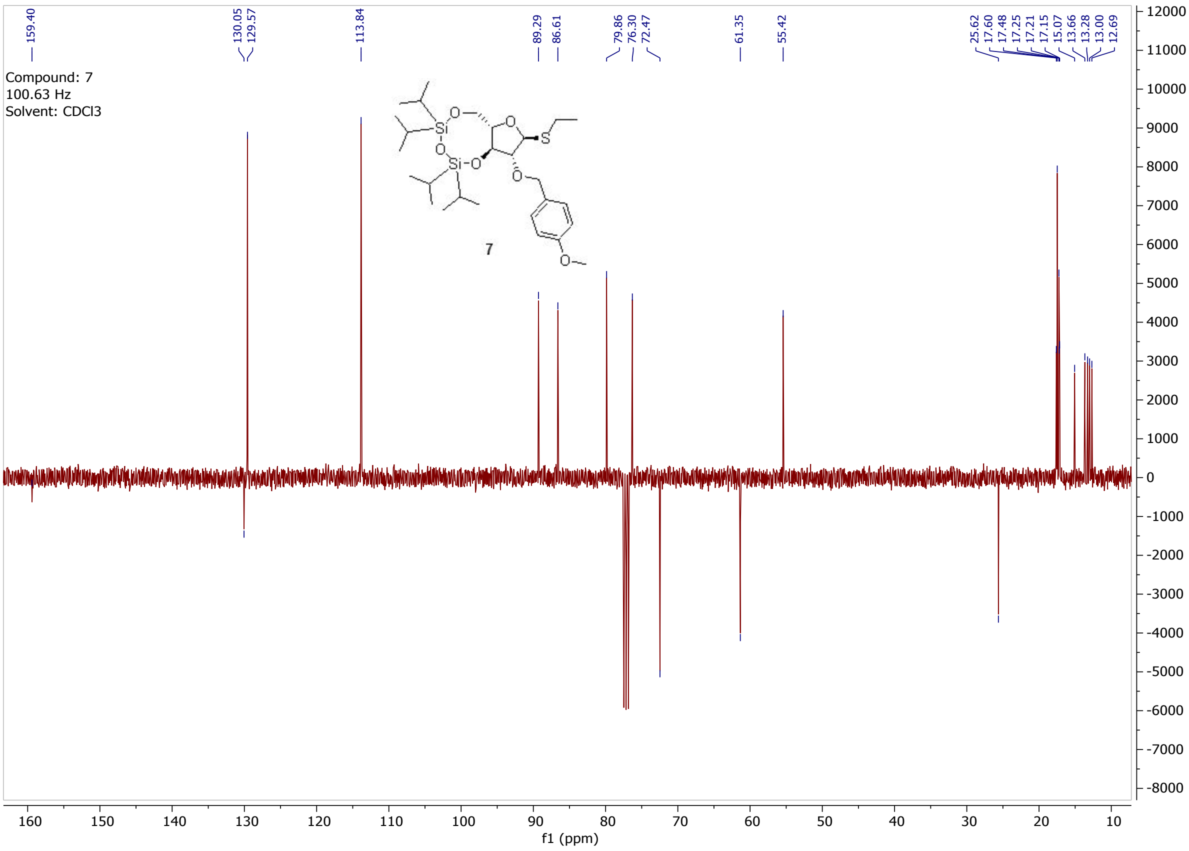

Figure S22. ^{13}C APT spectrum of ethyl 2-O-PMB-3,5-O-TIPS-1-thio-α-L-arabinofuranoside (**7**).

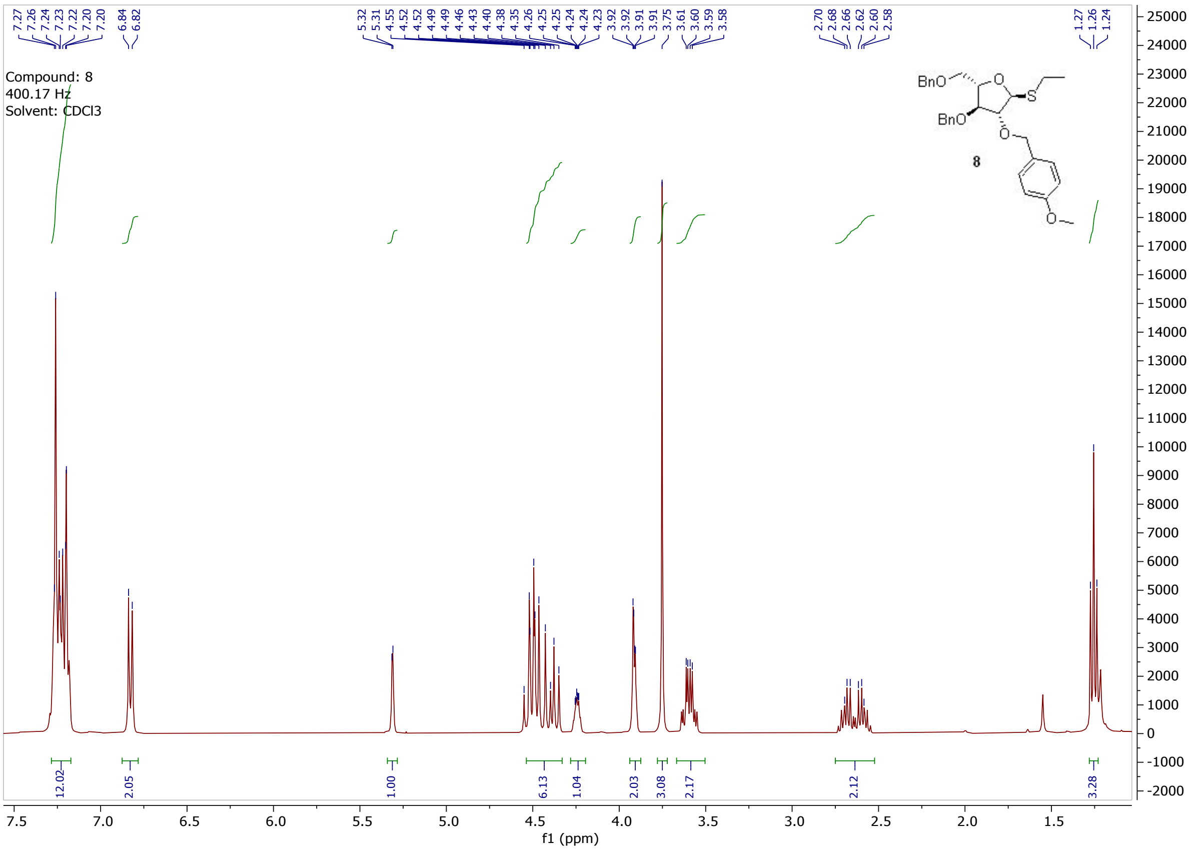

Figure S23. ^{1}H spectrum of ethyl 2-O-PMB-3,5-O-benzyl-1-thio-α-L-arabinofuranoside (**8**).

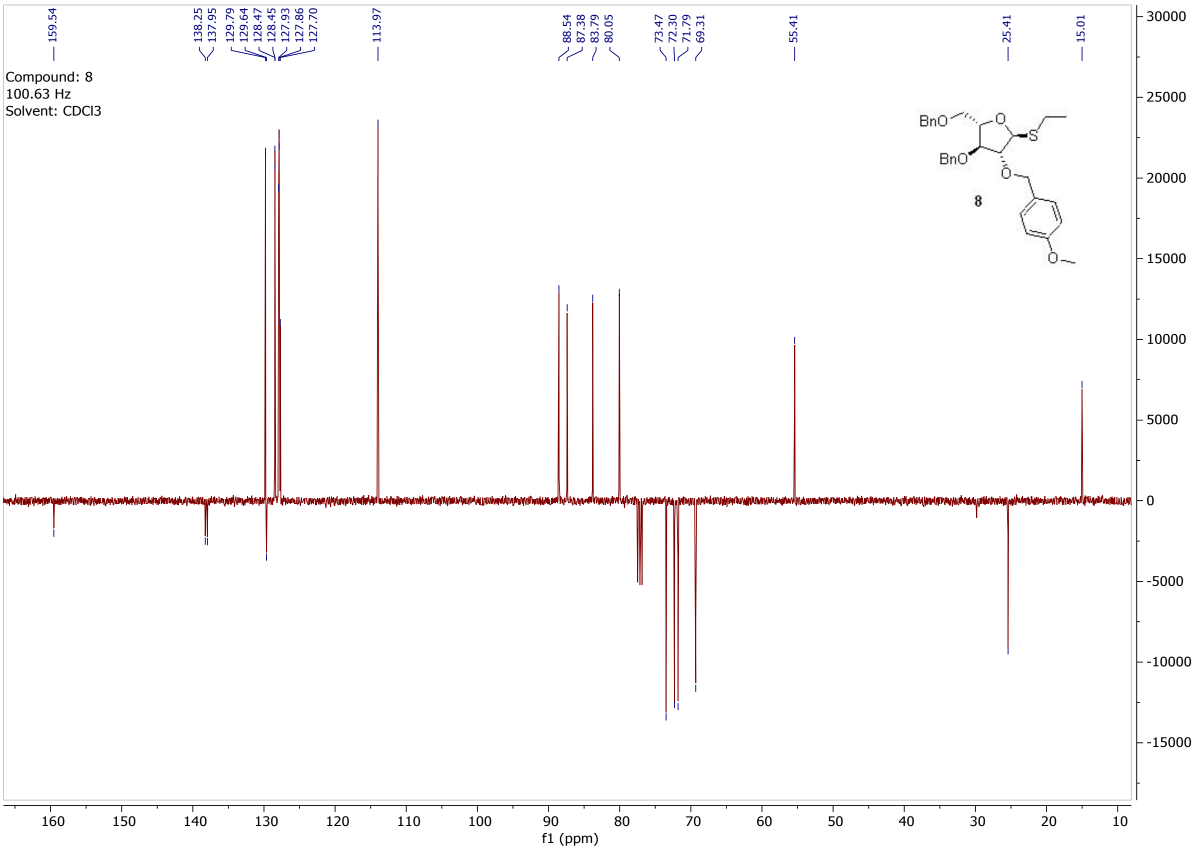

Figure S24. ^{13}C APT spectrum of ethyl 2-O-PMB-3,5-O-benzyl-1-thio-α-L-arabinofuranoside (**8**).

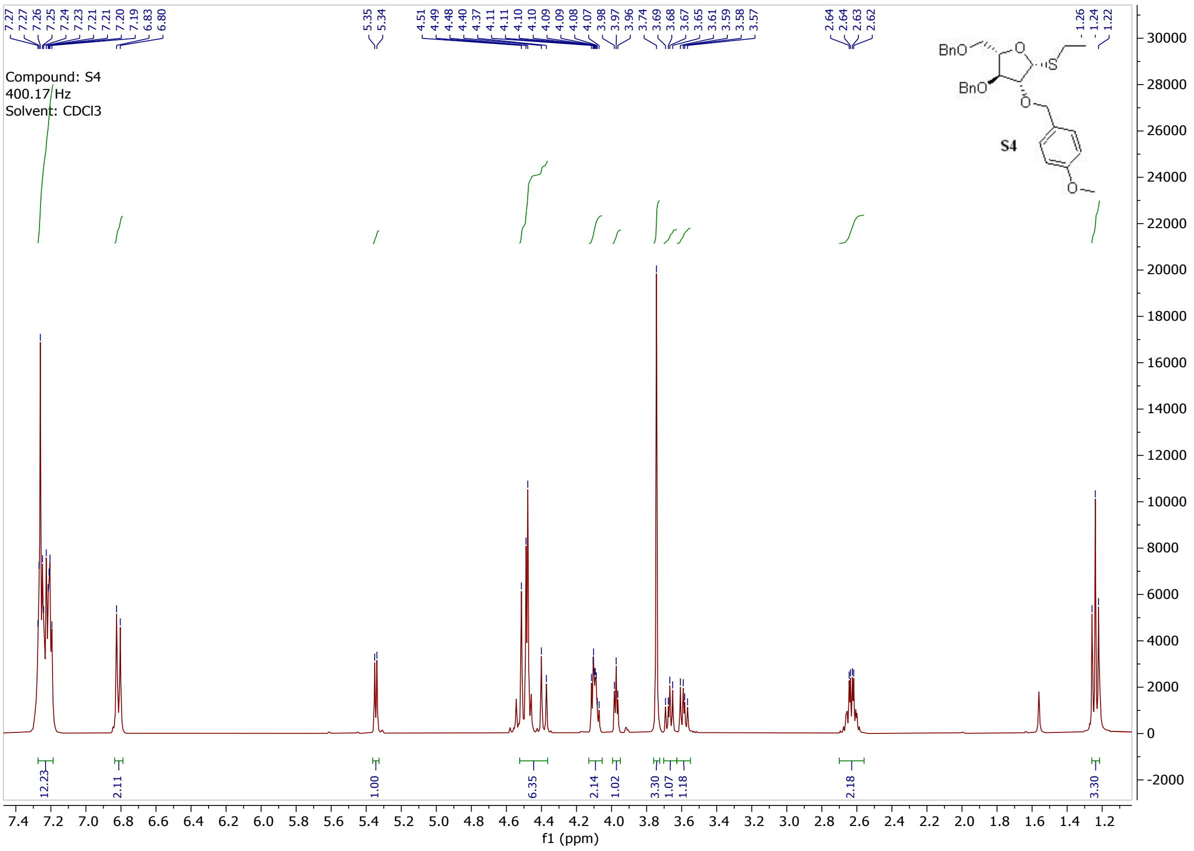

Figure S25. ^{1}H spectrum of ethyl 2-O-PMB-3,5-O-benzyl-1-thio-β-L-arabinofuranoside (**S4**).

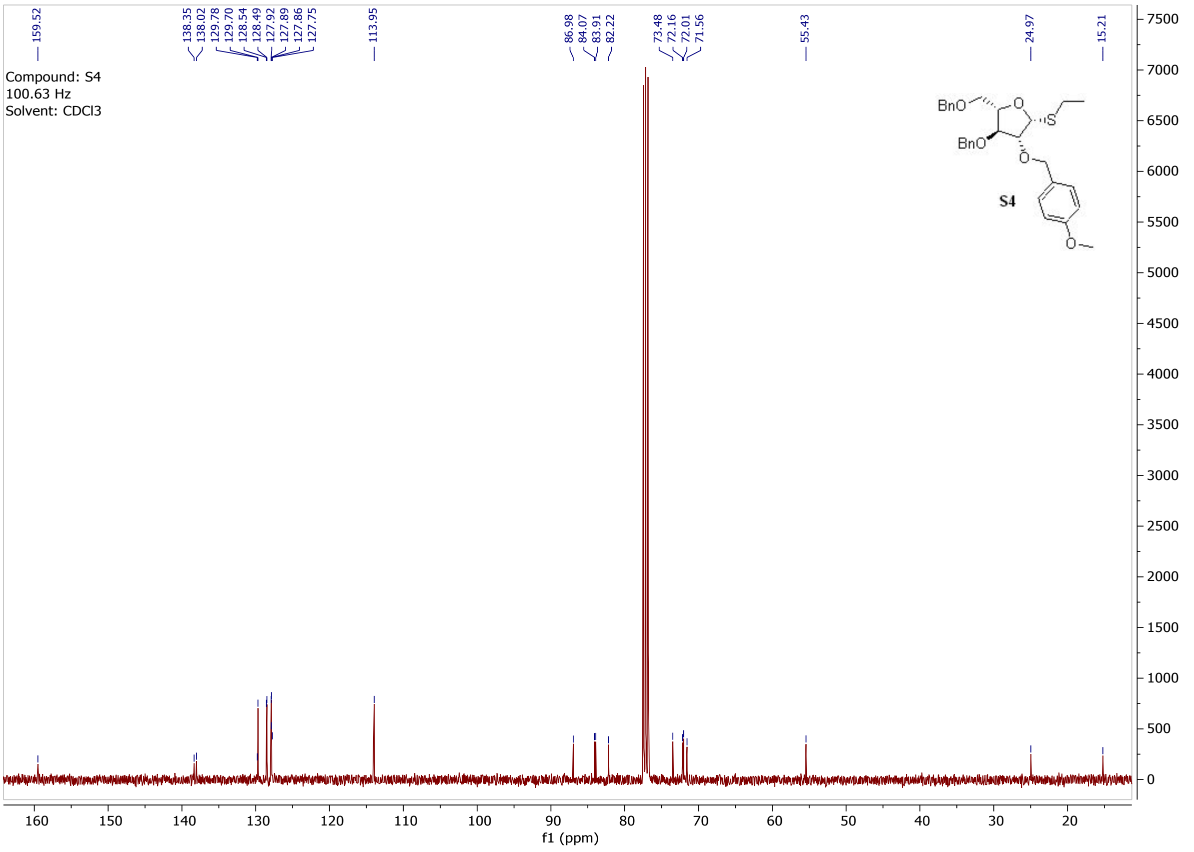

Figure S26. ^{13}C spectrum of ethyl 2-O-PMB-3,5-O-benzyl-1-thio-β-L-arabinofuranoside (**S4**).

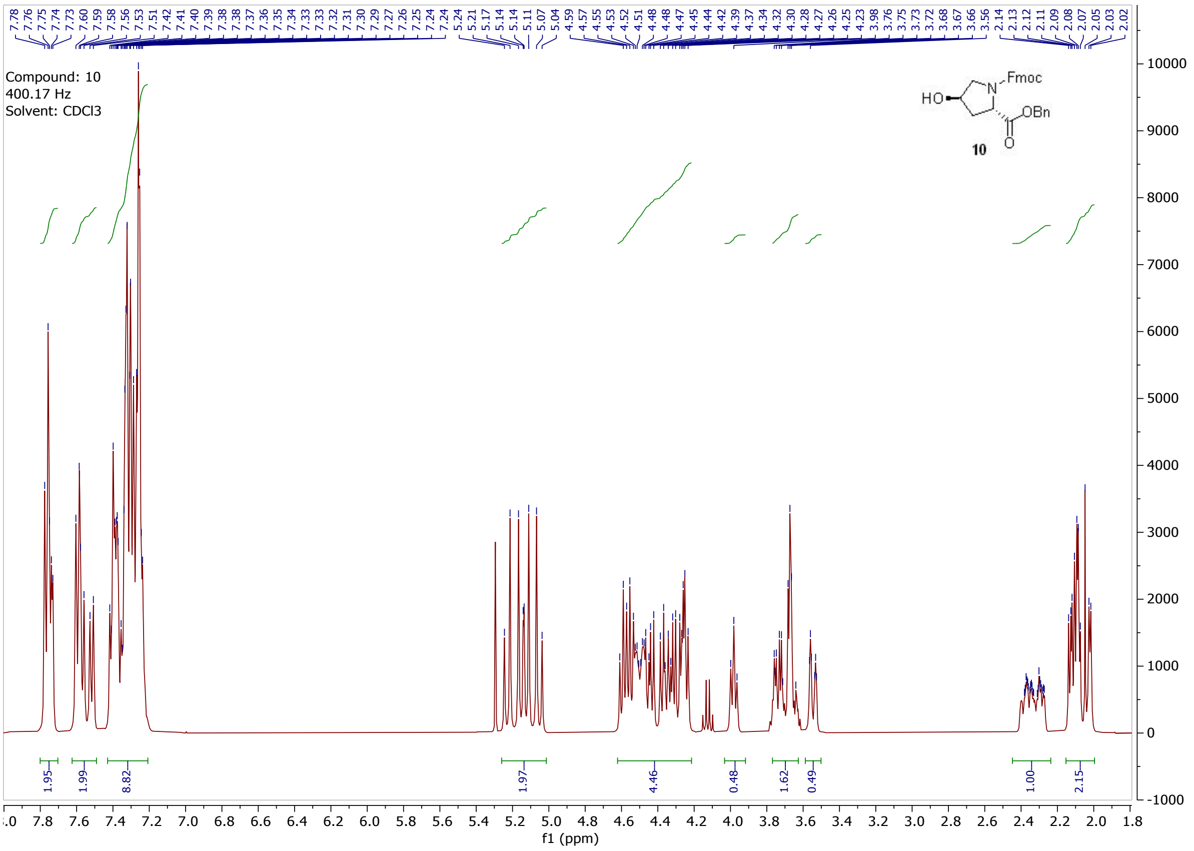

Figure S27. ^{1}H spectrum of Fmoc-Hyp-OBn (**10**).

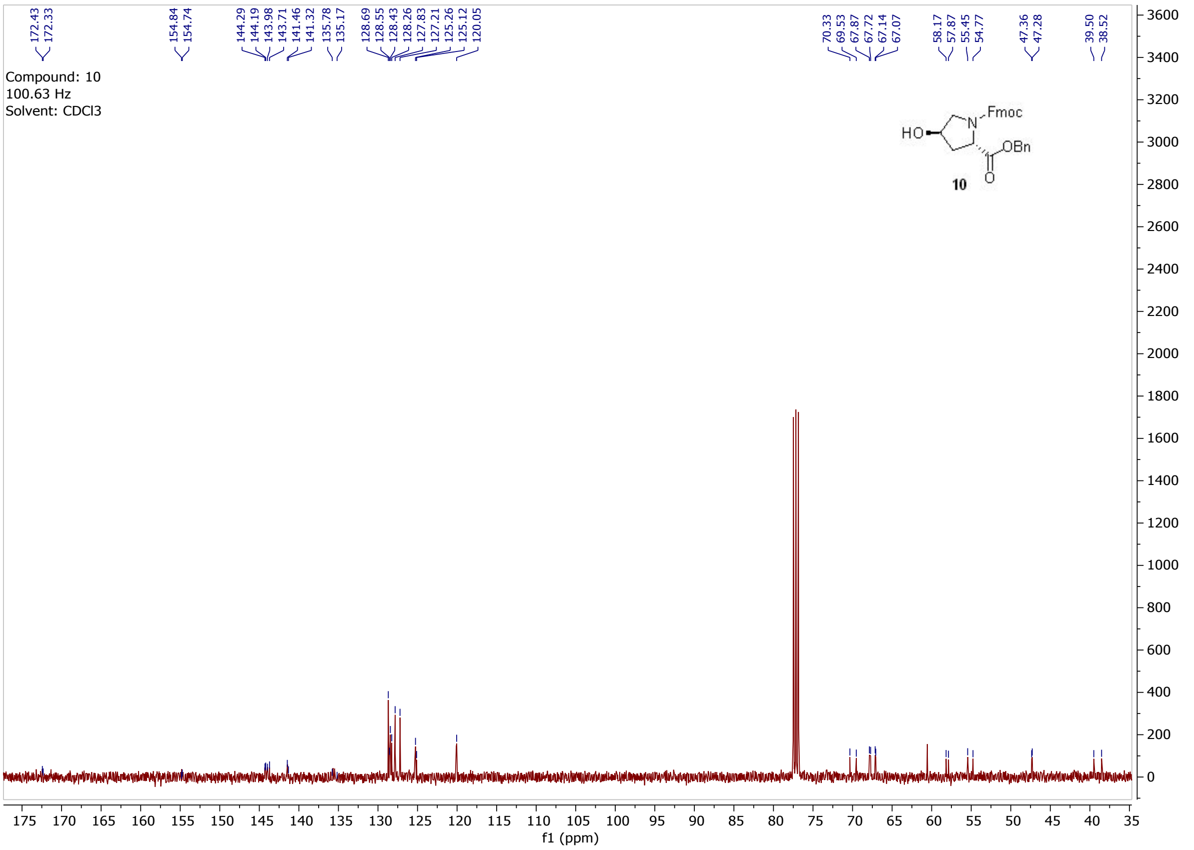

Figure S28. ^{13}C spectrum of Fmoc-Hyp-OBn (**10**).

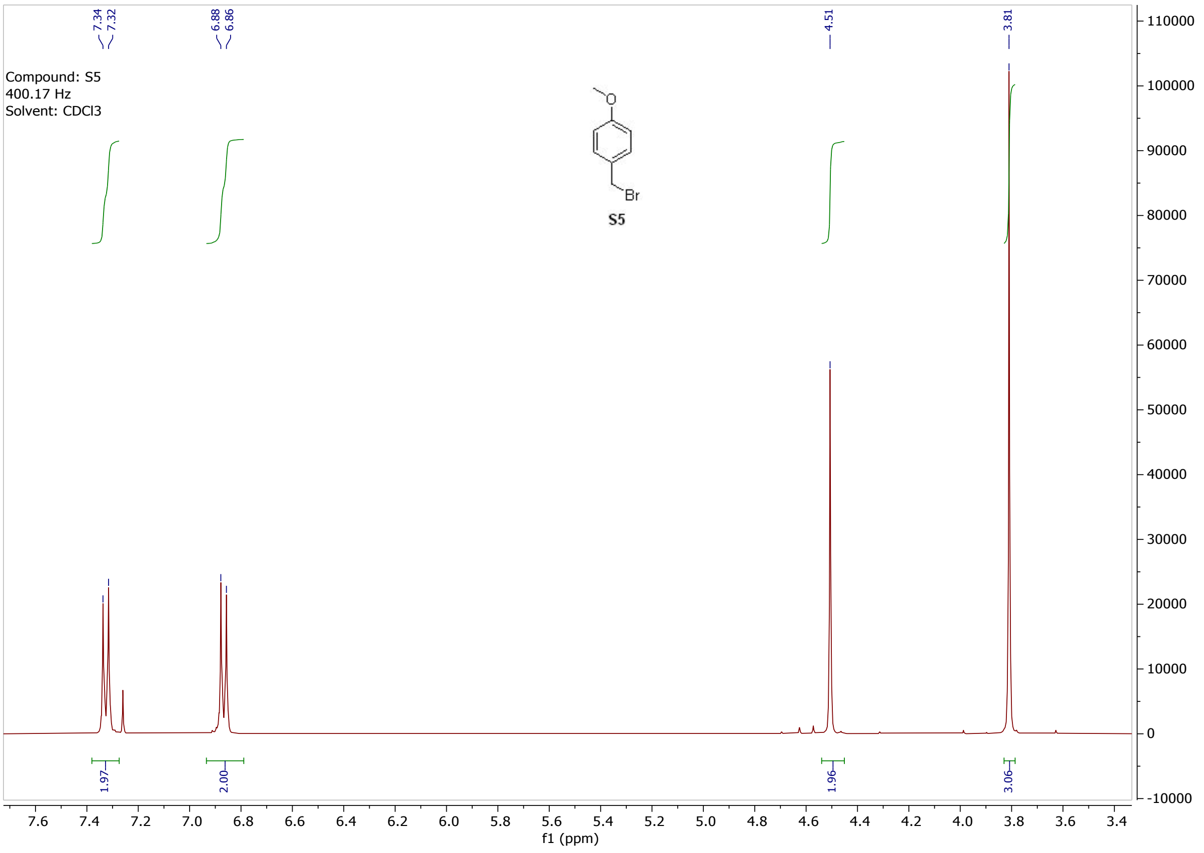

Figure S29. ^{1}H spectrum of 1-(bromomethyl)-4-methoxybenzene (**S5**).

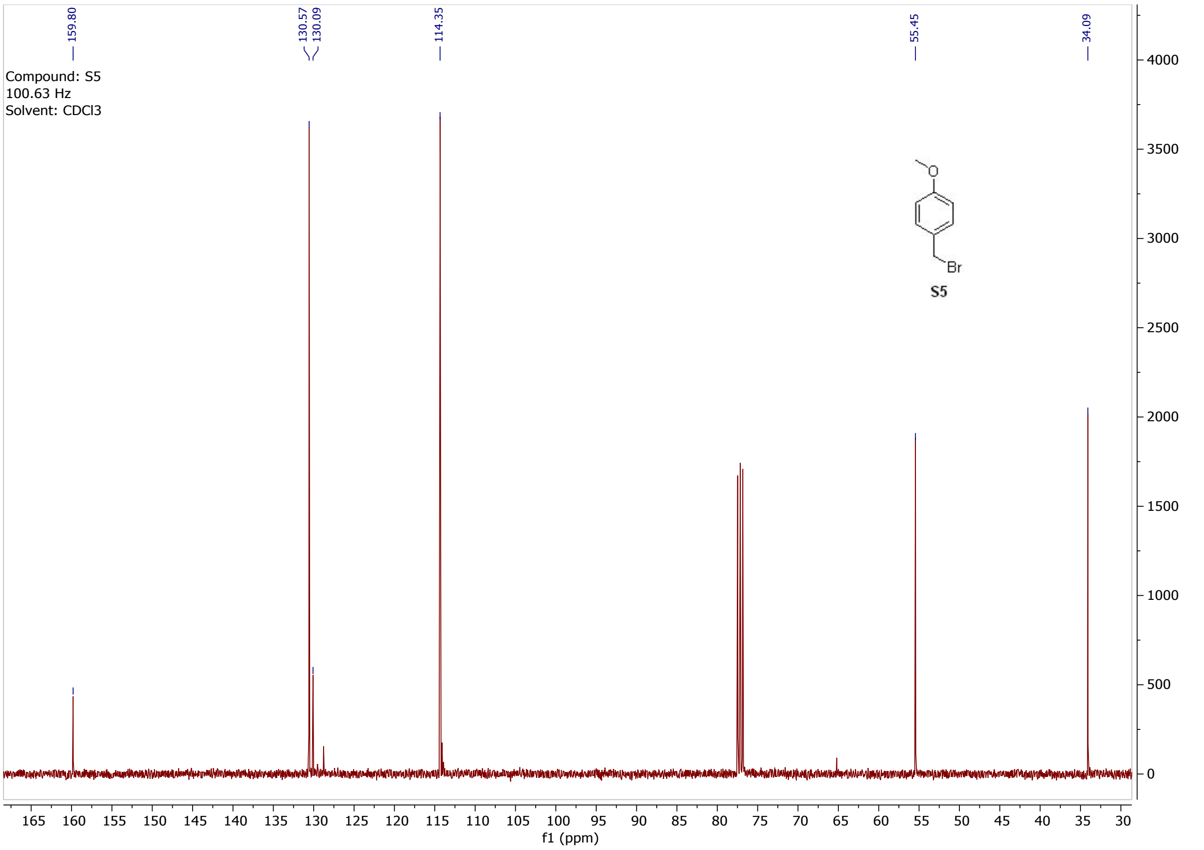

Figure S30. ^{13}C spectrum of 1-(bromomethyl)-4-methoxybenzene (**S5**).

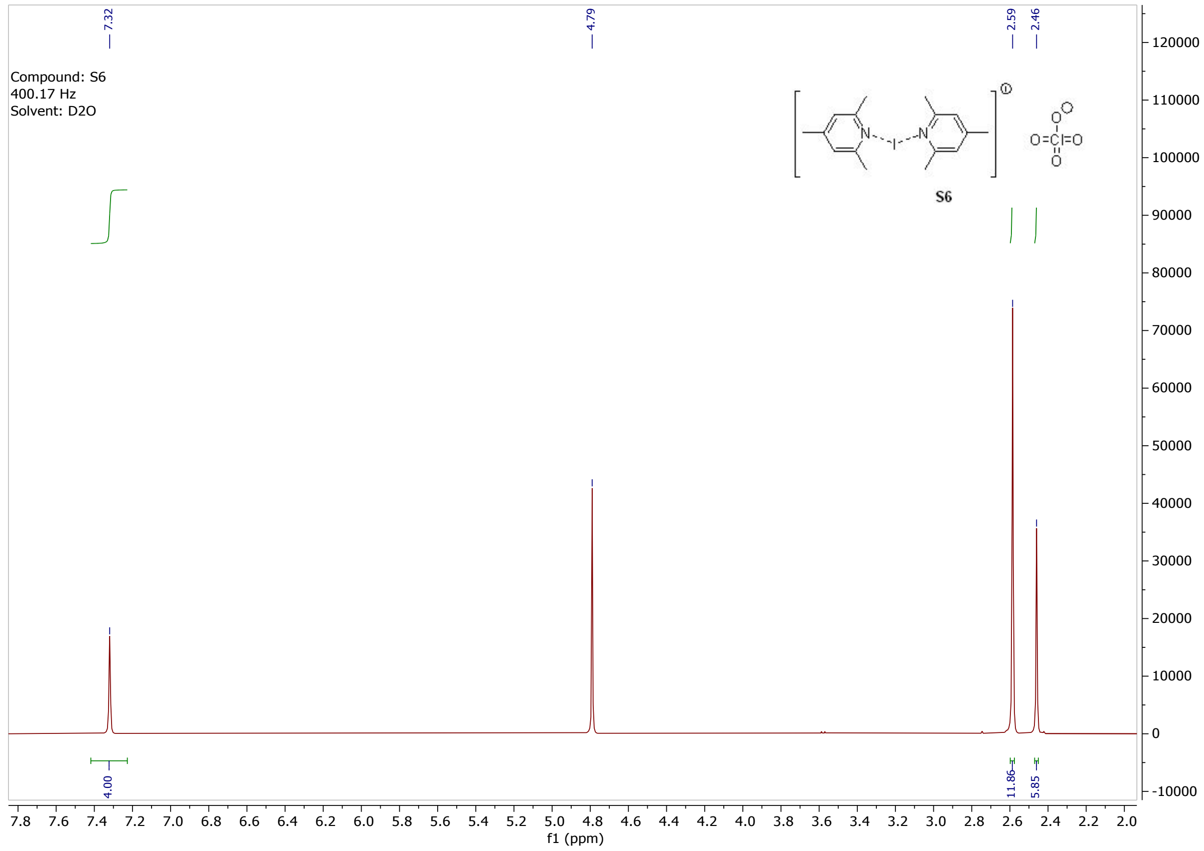

Figure S31. ^{1}H spectrum of iodonium di-collidine perchlorate (IDCP) (**S6**).

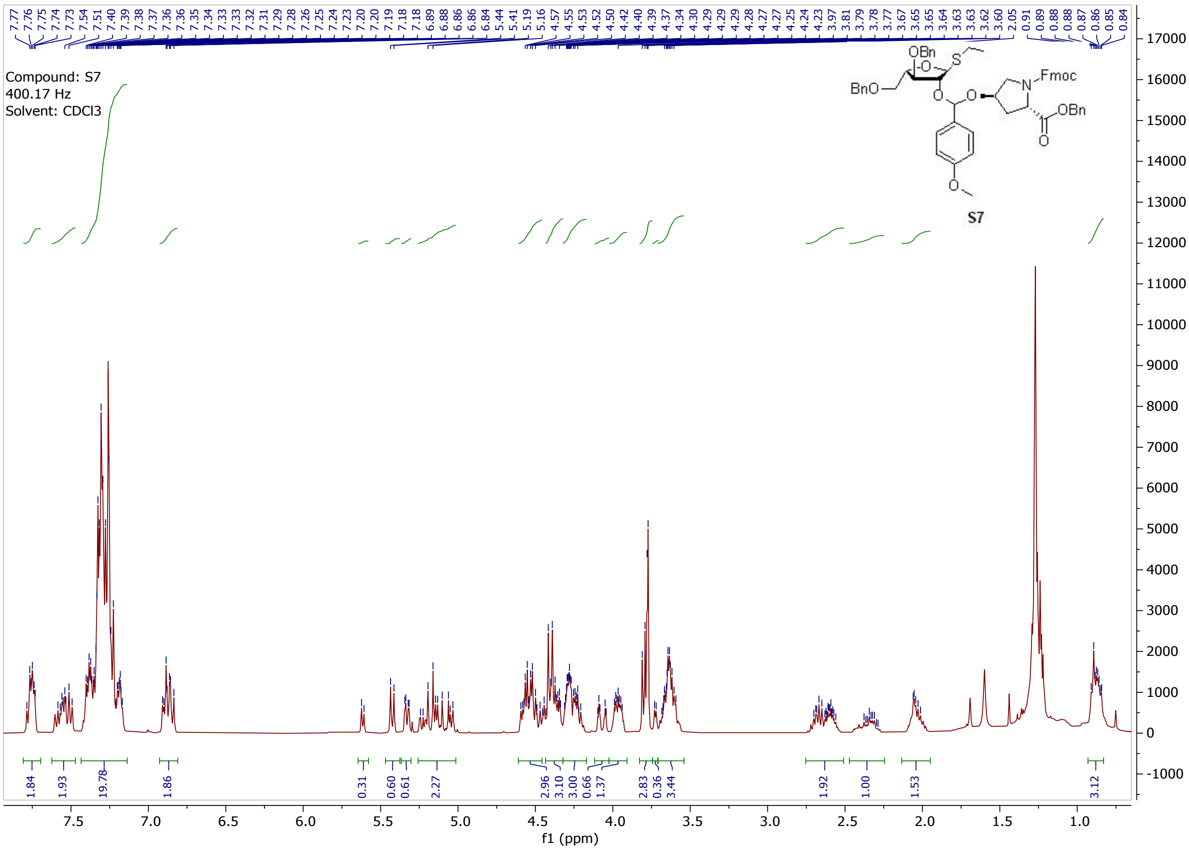

Figure S32. ^{1}H spectrum of Fmoc-[(3,5-Bn)Ara]$_1$-2-PMB acetal Hyp-OBn (**S7**).

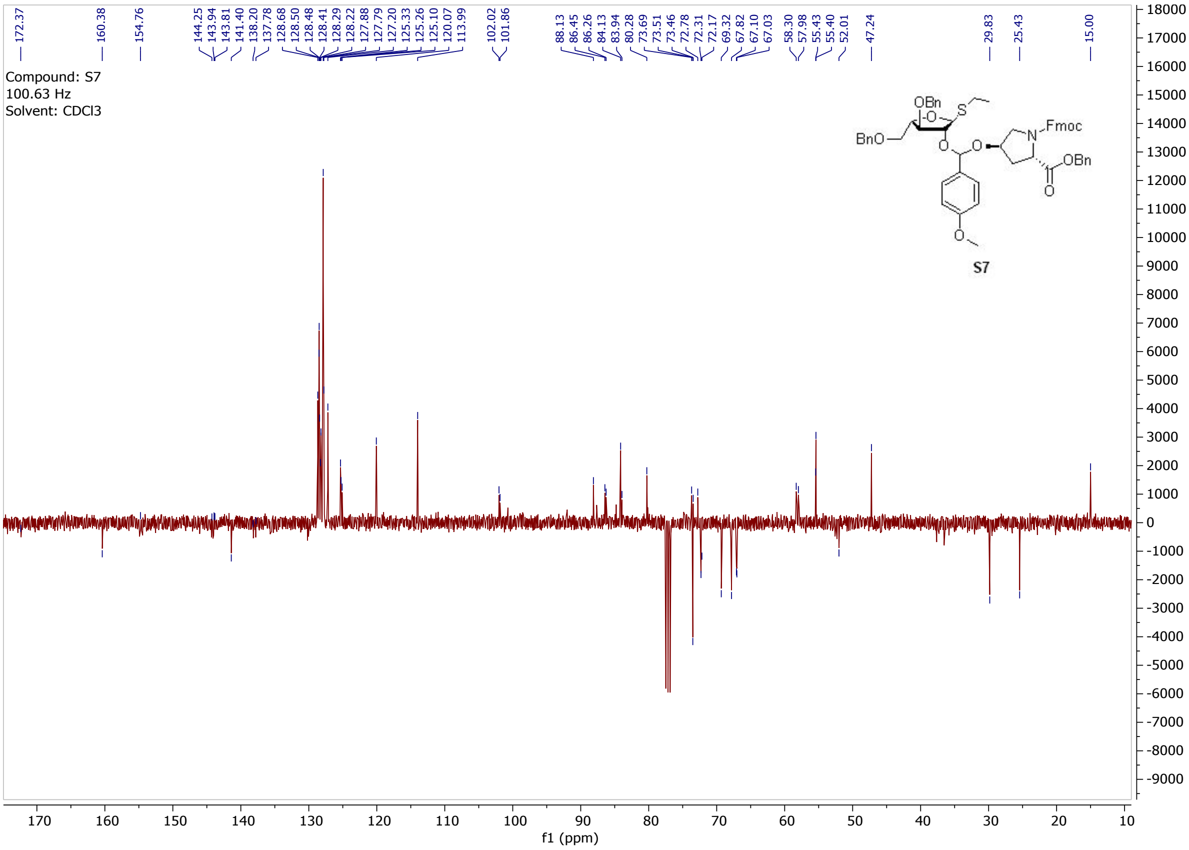

Figure S33. ^{13}C APT spectrum of Fmoc-[(3,5-Bn)Ara]$_1$-2-PMB acetal Hyp-OBn (**S7**).

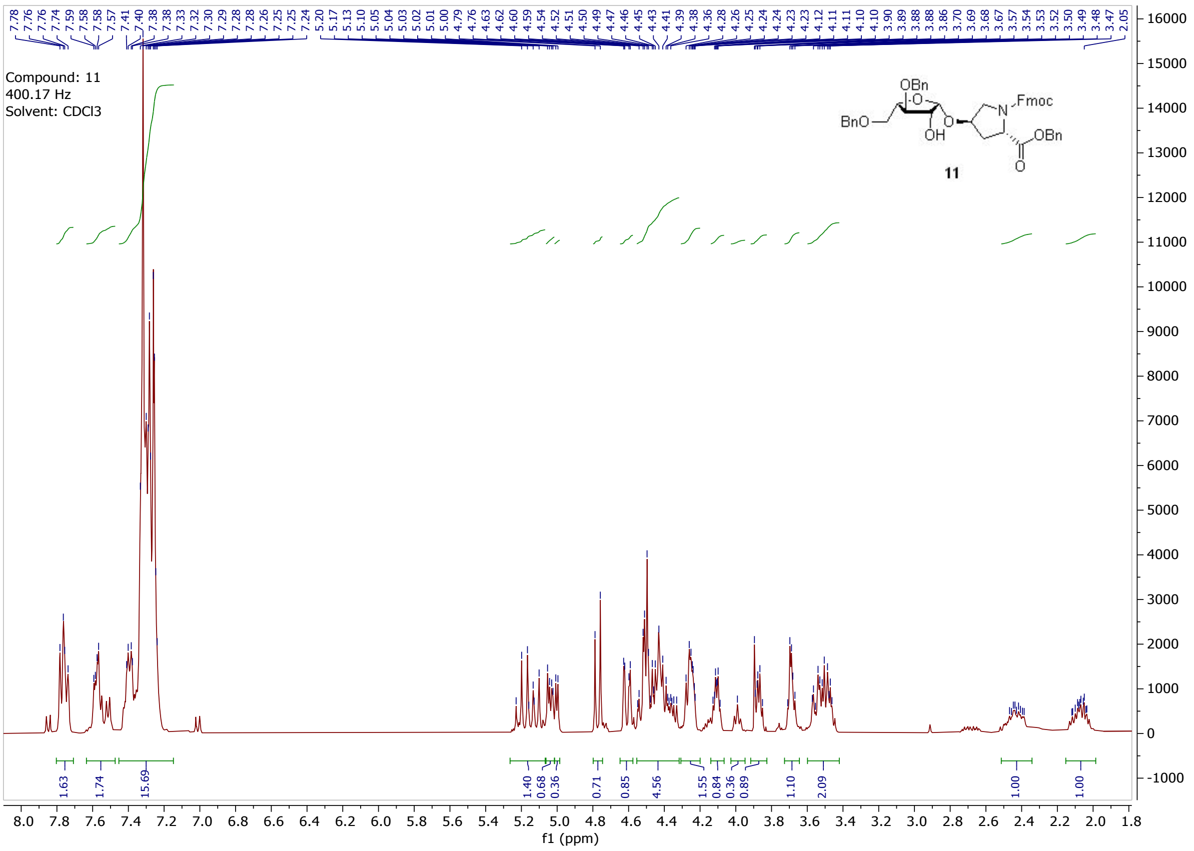

Figure S34. ^{1}H spectrum of Fmoc-[(3,5-Bn)Ara]$_1$Hyp-OBn (**11**).

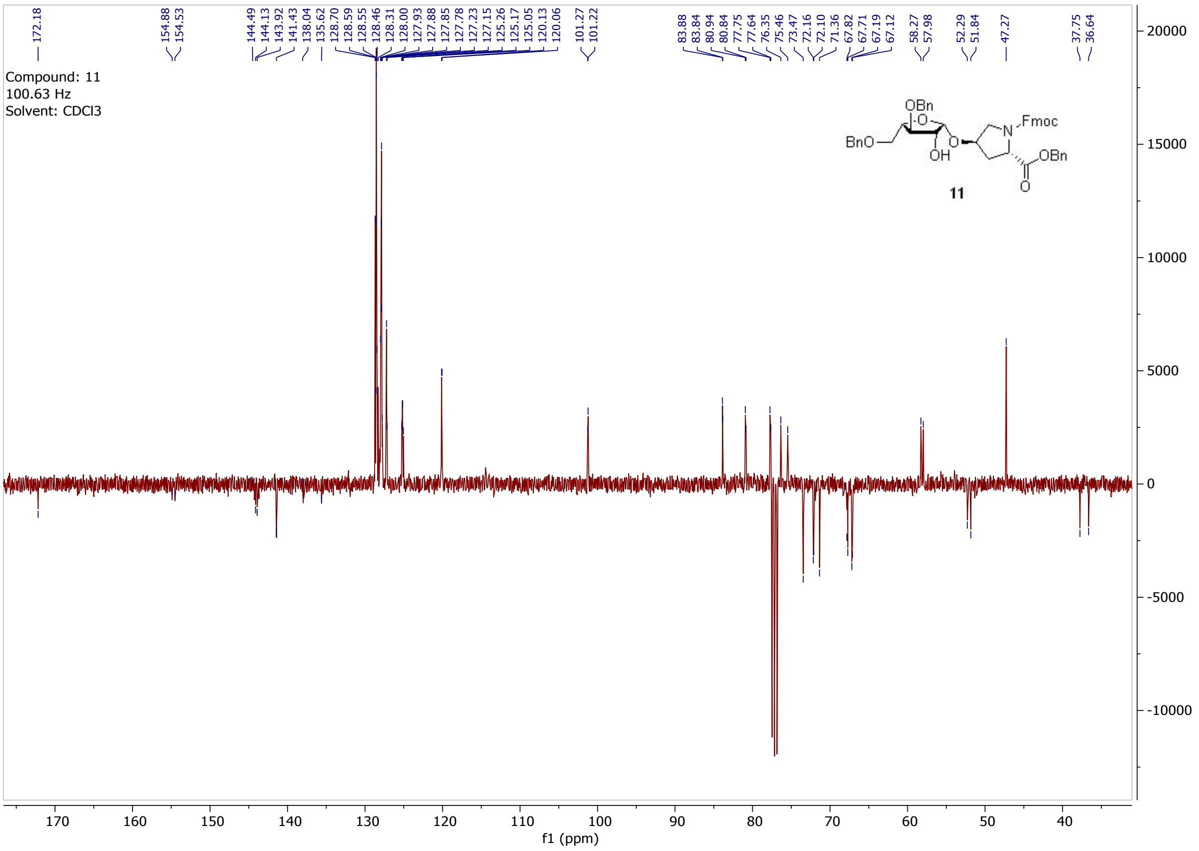

Figure S35. ^{13}C APT spectrum of Fmoc-[(3,5-Bn)Ara]$_1$Hyp-OBn (**11**).

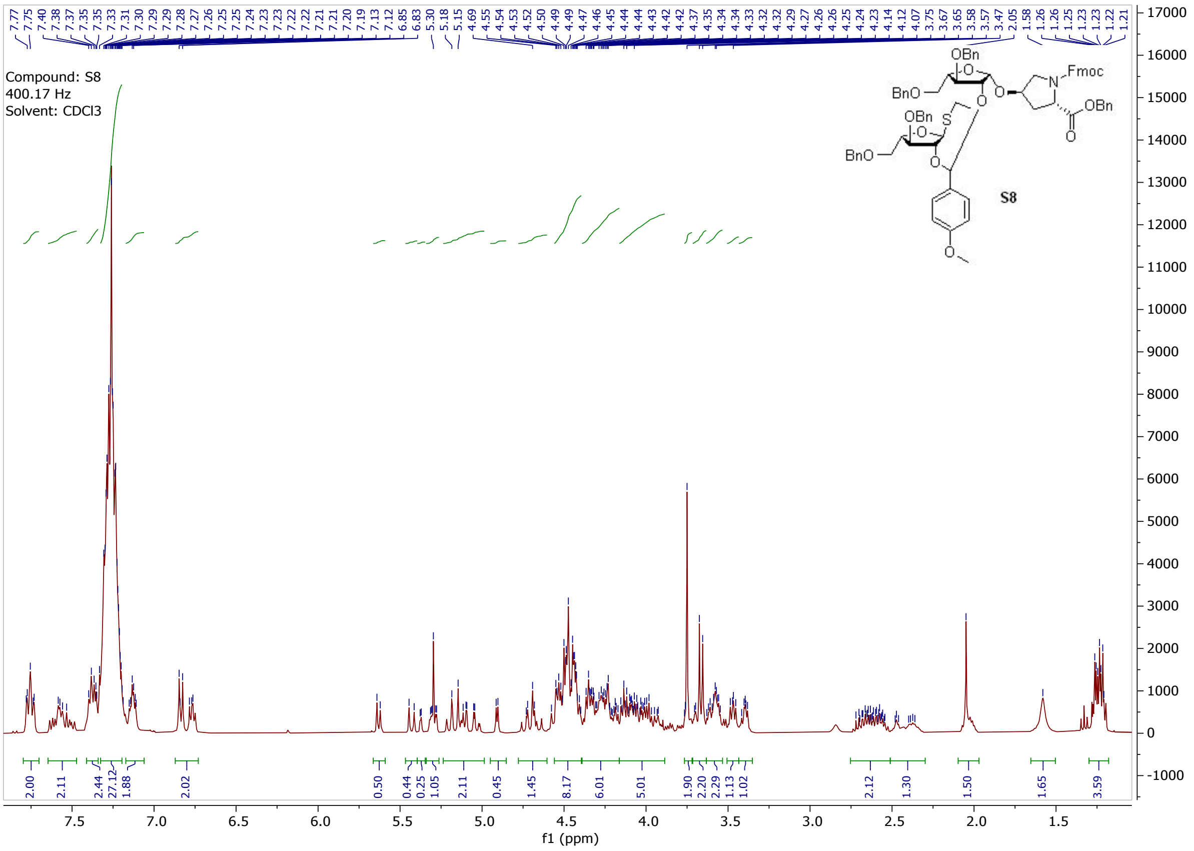

Figure S36. ^{1}H spectrum of Fmoc-[(3,5-Bn)Ara]$_2$-2-PMB acetal Hyp-OBn (**S8**).

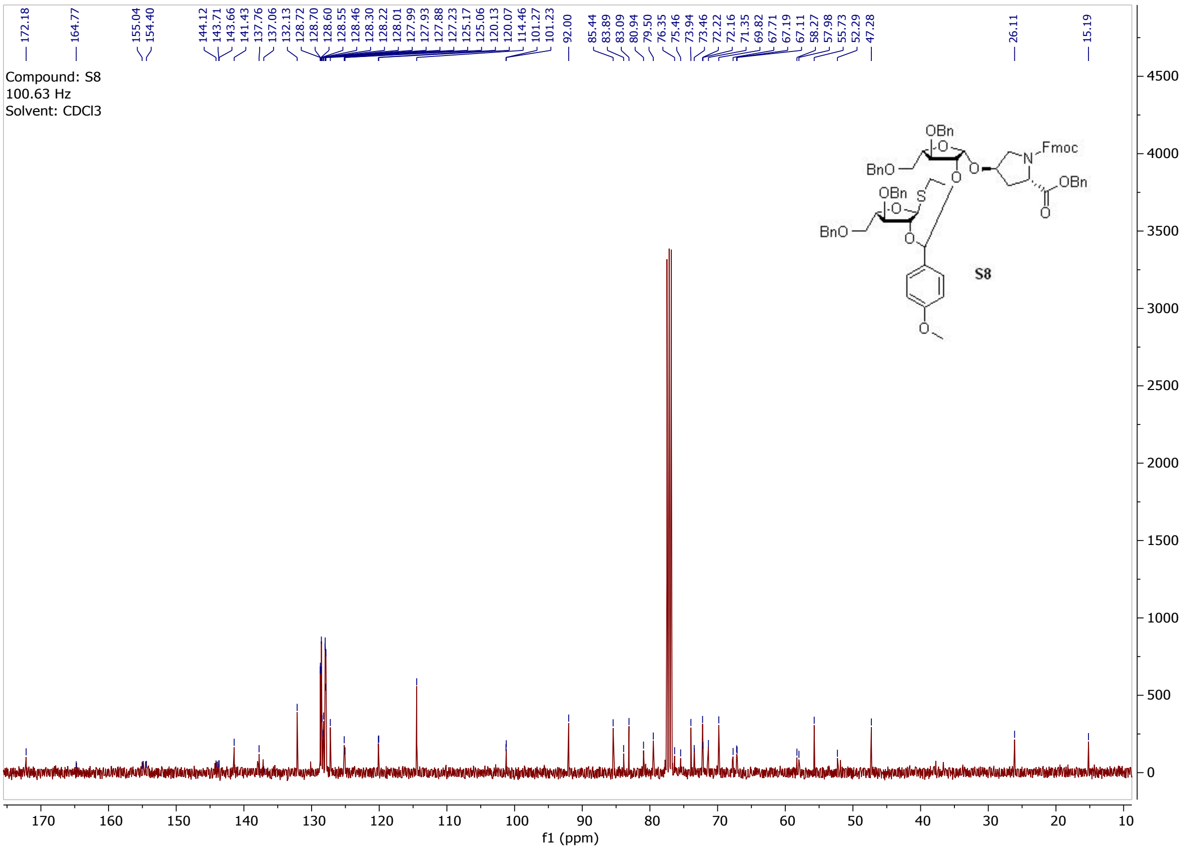

Figure S37. ^{13}C spectrum of Fmoc-[(3,5-Bn)Ara]$_2$-2-PMB acetal Hyp-OBn (**S8**).

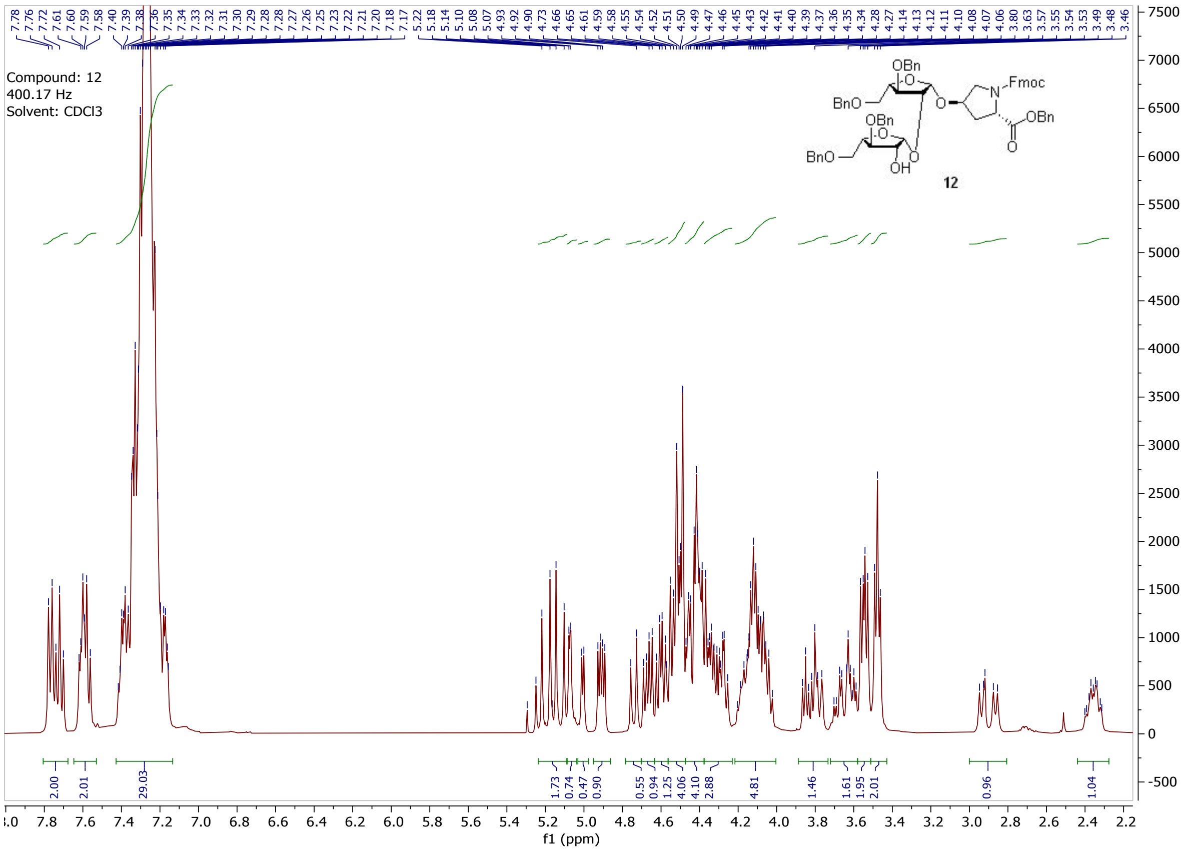

Figure S38. ^{1}H spectrum of Fmoc-[(3,5-Bn)Ara]$_2$Hyp-OBn (**12**).

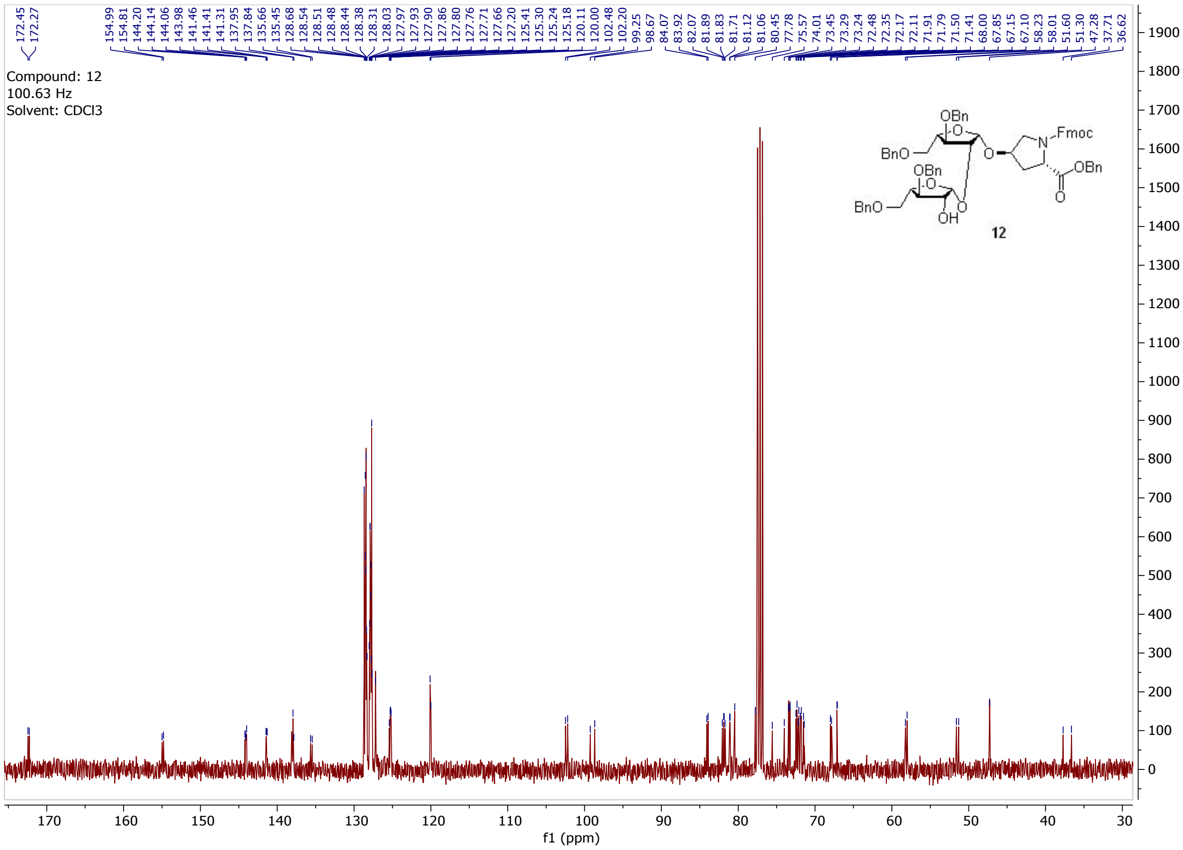

Figure S39. ^{13}C spectrum of Fmoc-[(3,5-Bn)Ara]$_2$Hyp-OBn (**12**).

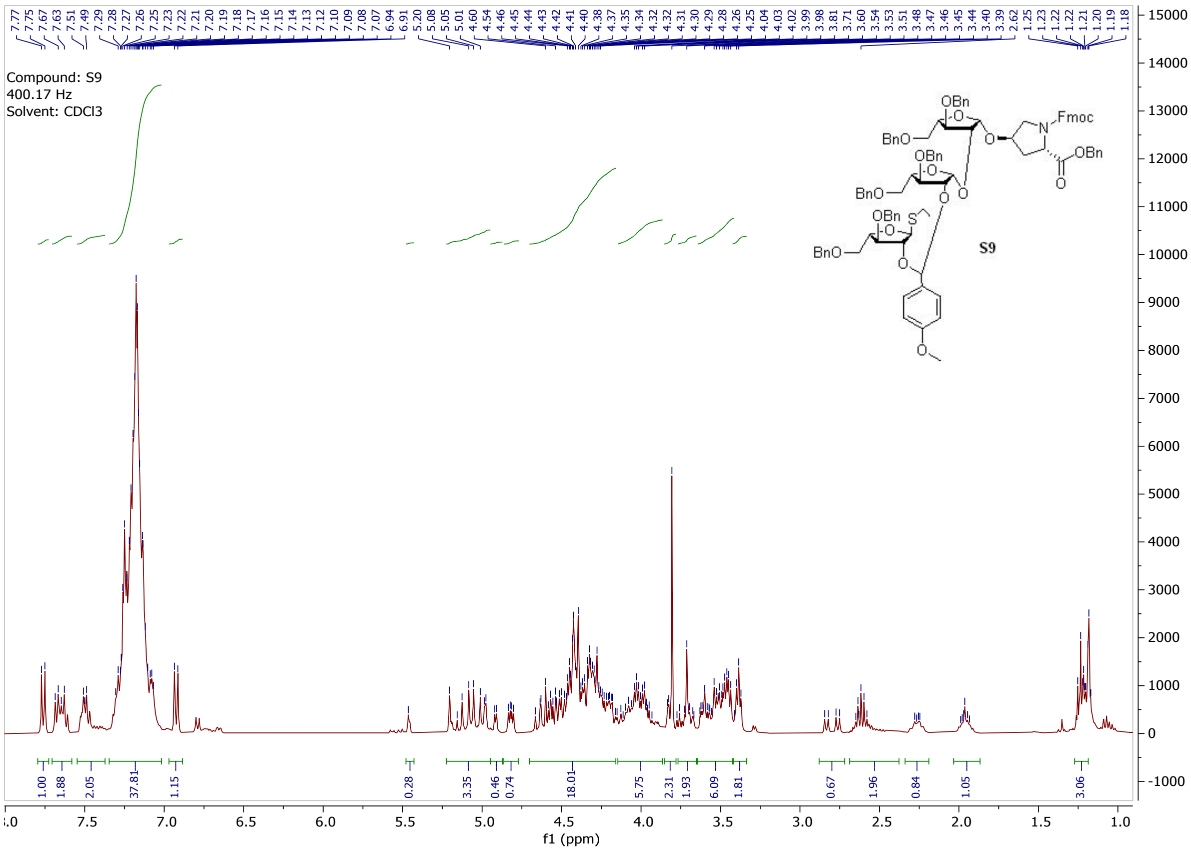

Figure S40. ^{1}H spectrum of Fmoc-[(3,5-Bn)Ara]$_3$-2-PMB acetal Hyp-OBn (**S9**).

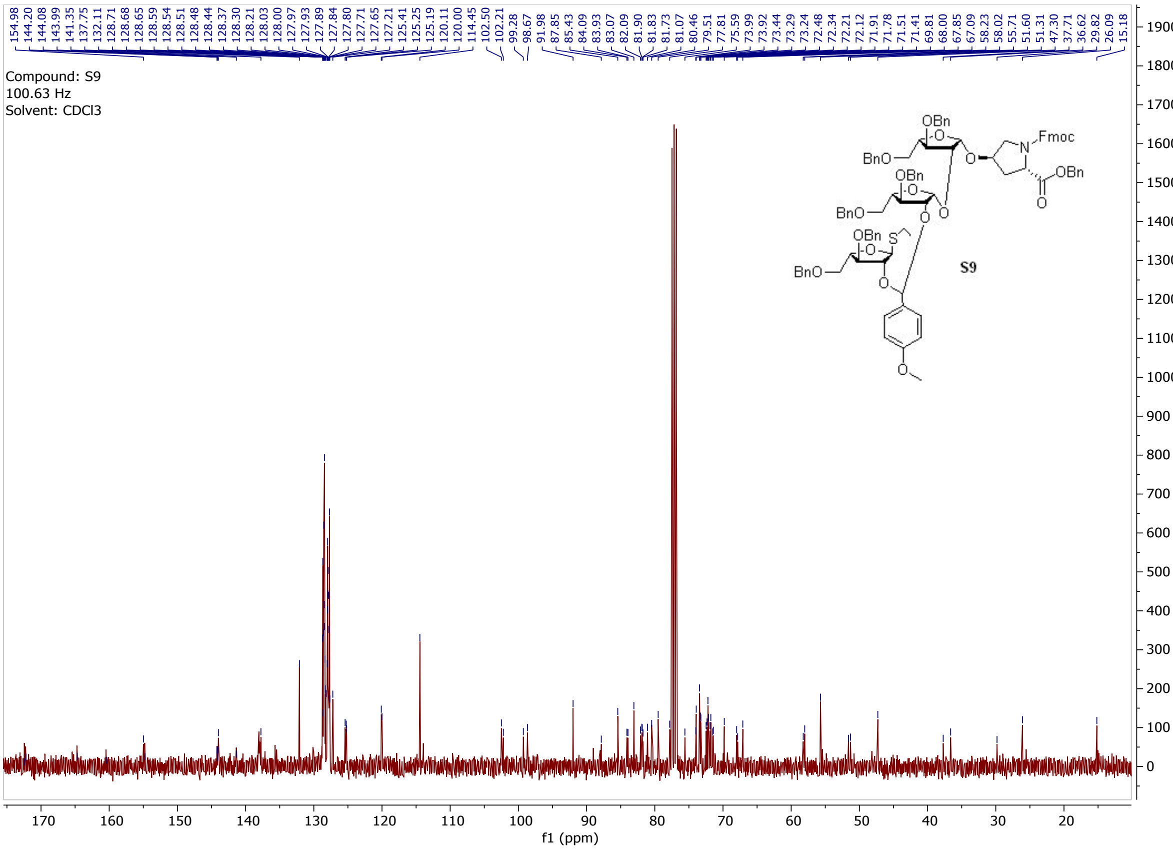

Figure S41. ^{13}C spectrum of Fmoc-[(3,5-Bn)Ara]$_3$-2-PMB acetal Hyp-OBn (**S9**).

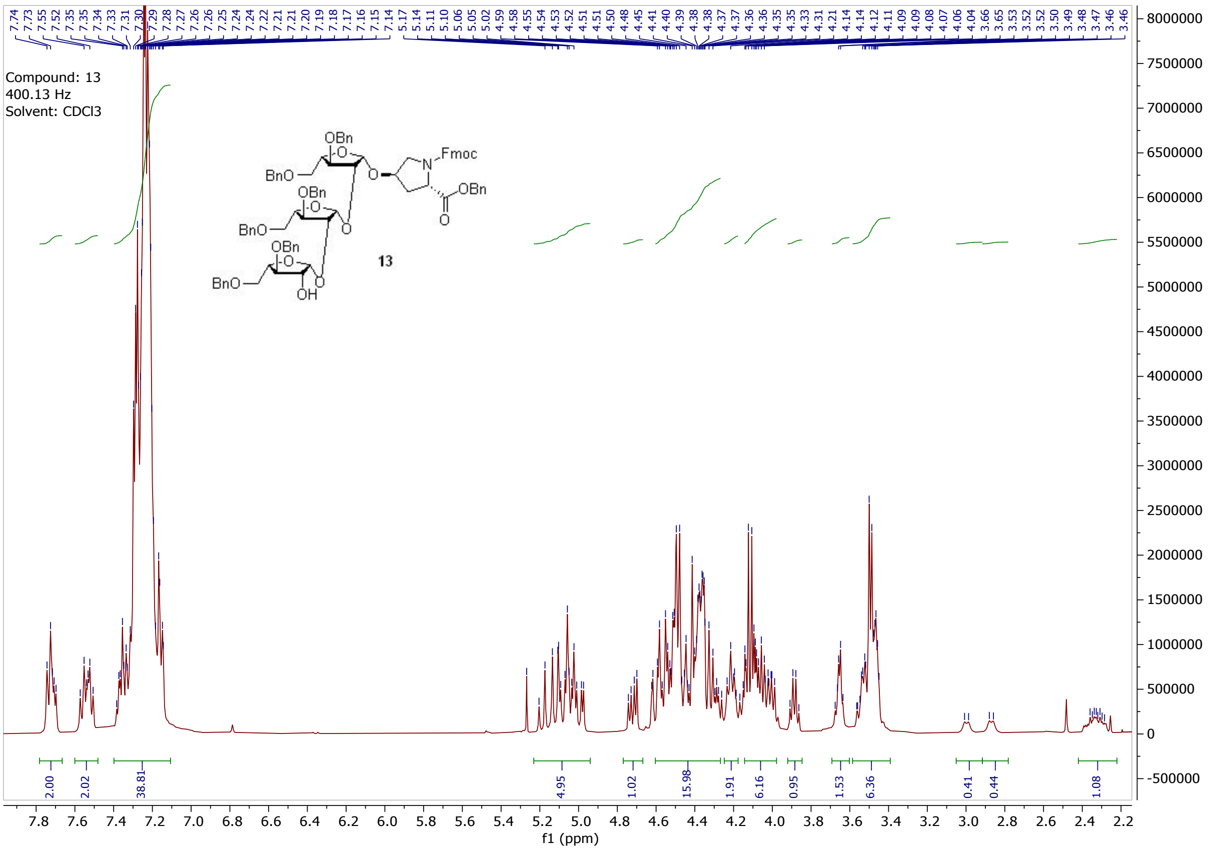

Figure S42. ^{1}H spectrum of Fmoc-[(3,5-Bn)Ara]$_3$Hyp-OBn (**13**).

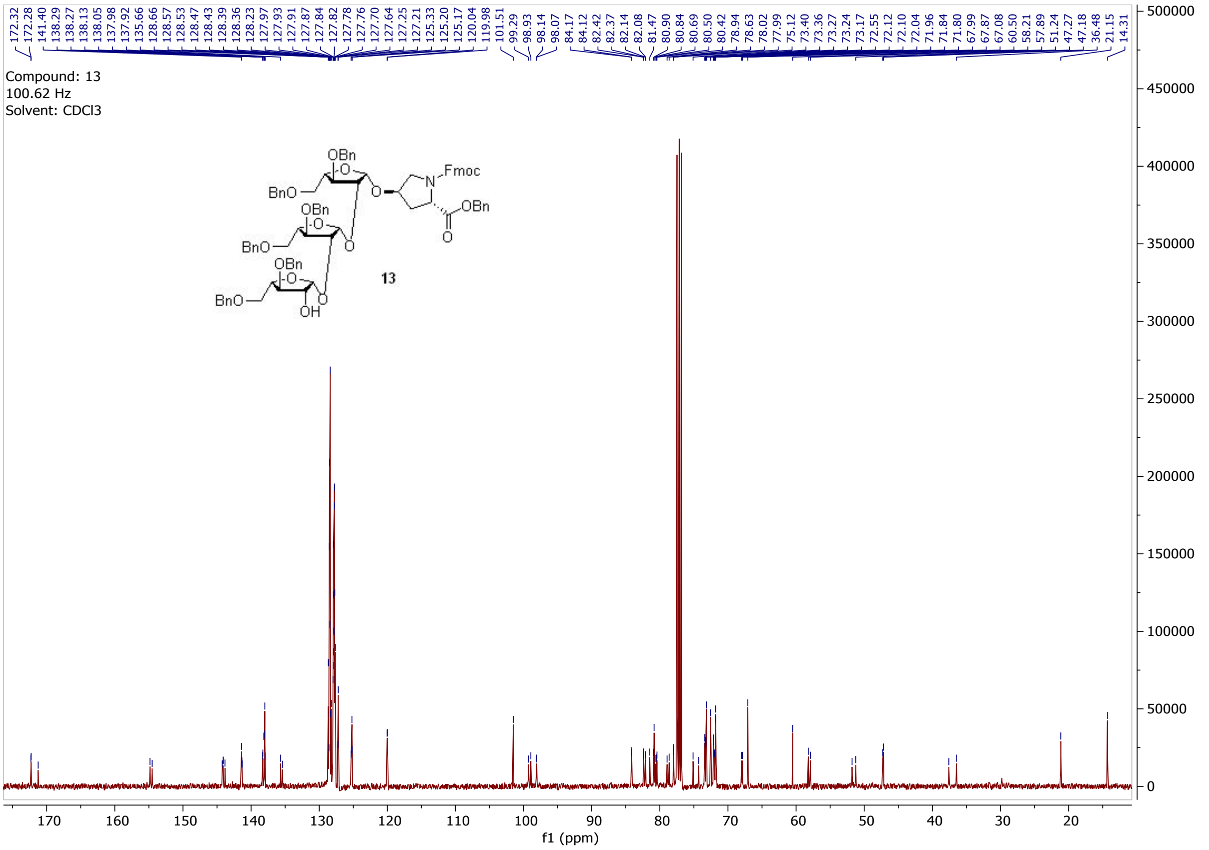

Figure S43. ^{13}C spectrum of Fmoc-[(3,5-Bn)Ara]$_3$Hyp-OBn (**13**).

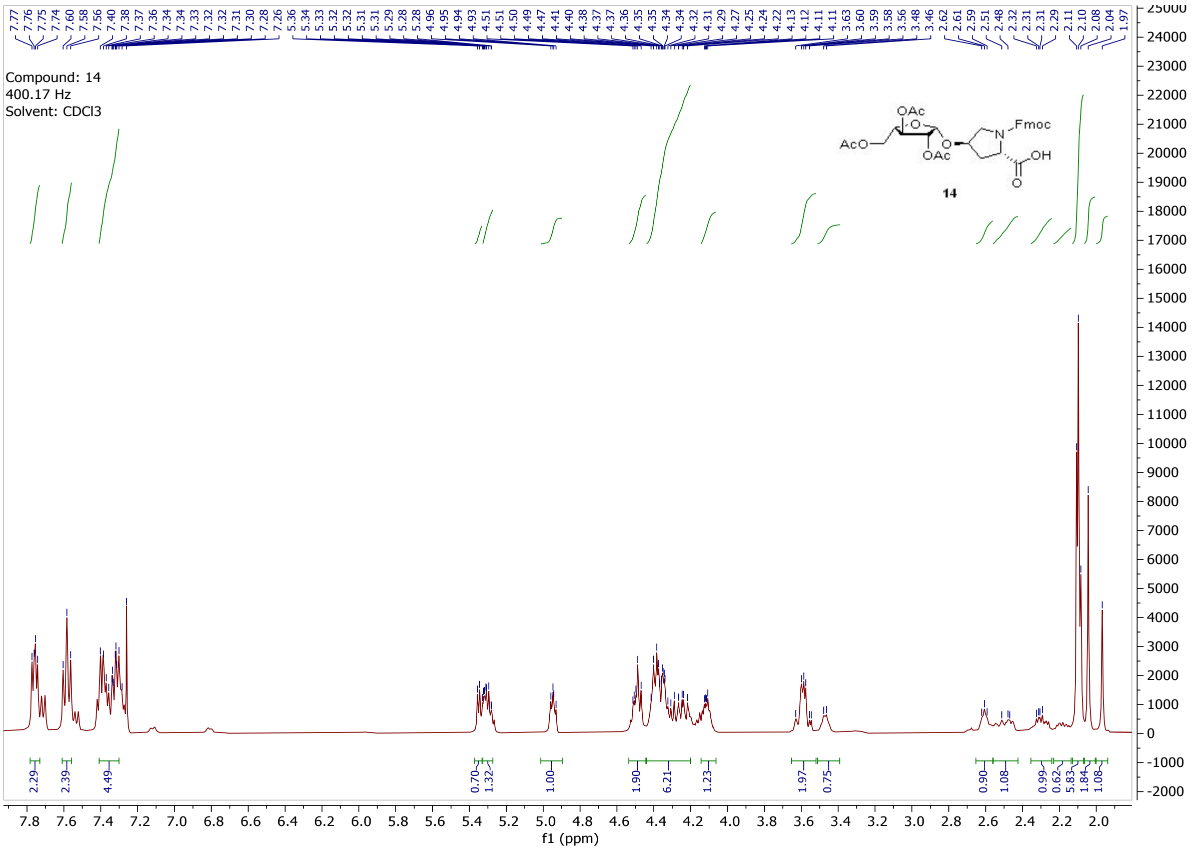

Figure S44. ^{1}H spectrum of Fmoc-[Ara(OAc)]$_1$Hyp-OBn (**14**).

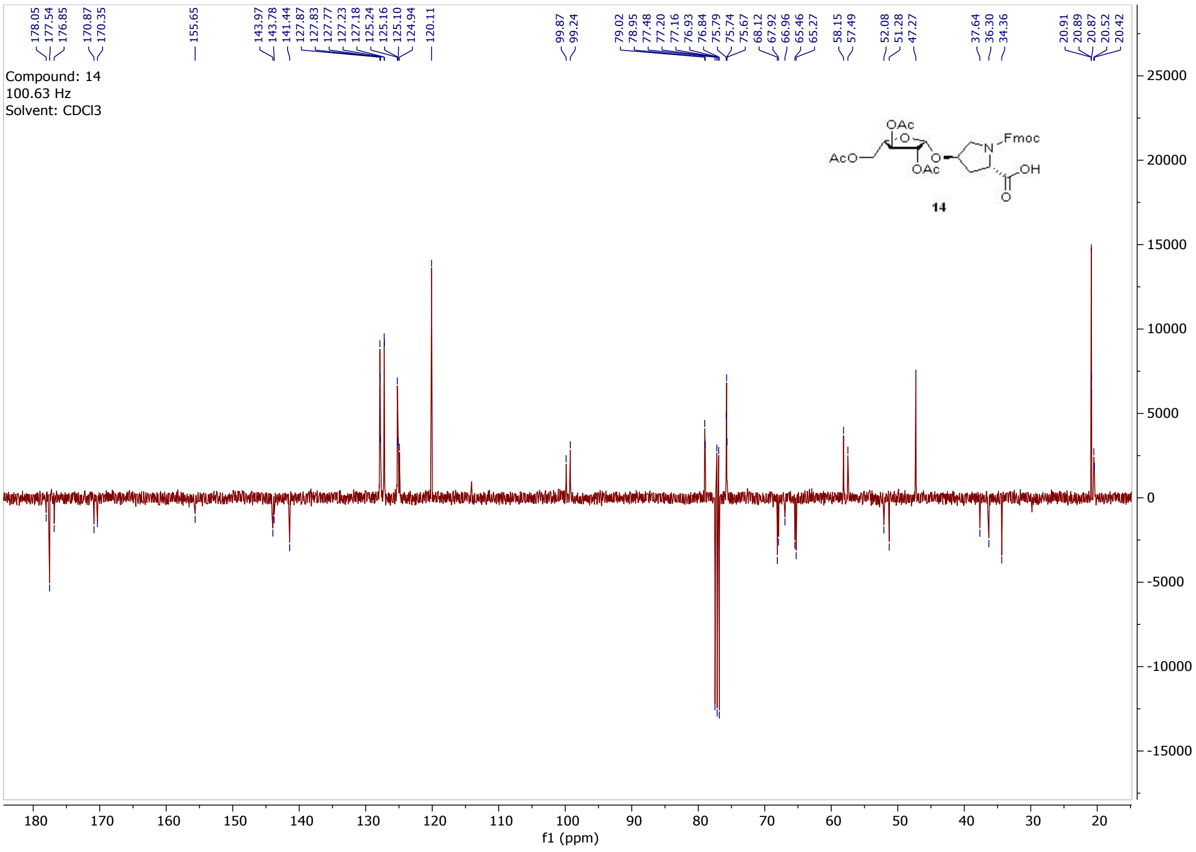

Figure S45. ^{13}C APT spectrum of Fmoc-[Ara(OAc)]$_1$Hyp-OBn (**14**).

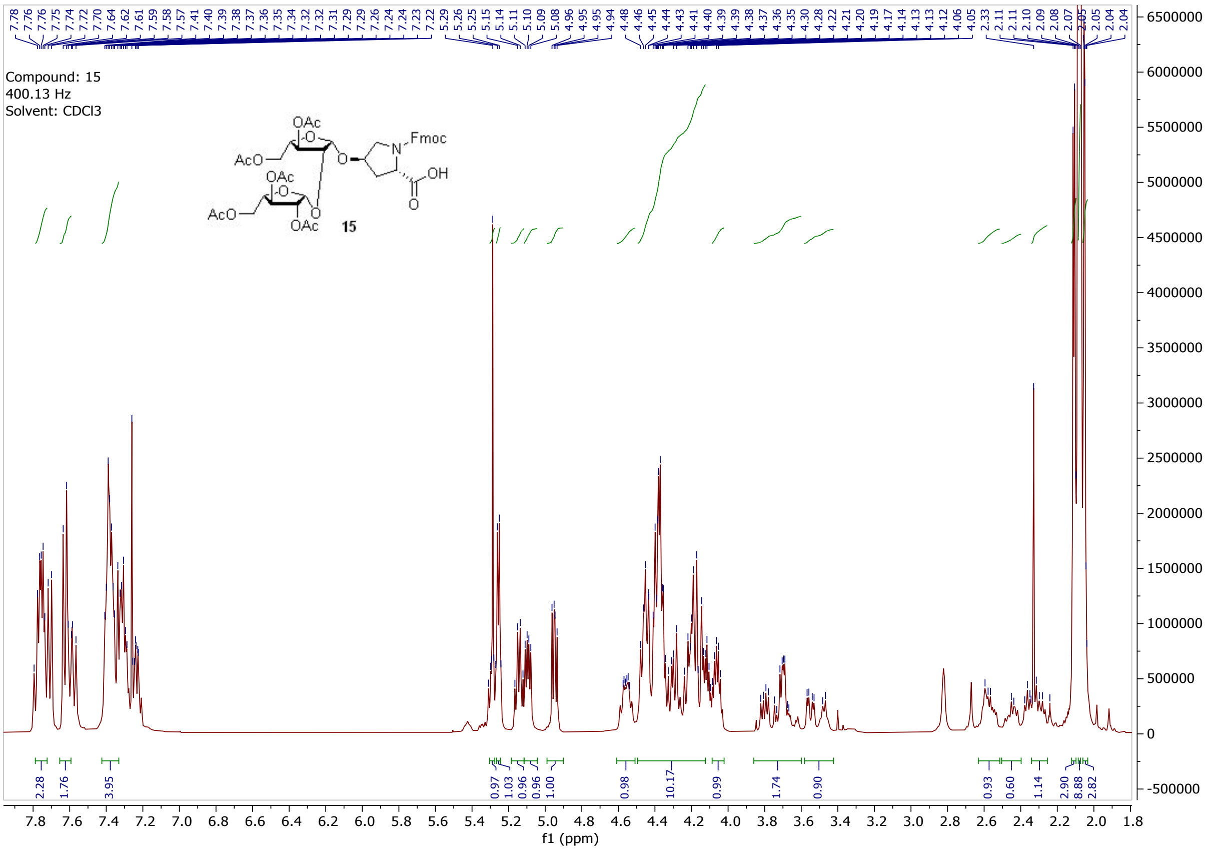

Figure S46. ^{1}H spectrum of Fmoc-[Ara(OAc)]$_2$Hyp-OBn (**15**).

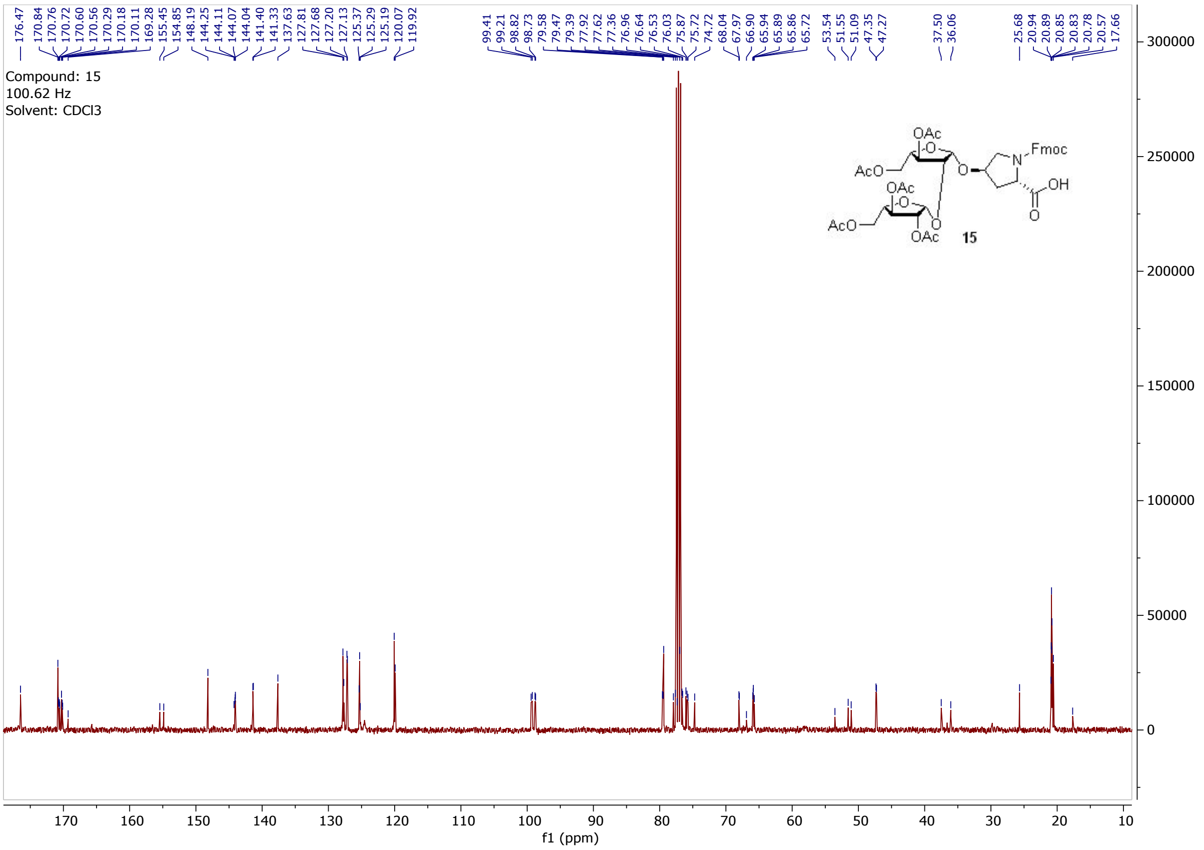

Figure S47. ^{13}C spectrum of Fmoc-[Ara(OAc)]$_2$Hyp-OBn (**15**).

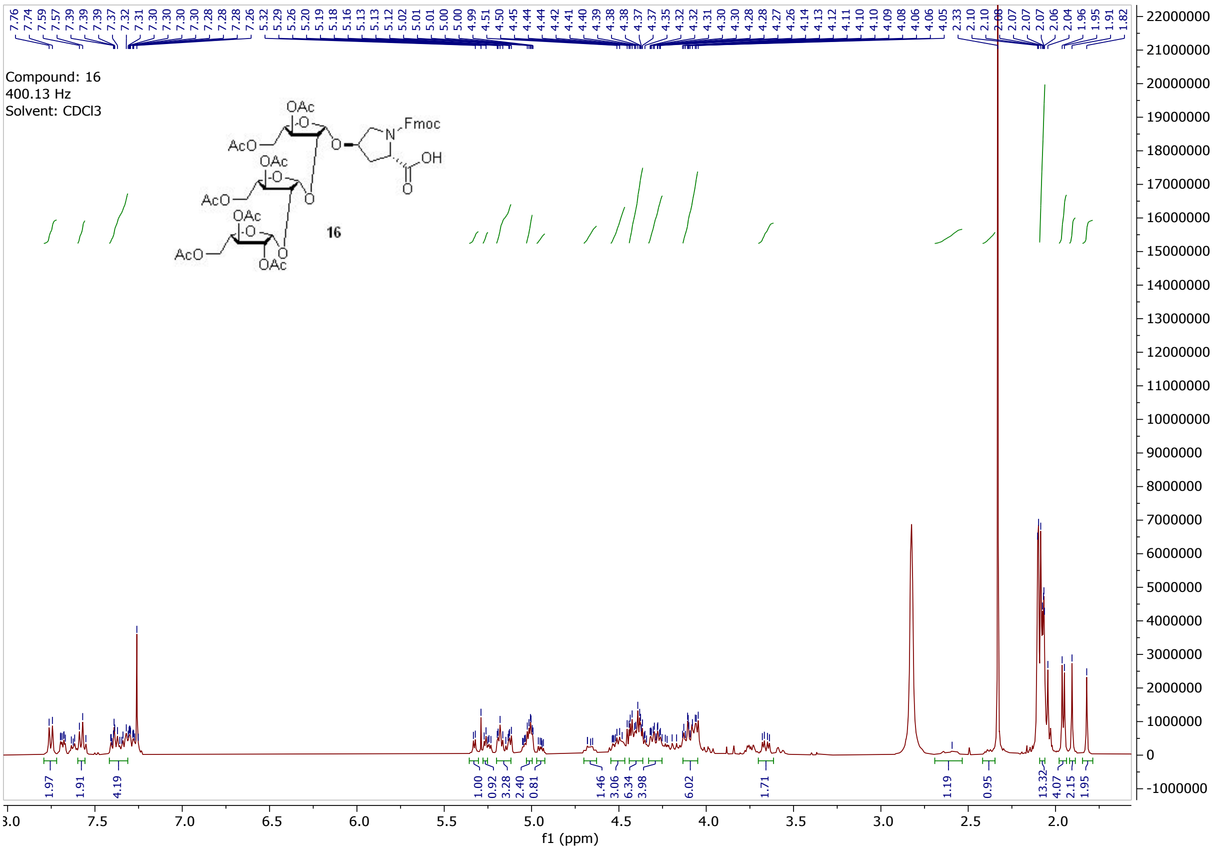

Figure S48. ^{1}H spectrum of Fmoc-[Ara(OAc)]$_3$Hyp-OBn (**16**).

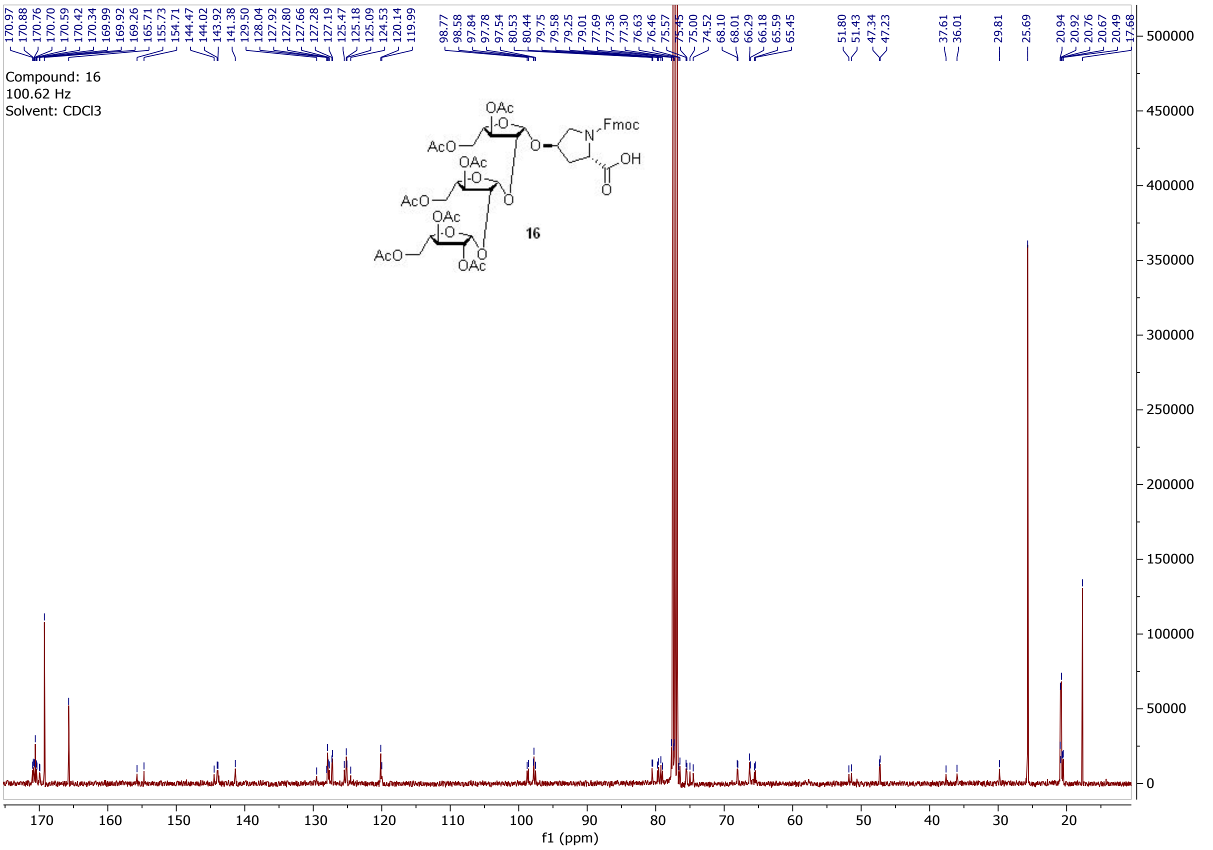

Figure S49. ^{13}C spectrum of Fmoc-[Ara(OAc)]$_3$Hyp-OBn (**16**).
